# Smart and Multi-Functional Magnetic Nanoparticles for Cancer Treatment Applications: Clinical Challenges and Future Prospects

**DOI:** 10.3390/nano12203567

**Published:** 2022-10-12

**Authors:** Elham Aram, Masome Moeni, Roya Abedizadeh, Davood Sabour, Hamid Sadeghi-Abandansari, Jabbar Gardy, Ali Hassanpour

**Affiliations:** 1 Department of Cancer Medicine, Cell Science Research Center, Royan Institute for Stem Cell Biology and Technology, ACECR, Babol 47138-18981, Iran; 2 Department of Polymer Engineering, Faculty of Engineering, Golestan University, Gorgan 49188-88369, Iran; 3 School of Chemical and Process Engineering, University of Leeds, Leeds LS2 9JT, UK; 4 Department of Cell Engineering, Cell Science Research Center, Royan Institute for Stem Cell Biology and Technology, ACECR, Tehran 16635-148, Iran

**Keywords:** smart magnetic nanoparticles, theranostics, cancer

## Abstract

Iron oxide nanoparticle (IONPs) have become a subject of interest in various biomedical fields due to their magnetism and biocompatibility. They can be utilized as heat mediators in magnetic hyperthermia (MHT) or as contrast media in magnetic resonance imaging (MRI), and ultrasound (US). In addition, their high drug-loading capacity enabled them to be therapeutic agent transporters for malignancy treatment. Hence, smartening them allows for an intelligent controlled drug release (CDR) and targeted drug delivery (TDD). Smart magnetic nanoparticles (SMNPs) can overcome the impediments faced by classical chemo-treatment strategies, since they can be navigated and release drug via external or internal stimuli. Recently, they have been synchronized with other modalities, e.g., MRI, MHT, US, and for dual/multimodal theranostic applications in a single platform. Herein, we provide an overview of the attributes of MNPs for cancer theranostic application, fabrication procedures, surface coatings, targeting approaches, and recent advancement of SMNPs. Even though MNPs feature numerous privileges over chemotherapy agents, obstacles remain in clinical usage. This review in particular covers the clinical predicaments faced by SMNPs and future research scopes in the field of SMNPs for cancer theranostics.

## 1. Introduction

Cancer is a serious burden of disorder which has become one of the greatest dilemmas to tackle globally [1]. As stated by GLOBOCAN 2020, the World Health Organization and the American Cancer Society Database for 36 cancer types in 185 countries, there has been an approximated 19.3 million new incidences in 2020. The death rate of cancer is about 10.0 million new cases each year, with the lung carcinoma in the lead, followed by colorectal, liver, stomach, and breast carcinoma, (18%, 9.4%, 8.3%, 7.7%, and 6.9%, respectively). It is predicted there will be about 28.4 million new annual cases by 2040 worldwide. The rise of about 47% in cancer cases from 2019 to 2020, with a significant expansion in developing nations (64% to 95%) compared to advanced nations (32% to 56%) could be subsequently ascribed to globalization and the economy [2,3,4,5,6,7]. The main risk elements of carcinoma formation are genetic and epigenetic modification [8,9]. Epidemiological reports have highlighted that 35% of the mortalities are due to mode of living, e.g., smoking, alcohol, unhealthy diet, repetitive application of solarium/tanning equipment, or subjection to chemical poisoning, infectious agents, or radiation [10,11].

Despite gaining exceptional knowledge about the initiation, progression, and resistance to treatment, our failure or incapability to permanently cure metastatic cancer indicates an inadequate understating of its intricacy [12,13]. Anti-neoplastic medicines often display limitations such as rapid elimination, poor efficiency, and water solubility [14,15]. Many agents which are effective in vitro have proven to be ineffective in vivo, creating an immense toxicity in healthy cells [16,17]. Substantial works are in progress to defeat drug-resistance barriers, for example the evolution of nanoparticles (NPs) could surpass the traditional chemo-medicines whilst at the same time offering diagnosis, prognosis, and treatment options [18,19,20]. A branch of NPs, magnetic nanoparticles (MNPs), have emerged as a substitute novel strategy for the treatment of neoplasm in a targeted fashion [21,22] and efforts are focused on generating smart magnetic nanoparticles (SMNPs). SMNPs can alter their structure and functional characteristics in response to an extrinsic stimulus, e.g., magnetic field (MF), magnetic hyperthermia (MHT), radiation, and ultrasound (US), and can also perform as a multi-functional tool in a single platform [23].

The category of MNPs is composed of metal (e.g., Fe, Ag, Au, Co, Ni), metal oxide (e.g., γ-Fe_2_O_3_ and Fe_3_O_4_), alloys (e.g., FePd), and ferrites (e.g., CoFe_2_O_4_) [24]. MNPs have unique biological and physiochemical characteristics as opposed to other NPs [25]. The distinctive attributes, such as a larger specific surface area-to-volume ratio, stable signals in MRI, small particle size (NPs > 200 nm and <10 nm will be removed by reticuloendothelial system (RES) and basal laminar cells, respectively), and unique magnetic characteristics (manipulable magnetic moment and magnetic sensitivity) [26,27], make them an ideal candidate for theranostic purposes.

Amongst MNPs, iron oxide NPs (IONPs) especially magnetite (Fe_3_O_4_) have been widely scrutinized in the medical fields. IONPs can reach the malignant tissue/cells in a (i) passive manner, e.g., by the enhanced permeability retention effect (EPR), (ii) an active manner by applying ligands, specific-cell-targeting, and (iii) an extraneous manner where an external stimulus, e.g., US controls the cellular uptake and the release of neoplastic cargo. One of the challenges of using IONPs is that they tend to agglomerate because of their larger surface area-to-volume ratio and dipolar coupling. The alterations with biologically compatible materials can prevent agglomeration and improve their stability, biocompatibility, dispersibility, biodistribution, and blood circulation time (BCT) [28]. Nevertheless, the undesired content release still remains as a significant hurdle in the drug delivery system (DDS) [29,30]. Recently, numerous stimuli responsive smart MNPs have been engineered to deliver therapeutic cargo in response to any stimulant including pH, temperature, redox, MF, etc. [31,32,33]. Their advantages include potential higher drug accumulation in targeted organs, prolonged BCT, enhanced systemic stability, decreased toxic side effects towards normal cells, and improved therapeutic efficacy [34,35]. However, their safety, large-scale manufacturing challenges, cost-effectiveness, and poor perception of disease heterogeneity in the patient population constrains their clinical translation [36]. Herein, we provide a critical review of the recent advances in the utilization of IONPs in biomedical fields. Attention is devoted to smart IONPs that are contemporarily under clinical investigation. Finally, targeting schemes, biological effects, and the major obstacles for the clinical trials of smart IONPs are reviewed and discussed.

## 2. Synthesis of MNPs

The research is still ongoing on the development of a suitable pathway to generate desired IONPs with productivity in clinical field with both diagnostic and therapeutic effects. Numerous strategies have been followed to fabricate particles with a high stability, monodispersity, and crystallinity via physical, biological, and wet chemical techniques. The section below describes some of the approaches in brief.

### 2.1. Biological Synthesis

Biological synthesis is an economical, energy efficient, and non-toxic strategy which can fabricate chemically stable IONPs using biotic resources [37]. Examples of reported bio-synthesis methods are (a) plant-mediated bio-synthesis of MNPs [38,39], and (b) microorganism-based bio-synthesis of MNPs which includes (i) bacteria [40], (ii) yeast [41], (iii) algae [42,43], and (iv) fungi [44,45].

The plant-mediated pathway is based on co-precipitation via the reduction of iron ions in the presence of a plant extract acting as a reductant/capping agent. Although green co-precipitation fabricates biocompatible particles with diverse shapes (elliptical rode, cube-spherical), its major disadvantage is poor size control, low crystallinity, and poly disperse particles [46,47,48].

The fabrication of MNPs using microorganisms can feasibly be cultivated in artificial lab conditions, reducing inorganic substances into NPs via extracellular or intracellular pathways [49]. The bacteria-mediated intracellular process uses the cellular machinery of bacterial cells to generate NPs, in which the positively charged metal ions are reduced by enzymes and trapped inside the cell membrane of negatively charged bacteria cells. The NPs then diffuse out of the cell membrane into the solution [50], while, in the extracellular pathway, the enzymatically reduced metal ions accumulate on the outside of the cell membrane surface [40].

Fungi-mediated synthesis is a mycosynthesis method which is carried out similar to bacteria-mediated intracellular process via extracellular and intracellular pathways. However, it has several advantages over bacteria, such as (i) simple processed, maintained, and improved cultures, (ii) reduced toxicity [51], (iii) increased bioaccumulation of metabolites, and (iv) high capacity and tolerance to metal uptake [52].

Yeast-mediated synthesis is also a mycosynthesis method with a feasible mechanism. Yeast contains an envelope/plasma membrane which can form microcapsules, encapsulating polymer NPs. The process only involves water, yeast cells, and reagents with no need for stabilizers [53]. Intracellularly yeast-generated MNPs can develop through the reduction of metal salts due to nucleophilic and redox conditions; (a) passive transport/diffusion of aqueous metal salts across the cell membrane, (b) elimination of extracellular salts, and (c) diffusion of reducing reagents into the cell [54].

Algae are also suitable candidates for the bio-synthesis of MNPs, due the fact they are hyper-accumulators (ability to uptake metal), with an easy harvest, low energy input, and economical mass-production [55,56]. The algae intracellular mode of synthesis is the least convenient while the extracellular route is more favored because of the ease of purification [57]. The physio-chemical parameters, e.g., temperature, pH, concentration of metal salts and substrates, have an impact on shape, size, and aggregation of MNPs [58].

The preparation of MNPs via bio-synthesis eliminates the need for toxic materials and is a sustainable process. However, the majority of research works have reported that MNPs produced via the bio-synthesis route exhibit a low magnetic response and a broad size distribution with a low yield [59]. Therefore, there is still scope for further improvement.

### 2.2. Physical Synthesis

The physical approach can obtain a high yield in a short time. It comprises of “Top down” and “Bottom up” techniques. In the Top-down approach, the size of MNPs is minimized to nanometers in processes such as milling and physical vapor decomposition [60,61]. In the Bottom-up technique, MNPs are condensed from the gas or liquid state, using laser evaporation [62], electrochemical [63], gas/liquid phase [64], ultrasound-assisted [65], and laser ablation [66].

The major hurdle for the physical approach is the lack of ability to produce particles with a favorable shape or size [67,68]. Moreover, the construction of IONPs with an efficient coating which provides ideal efficacy in vitro and in vivo utilization is challenging. Other obstacles such as toxicity, scale up, and concern regarding the safety of mass production makes these routes disfavored [69].

### 2.3. Chemical Synthesis

The most prevalent preparation procedure of MNPs is based on wet chemical techniques. In the following sections, we focus on the major wet chemical methods.

#### 2.3.1. Co-Precipitation

MNPs are synthesized via the simultaneous precipitation of ferrous (Fe^2+^) and ferric (Fe^3+^) salts in an aqueous media under alkaline conditions and low temperature [70,71,72,73,74,75]. It is a popular route in biological applications due the fact it is water-based with non-toxic adducts and mild experimental conditions (temperature < 100 °C) [76,77]. It is also cost-effective which enables rapid large-scale production. For example, it is used to prepare Feridex, Combidex, and Resovist contrast agents for MRI. Poor crystallinity, irregular sphere morphology, and large polydispersity because of the wide size distribution could be barriers for their clinical use [67]. To overcome these issues, parameters such as pH, reaction temperature, concentration of Fe salts and base, mixing method, and stabilizing agents such as surfactants and polymers should be controlled [78,79].

#### 2.3.2. Thermal Decomposition

An effective pathway to generate monodispersed IONPs with a small particle size distribution, high yield and crystallinity, and controllable shape and morphology (cube sphere) is by thermal decomposition. Involving a non-magnetic precursor, iron carbonyls/iron acetylacetonates which are thermally decomposed into metal in high-boiling-point organic solvents and surfactants, e.g., oleic acid (OlA), and fatty acids and an inert gas [79,80,81]. The synthesis route is costly, too complex, lengthy (hours/days), unsustainable, and needs a high temperature (300 °C). The chemicals used in the procedure are toxic, facing extreme control by regulatory agencies [76,82]. Furthermore, the final MNPs are insoluble in water; thus, post-synthesis treatment, e.g., purification/hydrophilic modification of MNPs is required prior to their applications in the biomedical field. In addition, the NPs synthetized in this way possess poor magnetic characteristics [83].

#### 2.3.3. Hydrothermal Synthesis

A bottom-up strategy for cultivating IONPs with high crystallinity is hydrothermal synthesis in which aqueous iron precursors solution are heated with elevated pressure (>2000 psi) and temperature (>200 °C) in a Teflon-lined stainless-steel autoclave. The size growth impediments faced in co-precipitation are resolved, since the high temperature can augment the growth of MNPs and prevent secondary crystallization [84]. Although it is cost-effective and eco-friendly, controlling the size of NPs is a laborious task, and the products have a broad size distribution [67]. The procedure fails to uniformly coat all of the MNPs; hence, preparing the colloidal suspension is arduous due to the aggregation of the particles. The mentioned drawbacks limit its applicability for biological purposes [84].

#### 2.3.4. Microemulsion

The dispersion of water and oil in the presence of a surfactant is microemulsion with an ability to tune NPs’ constitution, shape, size (narrow size distribution), mono-dispersity, and magnetic characteristics (e.g., saturation magnetization is critical in bio-applications). Changing the size of the droplet radius and concentrations of precursors can optimize the particle size [59,85]. However, its yield is low in comparison to thermal decomposition and co-precipitation techniques, requiring large amounts of solvent which restricts large scale production [86].

#### 2.3.5. Polyol

Polyol is an easy, single step approach which was developed to control the agglomeration of IONPs and generate monodisperse and water-soluble particles. The standard procedure consists of reducing Fe precursors using a polyol solution, e.g., diethylene glycol at an elevated temperature (>200 °C) and suitable capping media, e.g., polyacrylic acid at a basic pH. IONPs with controlled-size were obtained via the pyrolysis of metal–fatty acid salts in which the concentration/length of the fatty acid was modified. The result revealed that the consumption of high concentrations of ligands led to the formation of almost monodisperse nano-crystals [82,87,88].

### 2.4. One-Pot Synthesis of MNPs

The one-pot preparation strategy has emerged as a robust, efficient, and atom-economical (time and chemical resource saver), pathway for the fabrication of MNPs without refining the intermediate materials or the need for a separation process [84]. Some of the pioneering studies are briefly highlighted here. Wang et al. [89] prepared zwitteronic 99mTc (ZW)-doped ultra-small IONPs as T1 contrast media for MRI and single photo emission computed tomography (SPECT) via one-pot synthesis co-precipitation. The one-pot synthesis pathway produced ZW-modified IONPs with no surface functionalization restrictions, and with an ability to resist against the generation of protein corona, a decreased RES uptake, and an improved malignancy contrast and SPECT/T1 MRI signals [89]. Similarly, Yoo et al. [90] introduced the one-pot polyol synthesis for the preparation of IONPs conjugated with amine for fluorescence and MRI. The resulting MNPs were stable and efficient for T2-weighted MRI applications [90]. In addition, dual-responsive MNPs functionalized with poly (vinyl alcohol) and polymer chitosan hydrogel through one-pot synthesis demonstrated controlled Luotonin (anti-cancer medicine) delivery [91]. One pot synthesis seems promising for the preparation of multi-modal IONPs due to the fact it is fast and mild with reduced harm not only to users but also to the environment. It follows green chemistry by overcoming the issues faced during the chemical preparation.

## 3. Surface Coating

**Figure 1:**
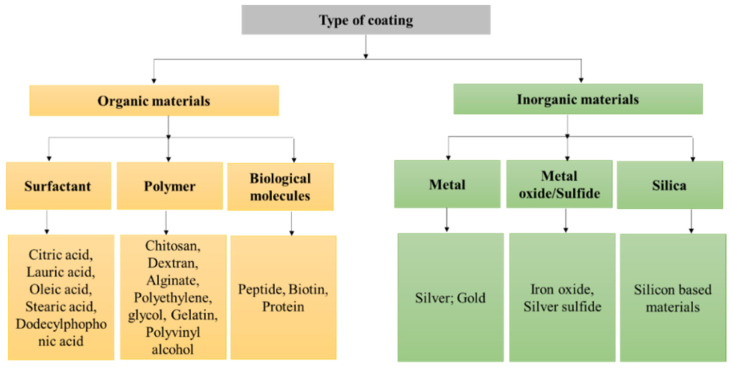
Types of materials applied as coating agents for MNPs.

The design of MNPs with a small particle size, controllable shape and morphology, high crystallinity, and superparamagnetic characteristics are vital for better biological activity and stability in the system, otherwise they can encounter obstacles such as toxicity, aggregation, and precipitation [92]. The coating limits non-specific interactions and uptake by the mononuclear phagocyte, enhances water dispersibility, prohibits possible oxidation, and provides chemical functionality for the addition of bioactive molecules, e.g., DNA, protein, or antibody [93]. MNPs can be coated either during synthesis or post-synthesis by surface adsorption or end-grafting. For surface adsorption, the coating agent forms a shell that uniformly encapsulates the core, while in the end-grafting approach, functional groups (amine, carboxyl, hydroxyl) are clamped onto the surface of the MNPs forming brush-like extensions [92,94]. The materials applied as coating agents are generally organic materials such as surfactants or inorganic compounds such as metals and oxides [67], which are summarized in Figure 1.

### 3.1. Inorganic Coating

The application of inorganic coatings such as gold, silver, or silica can improve the functionality and stability of MNPs in an aqueous solution. For instance, coating IONPs with gold can provide many advantages due to the unique characteristics of gold, such as magnetism, low toxicity, a capability to react with biological molecules, and surface plasmonic resonance which can facilitate optical features [95,96].

Si coatings have proven to be highly biocompatible and chemically stable in an aqueous environment [97,98] and have received Food and Drugs Administration (FDA) approval, e.g., food additives [99]. Si shell prevents oxidation and erosion at the same time reduces the cytotoxicity of IONPs [100,101,102]. For example, Si-layered Fe_3_O_4_ did not produce a major toxicity effect to osteoblast cells and also did not modify the secretion of collagen by cells. In addition, shielding superparamagnetic IONPs (SIONPs) with Si reduced the deterioration of the core, subsequently extending practice in MRI utilization [103]. Nevertheless, there is spreading apprehension regarding their toxicity to the immune cells. Some studies identified the toxicity induced by Si-NPs to monocytes [104], microglia [105], and Kupffer cells [106] which are all size dependent. The immunotoxicity to organs was also assessed by the intravenous administration of Si-NPs which raised the abundance of mast cells in the lung [107] and heart [108]. Oxidative stress [109], pro-inflammatory effects [110], and autophagy [111] are recognized as fundamental systems provoking immune toxicity. Efforts have been made to minimize the toxicity, induced by Si. Park et al. [112] who developed a simple and efficacious pathway to graft Si-NPs with a purified protein layer to alleviate intrinsic immune responses [112].

### 3.2. Organic Coating

The application of organic materials to coat IONPs such as polyethylene glycol (PEG) and dextran (Dex) has gained high interest amongst other polymers and organic materials. They are regarded as safe agents, and will not be quickly identified by macrophages in the liver/spleen and have longer BCT. Although, the direct cytotoxicity of Dex has not been reported, its degradation may have a direct effect on specific cellular processes [67,113]. In addition, biopolymer chitosan is a non-toxic, biocompatible, biodegradable compound, and is viewed as a sustainable and economical material. Plus, it has immense chemical structural possibilities, e.g., its hydroxyl and amino groups can form complexes with Fe_3_O_4_ NPs, increasing the hydrophilicity, stability, and biocompatibility of IONPs [114].

Surfactants can form nanocomposites with IONPs, making them sensitive towards external stimuli/internal, e.g., MF, electric fields, optical sources. The utilization of surfactants during the preparation process of IONPs facilitates a suitable coating and de-aggregates the particles. For example, the attachment of citric acid on the surface of IONPs during physical gas-phase synthesis relatively decumulated the particles [115]. In addition, the encapsulation of IONPs by surfactants can control their content release [116,117]. OlA as a capping agent can form a hydrophobic coating and its polar end can bond to the surface of IONPs, forming strong monolayer nanocomposites that can increases the consistent dispersion of MNPs in a polymer matrix of surfactant solution [118,119]. Furthermore, Kockar et al. [120] investigated the effect of tartaric and ascorbic acids as biocompatible surfactants on the characteristics of SIONPs. The surfactants increased the magnetic saturation but remained superparamagnetic, thus holding potential for biological utilization [120].

In addition to classical IONPs surface-coating agents, stimuli-sensitive/smart polymers have been designed to have fast physiochemical transitions in the surrounding tumor microenvironment (TME). Their smart chemistry is highly appealing to fabricate SMNPs since it allows a controlled and targeted distribution of pharmaceutical cargo at TME [121,122]. They can form conjugations or complexes, or become attached to biologically active molecules, e.g., nucleic acids, proteins, peptides, and carbohydrates for the purpose of wound-healing, tissue regeneration, and neoplastic medicine [123,124,125]. For example, a small hydrodynamic-size citric acid coating improved the heating efficacy of IONPs, equipped it as heat mediator in MHT, and reduced the particles’ aggregation while increasing the magnetization saturation [115].

Other prototypic examples of stimuli-responsive/smart polymers used to coat IONPs are poly(N-isopropylacrylamide) (PNIPAAm), poly(N,N-diethylacrylamide) (PDEAAm), poly (acrylic acid), and hyaluronic acid (HA) etc. [126,127].

## 4. Stimuli-Triggered SMNPs

SMNPs are sensitive to the differences between the intra/extracellular surroundings of malignant cells and are smart enough to implement structural transitions in response to a stimulant [128]. They could be equipped with coating agents/materials with sensitive linkers that have an innate sensitivity to the internal triggers in TME such as redox, concentration, pH, and enzyme levels [129]. Moreover, exogenic stimuli, e.g., light, heat, MF, and US can restrict early cargo release and facilitate an effective site-specific agent release [130]. The most common stimuli-responsive functional groups are collected in Table 1.

In the following sections, different categories of stimuli-sensitive (pH, redox, enzyme, light and ultrasound, dual/multi-modal stimuli) SMNPs are described in detail.

### 4.1. Thermo-Responsive MNPs

Temperature is a vital element to vent the drug into the TME which has a temperature greater (~40–42 °C) than healthy tissues (37 °C) [131]. Thermo-responsive (TR) SMNPs are engineered via incorporation with polymers that can perform a volume phase transition at a critical solution temperature (CST) [132]. Polymers with a lower CST (LCST) have a reduced solubility when heated, whilst upper CST (UCST) polymers act in the opposite way [133]. The UCST polymers demonstrated a higher rate of agent release in response to slight temperature variation and seem promising for photo-thermal utilization. However, their control is challenging, while LCST polymers have minor adverse effects, enhanced therapeutic efficacy, and low drug doses needed [134,135,136]. For example, PNIPAAm, a TR polymer, can transit between the hydrophilic state and hydrophobic state at LCST (hydrophilic below LCST and hydrophobic above LCST) [137].

Mainly, the MNPs are constructed to maintain their payloads in a physiological temperature and deplete upon exposure to higher temperatures. The delivery of therapeutic cargo could be performed either via (i) thermo-sensitive drug carriers, releasing the drug in response to temperatures above the physiological temperature which is an intrinsic characteristic of malignancy cells/tissues (internal stimuli) or, (ii) the malignancy cells/tissues could be heated by an external stimulus such as MF, light, etc., to enhance the release of the pharmaceutical cargo [138,139].

In this regard, Ferjaoui et al. [140] synthesized TR IONPs carrier coated with 2-(2-methoxy) ethyl methacrylate and oligo (ethylene glycol) methacrylate TR co-polymer for the sustained release of doxorubicin (DOX). The results showed 100% drug release after 52 h at 42 °C (LCST at 41 °C). The cytotoxic tests unveiled that the core/shell of IONPs had high toxic effects on human ovary carcinoma SKOV-3 cells at a very low drug concentrations [140]. Moreover, Zhang and his colleagues [141] designed nano-in-micro TR micro-spheres theranostic tools for HT and chemotherapy in cultured Caco-2 and A549 cells. In vitro, the results revealed the chemo-agent, methotrexate (MTX) or 5-fluorouacil (5-FU), had a slow release and the release of the microspheres was over the range of 37 to 43 °C, and the relaxivity (r2) value was distinctive at temperatures between 35 and 46 °C, which approved the particle characteristics as TR [141].

Although TR MNPS are deemed as low-risk and capable of efficiently loading and discharging therapeutic cargo when heated, they have not been effectively tailored to meet the clinical context. They remain unable to be induced in real-time and at the location of malignancy [142].

### 4.2. Magnetic-Responsive NPs

Magnetic field is a non-invasive energy. The revolution in nano-medicine has endorsed magnetic fields for cancer theranostic applications, including targeted drug release (TDR), MHT, and MRI. The external static or dynamic MF can apply a force greater than the blood flow force to drag drug-carrying MNPs through the complex physiological system and deliver the cargo to the site of malignancy. MF regulates the motion of MNPs and facilitates controlled and TDR. Magnetically guided pharmaceutical cargo delivery has a high therapeutic efficacy and low toxicity [143]. However, one of the limitations of MF-guided delivery is that MNPs are unable to hold maximum magnitude inside the physiological system when they are further away from the external magnetic force. Although this prevents the tumor from being targeted in the deeper region, externally magnetic-guided cargo delivery remains more effective in comparison to passive targeting (EPR effect) [144].

#### 4.2.1. Targeted Drug Release

IONPs can efficiently transport and selectively release pharmaceutical cargo with fewer side effects at TME via an external MF, and this is one of the critical fields of research in DDS. Special consideration should be given to the pharmacokinetic and in vivo characteristics of the generated IONPs and the exerted magnetic force [145]. The potential use of IONPs as a DDS to deliver DOX to a glioblastoma cancer (GMC) site guided via an external MF in a rat was investigated by Lee et al. [146]. In this drug delivery methodology, the N-hydroxy succinimide (NHS), PEG and free thiol (SH) (NHS-PEG-SH) were conjugated to modify the surface of IONPs and improve the particles’ EPR effect on GMC cells. The presence of an external MF increased the local concentration of IONPs within the GMC cells which improved the retention and accumulation of the DOX [146]. Wang et al. [147] designed a biocompatible nano-carrier with a uniform size distribution for in vivo application based on IONPs guided via an extraneous MF source. The nano-carrier demonstrated successful TDR via an extraneous MF to the rat brain, and was proven to have a potential for therapeutic application in the therapy of brain disease [147].

Magnetically TDR is promising strategy to guide the therapeutic cargo to the specified sites. Therefore, in order to succeed, an appropriate magnetic system (e.g., MF and MNPs) is a prerequisite.

#### 4.2.2. Magnetic Hyperthermia Application

Hyperthermia (HT) involves increasing the temperature of carcinoma cells (clinical temperature 42–46 °C) above physiological condition (37 °C), to induce the apoptosis/necrosis of neoplasm cells [148]. The neoplasm cells are sensitive to heat oscillations compared to healthy cells due to the lower blood supply around the tumor [149]. HT is actively utilized in pre-clinical and clinical trials as an adjuvant to treat numerous solid malignancies [150].

HT can fabricate heat via various techniques including alternating magnetic field (AMF) [151], high intensity focused ultrasound (HIFU) [152], and water bath [153]. Nonetheless, water bath fails to maintain spatially precise treatment, likewise HIFU demonstrates an inability to perform deep thermal treatment to a large specific location or ingress bones and air, while AMF can exhibit a deeper penetration competence with a higher location accuracy [154]. Since SIONPs endow a magnetocaloric effect, the exposure of SIONPs to strong frequency AMF generates heat via hysteresis loss and the heat is applicable in MHT. The technique harnesses the heat-releasing characteristics of remotely controlled SIONPs which are designed to be smart and can heat up to 42–45 °C [155,156,157]. HT can induce therapeutic cargo release by influencing the permeability of malignant vasculature, expanding the pore size of the endothelial membrane, rising perfusion, and enhancing the accumulation and toxicity of the therapeutic agent [158,159].

In clinic, the procedure can be classified into local (application of heat to small part via micro/radio waves, or US), regional (large part of the body is heated), and entire body HT [160]. The factors which impact the heating efficiency of SIONPs include concentration, magnetic characteristics, curie temperature ~50 °C, and the applied field (e.g., frequency/amplitude) [161]. 

MHT has shown capacities in sensitizing malignancy cells to adjuvant treatment, and its applicability holds huge promise, qualifying for further consideration not only as an adjuvant but also as tumor ablation technique.

#### 4.2.3. Theranostic Application of MRI and MNPs

Magnetic resonance imaging is a non-invasive and non-destructive diagnostic imaging modality that utilizes a powerful radio frequency (RF) electric field and a magnet field to visualize detailed images of the internal anatomy of human/animal. It allows the clinicians real-time monitoring of the treatment and location of malignancy as well as providing a handle to control the maneuver of therapeutic cargo and to regulate the dosage for optimum treatment results. Its superiority is related to the great spatial resolution, the contrast of sensitive tissue, and practicability in early diagnosis of malignancy which maximizes the chance of treatment and survival [162,163,164]. MRI contrast media are distinguished by their relaxivity (r1/r2) which reflects on how the medium can enhance the magnetic resonance (i) longitudinal relaxation time (T1) and (ii) transversal relaxation time (T2) in milliseconds (ms). The correlating longitudinal and transversal relaxation rates are r1 and r2, respectively, in which r1 = 1/T1 and r2 = 1/T2, and the unit is 1/ms. T1 contrast agents generate lighter/positive images whereas T2 contrast media produce darker/negative contrast images. The performance of a contrast media substantially relies on r1 and r2, which determines if there will be going to be T1 or T2-weighted images [165,166].

Currently, the heavy paramagnetic metal, Gadolinium (Gd), is broadly applied in clinic for diagnostic intention as a T1-weighted MRI contrast media, due to its strong magnetic moment, high relaxation time, and low r2/r1 ratio [167,168]. The toxicity of free Gd can be eliminated to some extent by terminating free Gd^3+^ ions using organic chelates (e.g., diethyl-enetriaminepenta acetic acid) [169]. Although a Gd-consisting-contrast-medium (GdCCM) is widely applicable in clinic, it has a varied BCT, and compelling evidence has shown that the repeated dose of GdCCM and in particular the less stable GdCCM accumulated in the globus pallidus and dentate nucleus of the brain. The patients with kidney and liver dysfunction are unable to eliminate the heavy Gd complexes and the metal can accumulate in the brain and result in brain lesions [170,171,172,173]. Hence, the findings have provoked attention about their safety as contrast media and efforts have been made to discover safer alternatives.

The concept of the unique magnetism behavior of SIONPs and their biocompatibility has made them powerful nominees to be utilized as contrast media [174,175,176]. Ultra-small SIONPs (≤5 nm) demonstrated an encouraging performance as T1 contrast media since they possess a larger surface-to-volume ratio, expanding the accessibility of surface of iron ions to the neighboring water or hydrogen [177,178]. They are used as blood-pool contrast media for magnetic resonance angiography and perfusion imaging [179]. Although SIONP T1 contrast media (≤5 nm) have highly favorable properties, the reproducibility in mass production and the complexity of the interrelated factors impact their enhancement and make their fabrication/utilization challenging; hence, they are yet to be approved for clinical applications [180].

Larger SIONPs (>8 nm) could predominantly perform as T2 contrast media and generate T2-weighted images due to the magnetic heterogeneity produced by their powerful magnetic moment and high signal/noise ratio [168]. However, the results of T2-weighted MRI can misguide the clinicians, because of the formation of black signals or the “Bloom Effect” phenomenon which might occur due to bleeding, or the deposition of metal (Fe) [177,181]. In addition, the IONPs might degrade and perform inversely. For example, Lu et al. [182] employed IONPs (ca 20 nm) for the diagnosis of hepatocellular carcinoma (HCC). The T2 IONP contrast agent injected into healthy mice generated darker images while in the HCC tumor-bearing mice, no T2-weighted/dark images were detected. Due to the acidic TME, in less than 40 s, T2 contrast agents de-agglomerated (~3 nm) and started to degrade, intelligently reversed from T2 to T1 contrast agents, and produced positive/bright images [182].

Wang et al. [183] engineered Enolase 1 (ENO1) functionalized SIONPs for MRI of pancreatic ductal adenocarcinomas. IONPs were coated by poly(ε-caprolactone)-grafted dextran (PCL-g-Dex) and conjugated with ENO1 antibody (average size of ENO1-PCL-g-Dex/SIONPs = 30 nm, Fe_3_O_4_ core size = of 5–10 nm, not suitable as T1 contrast media, since NPs size > 5 nm). The particles demonstrated superparamagnetism and enhanced the detection of adenocarcinoma in vivo and in vitro MRI. A significant reduction in T2 signal intensity in malignant tissue caused the malignancy to become darker, producing negative/darker images [183].

Sridharan et al. [184] constructed bio-mineral Fe-doped nano-calcium phosphate (nCP:Fe-CA) contrast media for the in vivo detection of liver cirrhotic and HCC nodules at an early stage. The intravenously administered nCP:Fe-CA (sphere, size: 137.6, r: 63 mM^−1^ s^−1^, colloidal stability: 48 h) detected the lesions as tiny as 0.25 cm, while the current clinical diagnosis limit is ~1 cm [184]. In another in vivo study, an nCP:Fe-CA stem cell label was constructed as MRI contrast media to track the embedding, migration, and bio-distribution of the therapeutic agent in the brain. The intracerebral implantation of a nano-formula in a healthy rat’s brain was highly biocompatible with an efficiency of ~87% and no effect on mesenchymal stem cells. In addition, T2 relaxation time considerably reduced from 195 to 89 ms and distinctive dark T2-weighted images were observed up to 30 days. The bio-compatible nCP:Fe-CA showed potential as a T2-weighted MRI contrast agent for monitoring stem cells in vivo [185].

From a diagnostic and therapeutic point of view, IONPs used in MRI applications have displayed optimistic results in imaging, selective TDD and CDR in particular, T2 negative contrast agents. IONPs have paved the way as a desirable choice in clinic competing to replace Gd-based contrast media. In addition, new horizons of innovation in designing T1 contrast media which have been intelligently converted from T2 to T1 contrast in acidic TME seems a promising modality for the construction of next-generation smart MRI contrast media. Nonetheless, further in vivo studies are necessary to assure their credibility and ultimate translation for clinical applications.

Thus, designing appropriate detection modalities which permit in vivo studies and real-time mapping is a vital aspect in order to enhance the practicality of MNPs and empower real translational methods.

### 4.3. Electric Field-Responsive MNP

Electric field-responsive (EFR) stimulus has revolutionized treatment, since an electric field can be exploited endogenously/exogenously for DDS [186]. Endogenous electric field are generated by injured tissue that can influence the proliferation/division/migration of cells, e.g., tissue repair after injury [187]. External electric field pulses can facilitate TDD by triggering the cell membrane permeability to allow drug entry. It can also stimulate wound healing or tissue restoration [188,189]. Electric field can be synchronized with MNPs to assist drug delivery to the desired location. In this regard, Viratchaiboota et al. [190] put to use the technology of electric field, MF, and IONPs to deliver 5-FU to ablate cancer cells. The results indicated that the therapeutic release time decreased but the diffusion coefficient rose [190]. Although the downside of electric field application for DDS is the generation of heat, even this phenomenon can be utilized in tumor-treating field therapy to treat malignancy [191].

### 4.4. pH-Responsive MNPs

The pH-responsive (pHR) IONPs are designed to detect the differences in pH environments of normal body tissues (pH~7.4), the tumor extracellular matrix (pH~6.5–7.0), and organelles, e.g., endosomes (pH~4.5–6.5) and function accordingly. The low pH level in malignancy tissue is due to the excess production of lactic acid, (particularly in endosomes) and reactive oxygen species (ROS) which stimulates the generation of Glutathione (GSH) to deal with ROS [192]. Molaei et al. [193] formulated an iron oxide nano-system, enveloped with pHR polyethyleneimine (PEI) and amphiphilic poly-maleic anhydride-alt-1-octadecene and functionalized with FA for curcumin (CUR) delivery. The characteristics of the final NPs are collected in Table 2. The drug release at the acidic condition of TME was improved as compared to physiological pH due to the swelling property of cationic PEI via proton absorption and repulsion effects between positive charges. Furthermore, the nano-system could be a prospective candidate for theranotics purposes as MRI contrast media and also CDR [193].

Glutaraldehyde cross-linked chitosan-coated IONPs were prepared and loaded with epirubicin (EPI) and temozolomide (TMZ) drugs for cancer treatment by Nalluri et al. [194]. The release of EPI and TMZ was much higher at a lower pH compared to the physiological pH because of the flexibility of the polymer network. As EPI contains an amine group formed an imine bond, this bond was sensitive to cleavage at a lower pH (4.4–6.4) with glutaraldehyde while TMZ with an amide group cannot form the imine bond. At pH 4.6, the release of EPI (94.06%) was higher than TMZ (87.68%) [194].

Overall, pHR IONPs are charge-dependent, with prolonged BCT and greater accumulation in the tumor. These nano-structures demonstrate fewer adverse effects and minimum non-selective cellular uptake, and these encouraging results acknowledge their competency in therapeutic cargo delivery and targeting the specific malignancy cells/tissue [195,196,197].

### 4.5. Redox-Responsive MNPs

**Figure 2:**
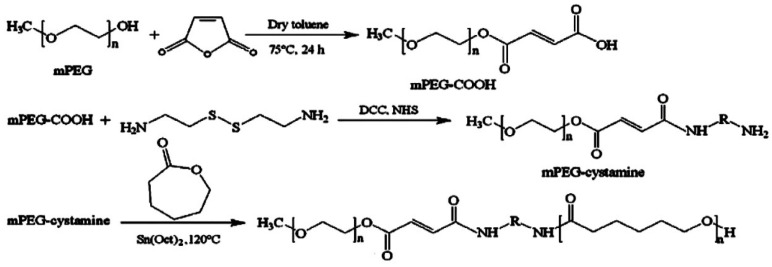
Preparation of di-block copolymer based on PEG and PCL. Reprinted with permission from [201]. Elsevier, 2018.

The redox-responsive (RR) magnetic nano-system is constructed considering the reduced TME which can perform as a unique inner signal, permitting the RR nano-system to degrade and discharge its therapeutic payload. The oxidation/reduction state of GSH and nicotinamide adenine dinucleotide phosphate (NADPH) governs the reducing TME with each having distinctive reduction capabilities [198]. Compared to NADPH, GSH has a greater concentration in reducing TME (2–10 µM) and regulates the TME via reduction in the disulfide linkage and the reaction with excessive ROS [131,198,199,200]. Mousavi et al. [201] created a di-block co-polymer based on PEG and poly(ε-caprolactone) (PCL) with SS-linkage for the co-delivery of IONPs and DOX (Figure 2). The biocompatible RR nano-carriers had a high and rapid DOX release rate in the reductive environment of human foreskin fibroblast cells [201].

In another work, the researchers produced a RR protein delivery system based on IONPs and methoxy-poly(ethylene glycol)-block-poly [dopamine diethylene triamine-L-glutamate] polymer ligands to investigate redox-triggered targeted human serum albumin (HSA) as a model protein delivery and diagnostic imaging of breast cancer [202]. The average size of nano-carriers was approximately 60–70 nm and proteins were released swiftly under a high concentration of GSH (10 µM) due to the reduction-triggered disulfide bonds cleavage. The polymer-coated particles had a low cytotoxicity and biocompatibility against HeLa cells and demonstrated an effective cellular uptake. In vivo imaging analysis of breast-tumor-bearing mice showed the nano-carriers can serve as potential T2-weighted MRI contrast media [202]. For delivering DOX and MRI, polydopamine (PDA)-based RR IONPs were constructed by Shang et al. [203]. In the presence of GSH, a sustained and accumulative DOX release (73%) was observed, while in the absence of GSH the release rate declined (37%). In addition, the IONPs exhibited intense T2-weighted signals, a negative contrast result in MRI analysis, and an enhanced r2 value [203].

A RR magnetic star-structured micellar (MSSM) was generated using magnetite and PEG and PCL co-polymers and loaded with DOX. The MSSM was modified by phenylboronic acid (PhBA) to enhance the agent’s capability to target sialic acid (SA) which is up regulated in cancerous cells, e.g., HeLa cells and HepG2 cells. The MNPs with a saturation magnetization of 15 emu/g had both active-targeting and magnetic-targeting features to accumulate around the malignant tissues and internalize HepG2 cells by the sialic acid-mediated endocytosis. Moreover, the rapid DOX release under a high level of GSH improved the therapeutic efficacy. The RR MSSM systems displayed therapeutic efficacy in targeting malignancy tissue without the premature or non-specific distribution of therapeutic cargo due to the low level of reducing species in the blood. However, these studies were conducted on animal models which are dissimilar to real conditions in malignancy cells/tissues or in metastatic carcinomas. Plus, the major concern is mass production which has remained a hurdle [192,199,204].

### 4.6. Enzyme-Responsive MNPs 

The integration of MNPs with enzyme responsive (ER) stimuli has received great interest since enzymes play essential roles in all biological and metabolic processes. Some of their advantages are substrate specificity and high selectivity, and they are capable of attaining ER drug release through the bio-catalytic action at malignancy cells/tissues [131,205]. In cancerous cells, specific enzymes, including proteases, phospholipases, lipase, or glycosidase, often exhibit a higher expression than in normal cells [206]. In recent studies, two classes of enzymes have often been used as stimulants in ER drug delivery, including proteases (or peptidases) and phospholipase [207]. For instance, Li et al. [208] fabricated mesoporous silica nano particles (MPSNPs) engulfing DOX and matrix metalloproteinase-2 (MMP-2) ER peptide for chemo-drug delivery and contrast media in MRI (Table 3). The rate of DOX release without the peptide was significantly greater; however, MMP-2-facilitated IONPs initially had a slow-release rate, and then gradually 20 min later the rate value intensely rose. The peptide on the surface of the NPs efficiently cleaved in the presence of the MMP-2 enzyme to induce DOX release. Furthermore, the results of the methyl thiazole tetrazolium assay showed that the final nano-carrier had a high specificity to HT-1080 human fibrosarcoma cells with high MMP-2 expression and limited toxicity to normal cells. The MRI results acknowledged that the exogenous MF-stimulated accumulation of nano-carriers at the tumor site improved T2 signals and r2; hence, they should be considered as candidates in a sensitive probe [208].

**Figure 3:**
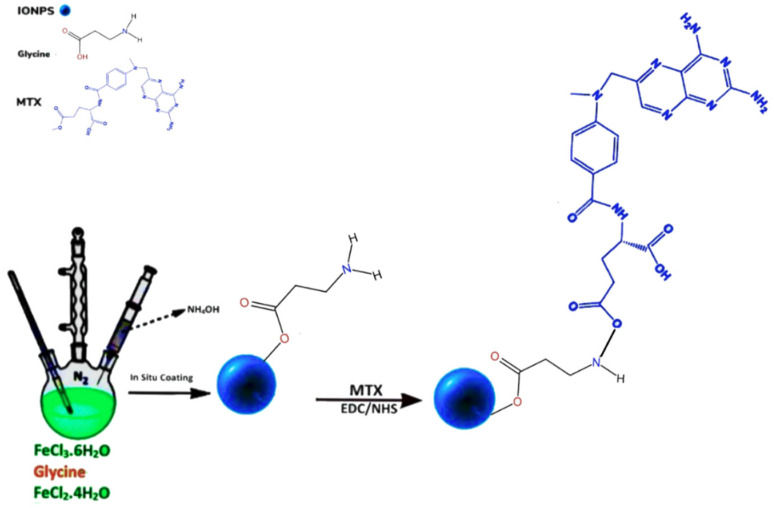
Enzyme-responsive, glycine-coated, MTX-conjugated Fe_3_O_4_ NPs. Redrawn from [209].

Similarly, Nosrati et al. [209] developed enzyme-responsive glycine-coated Fe_3_O_4_ NPs functionalized with MTX for TDD to MCF-7 breast carcinoma cells (Figure 3). The MTX was released faster since the proteinase K enzyme cleaved the peptide inside lysosomes. Furthermore, the final nano-carrier with an average size of 46.82 nm demonstrated a higher cytotoxicity on the MCF-7 cell line as compared to free MTX due to the large number of enzymes in lysosomes that cleaved peptide bonds and allowed the free MTX to decrease cellular viability [209]. Rastegari et al. [210] prepared two samples, coating one with β-cyclodextrin (β-CD) and the other with carboxymethyl chitosan (CMCS) to degrade and promote prodigiosin (PG) delivery in the presence of lysosomal glycoside hydrolases. The characteristics of nano-carriers such as size, saturation magnetization, release, and toxicity are collected in Table 4. Both nano-carriers displayed a relatively fast rate of PG release in the cells’ lysosome and had exceptionally low drug-leakage into the bloodstream. The nano-carriers targeted glucose overexpressing cells and the PG-loaded CMCS MNPs had higher toxicity effects on MCF-7/GFP and HepG2 cells and could be more effective in the killing of cancerous cells compared to PG-loaded β-CD MNPs [210].

The MNPs incorporated with enzymes display tremendous diagnosis and therapeutic potency and can embellish bio-“specificity” and “selectivity” of the nano-structures. Their site-specific and selectiveness on one hand offer significantly improved accumulation at the malignancy site and decrease the uptake of nano-formulations by non-targeted tissue, and on the other hand, facilitate site-specific CDR without undermining targeting efficacy. Plus, they can overcome constrains faced by conventional therapeutic agents. Although progress has been achieved in enzyme-responsive MNPs, there are still many limitations and drawbacks that need to be addressed, such as biocompatibility, cytotoxicity, and systemic toxicity [211,212,213,214].

### 4.7. Light and Ultrasound-Responsive MNPs

Light-sensitive (LS) MNPs operate by an exogenic light source (i.e., ultraviolet (UV), visible (Vs), US irradiation, and near infrared light (NIR)/photothermal therapy (PTT)), and their physical and chemical structures become disrupted and destabilized, releasing the agent in the desired tissue [138]. The practice of UV and Vs lights is limited owing to their short penetration depth in vivo [138,215]. The non-invasive PTT utilizes NPs to change NIR light into heat to eradicate malignancy cells, and has demonstrated unique positive results in cancer therapy [206,216]. NIR light uses an absorbing chromophore (e.g., hemoglobin) to absorb light and increases the permeability of the tumor blood vessels, causing leakage, and annihilating malignant cells without causing damage to healthy cells and with low scattering property at the wavelengths of 700 to ~1000 nm [138,206]. Hence, NIR could be more practical in biomedical utilization when it is hybridized with MNPs. The impact of NIR light on IONPs is due to the intrinsic photothermal effect of the particles and the increase in their thermal motion to discharge the therapeutic payload and cause apoptosis of malignancy cells. The hybridization of NIR light and IONPs allows the immobilization of pharmaceutical cargo at the malignancy site for precise CDR, leading to multiple therapeutic effects in a single dose [217]. Feng et al. [218] generated hollow mesoporous CuS NPs containing PEGylated Fe_3_O_4_ and DOX-loaded for utilization in NIR-responsive DDS, diagnosis, and therapy of breast carcinoma. Nano-carriers displayed a high cytotoxicity on MCF-7 cells with decreased cell viability due to the effective phototherapy and synergetic effect of IONPs. Additionally, the exposure of IONPs with NIR light enhanced DOX release and destroyed the high number of malignancy cells [218]. Eyvazzadeh et al. [219] also synthesized core–shell gold-coated IONP (Au@IONP) as an LR agent for cancer PTT. Heating the nano-complex to the desired temperature with laser irradiation induced 70% cell death [219]. In another study, methylene blue (MB) photosensitiser was immobilized on Cu-Fe MNPs which resulted in an enhanced PTT effect and damaged the tumor cells efficiently since Cu-Fe MNPs acted as Fenton catalyst, changing H_2_O_2_ into ROS, e.g., singlet oxygen (^1^O_2_)/an excited form of O_2_. [220]. 

The US-responsive stimuli have received significant attention due to their safe profile, deep penetration into the body, non-invasiveness, and capability of unloading IONPs payload at the desired sites via thermal and mechanical effects [221]. The irradiated US waves continuously fabricate micro-bubbles (MBBs) in the form of spherical pressure waves which lead to the generation of heat, micro-jets, and oxidative radicals. The non-linear oscillations of MBBs re-radiated energy in varied frequencies. The production of low frequencies (20–100 kHz) promotes the implosion of MBB which aids the release of the therapeutic payload at the malignancy site [142,222,223]. The US-responsive magnetic mesoporous silica MBBs facilitated gene delivery guided by an external MF to malignant cells/tissues. The US assisted the cargo release and enhanced the efficiency of the plasmid DNA delivery to malignant tissue via stimulation of the blood tumor barrier to open and enhanced the membrane permeability. Furthermore, the HEK293T and SKOV3 cells treated with MMPS MBBs showed better viability than those treated with only magnetic MPSNPs (M MPSNP) due to the presence of lipid MBBs [224]. The characteristics of nano-carriers are shown in Table 5.

Even though PTT which uses NIR light is capable of disrupting the scaffold of nano-carriers to induce the therapeutic agent release, the number of NIR light-absorbing chromophores are limited which restricts the progress of this procedure [225]. Additionally, US waves can be utilized to stimulate oxygen-transporting MBBs to discharge oxygen, whilst concurrently initiate a sono-sensitizer, (especially practical for treatments of hypoxic malignancies) [226]. Besides the ability of US to enhance the agent’s cellular uptake, it can minimize the off-target and non-specific effects of chemo-agents [227].

### 4.8. Dual and Multi-Stimuli-Responsive MNPs

Single/multi stimuli-triggered MNPs have been utilized not only to improve sensitivity, but also to target and release anticancer cargo efficiently at the location of interest [228]. For example, the utilization of MHT partnered with other modalities, e.g., chemotherapy and concomitant with MRI and US has been advantages. Dual/multi modal application (i) decreases the necessity of high toxic concentration, and (ii) the therapeutic temperature is obtained in less time, preventing adverse effects (such as prolonged contact with heat causes burn/pain) [229]. Pre-clinical studies of thermo-sensitive MNPs in MHT therapy for theranostic purposes are collected in Table 6. The translation of this modality into standard clinical routine in therapy of various neoplasms has limitations including the loco-regional delivery of MNPs and real-time mapping during the procedure [230].

To tackle the aforementioned obstacles, the synergetic application of US with MHT has become one of the interesting new modalities for malignancy treatment, since it can specifically target the tumor cells without having any detrimental effect on normal cells. US-stimulated MHT is non-invasive with no ionization effect. US waves cause the vibration of tissue and as a result heat is generated [231,232]. In a pre-clinical study by Hadadian et al. [233], TR MNPs were utilized, integrating MHT with magnetomotive US imaging for localizing and temperature mapping of MNPs in a phantom study. However, further in vivo studies will be required to assess the technique in more complex and viscoelastic tissue [233]. In another study, hybridizing TR MNPs with US waves and MHT increased the rate of malignancy cell destruction and also the rate of therapeutic efficiency improved. Nonetheless, low intensity US-MHT is impractical for deep-seated malignancies and organs with air, e.g., abdomen and lungs. Since the acoustic impedance fails to distinguish between air and soft tissue, there will not be transmission in cavities with air [234].

In addition, doping Fe with other metal such as Zn and Mn which possess high saturation magnetization will improve the heating efficiency of MNPs [235]. Zn and Mn dopants in low doses have distinctive characteristics such as being non-toxic to healthy cells. Albarqi et al. [236] developed a multi dopant HR magnetic nano-carrier; using Zn, Mn, and Fe. The MNP had a high saturation magnetization and enhanced heating efficacy, suitable for MHT application [233,236].

A neoadjuvant chemo-treatment protocol using DOX synchronized with mild loco-regional MHT displayed remarkable improvements in survival rate of soft tissue sarcoma patients, due to cellular modification induced by MHT, e.g., DNA repair [237]. In this regard, a number of studies employed DOX and MHT in combination therapy (Table 7) [137,238,239,240]. The decoration of carboxylate-functionalized PNIPAAm nano-gel (NG) with Fe_3_O_4_ NPs via covalent bonds generated multi-modal diagnostic imaging and a thermal therapy tool which actuated DOX release due to the affinity of Fe to the carboxylate group [137]. Under RF field, thermally triggered MNPs exhibited TDR capability, above the LCST of carboxylated PNIPAAm, LCST = 43 °C (LCST of PNIPAAm 32 °C, below body temperature). Any temperature lower than LCST will be closer to body temperature (37 °C), leading to unexpected and early agent release, likewise, above LCST can affect healthy cells and cause adverse effects [137,158,241]. In vitro studies revealed the encapsulation of DOX by magneto-liposome (thermo-responsive agent) conjugated with ferumoxytol used in MHT and drug delivery could be a powerful modality for in vivo carcinoma treatment [238]. Additionally, Pourjavadi et al. [242] used N-isopropylacrylamide (NIPAM) for the TR release of paclitaxel. The therapeutic payload release ameliorated at an elevated temperature, indicating the agent release is temperature dependent [242]. In addition, Gue et al. [239] Pramanik et al. [240] and Afzalipour et al. [243] fabricated TR MNPs, grafted with overexpressed receptor targeting functional groups, MTX, HA, and FA, respectively, for application in oncothermia (Table 7).

Furthermore, pH and heat responsive Fe_3_O_4_ NPs conjugated with sodium dodecyl sulphate, aniline hydrochloride, and CUR were synthesized for CDR and MHT in vitro and in vivo studies. The rode and worm shape magnetic micelles demonstrated high colloidal stability (surface charge: −31 mV), great drug-loading affinity, satisfying heat efficiency, and high magnetization [244]. Matos et al. [245] developed Fe_3_O_4_ electro-spun nano-composite, functionalized with cellulose acetate, OlA and dimercaptosuccinic acid. The pH and heat sensitive, spherical particles had a high heating capacity due to the adsorption of IONPs on the surface of fiber. They also exhibited a high efficiency in carcinoma treatment with lower adverse effects [245].

The summary of the studies based on application of MNPs in MHT therapy and CDR for theranostic purposes are collected in Table 7.

Moreover, Farshbaf et al. [246] engineered smart theranostic agents for dual-modal MRI and TDD to A549 lung carcinoma cell, the r2 = 0.15 mM^−1^·ms^−1^ and size = 62 nm suggested MNPs have potential as a T2-weighted negative contrast agent for MRI [246]. In addition, MNP constructed by Nandwana et al. [137] had competency to enhance MRI contrast compared to clinically approved dual-modal contrast agents (MNP~8 nm performed as T2-weighted images while MNP~4 nm produced T1-weighted images) [137].

Aljani et al. [247] designed a multi-functional hybrid nano-formula, ideal for fluorescence imaging and also promising as an MRI contrast medium [247]. Additionally, Gholibegloo et al. [248] designed a smart theranostic nano-sponge for cancer treatment via the modification of Fe_3_O_4_ MNPs with cyclodextrin nano-sponges (CDNSs), FA, (CDNS-FA) and loaded with CUR for TDD and T2-weighted MRI. The nano-sponge demonstrated hemo-compatibility [248]. ETB-loaded IONPs successfully performed as smart theranostic tools and contrast probe (bio-marker) for MRI, with great targeting ability against highly aggressive and metastasizing malignancy cells [249]. Moreover, the study by Abedi et al. [250] showed that increasing the concentration of iron in dual modal imidazoline-functionalized MPSNPs in MRI formed T2-weighted images (darker images), while no alteration was detected for T1-weighted images (r1 = 5.89 m/M s^−1^, r2 = 144.88 m/M s^−1^) [250].

Ray et al. [164] developed a strategy for real-time mapping of MNPs by MRI, using Magnevist as a contrast agent and drug release by AMF heating. However, further in vivo and clinical assessments are needed to implement the strategy for application in clinic [164].

The solo application of PTT encounters challenges, such as uneven heat generation by laser beam energy and NPs, and also the gradual reduction in laser energy over time will cause an insufficient penetration into malignancy cells [251,252,253]. To overcome some of the issues, PTT can be synchronized with another technique, e.g., MRI. In this regard, sialic acid-functionalized mesoporous PDA SIONPs was designed for chemo-photothermal therapy and T1/T2 MRI of hepatic carcinoma. The increase in iron concentrations produced darker T2 images and lighter T1-weighted images, suggesting the nano-formula could be a potential candidate as T1/T2 dual-modal MRI contrast media [180]. In another study, arginylglycylaspartic (RGD) peptide-conjugated NBs were fabricated high relaxation value, T2-weighted MRI and ultrasound promoted the simultaneous diagnosis and therapeutic agent release to hepatocellular carcinoma cells [253].

Licciardi et al. [254] developed IONPs coated with amphiphilic inulin-based graft-copolymer as smart theranostic tools for MRI and TDD (FA conjugation permits active targeting) of DOX to colon carcinoma cells. The lipoic acid (LA) was employed as cross-linking ligand to link the polymers and to provide redox-sensitivity characteristics to stimulate CDR, due to the S-S bond which resulted in the cleavage of bonds and disturbing the stability of the molecule and releasing the agent. [254]. Similarly, Dong Li et al. [255] functionalized MNPs with FA and loaded DOX for simultaneous MRI and TDD to gastric cancer MGC-803 cells in vitro and in vivo. The MNPs displayed longer BCT ad were used for diagnosis/detection of small malignancy cell with overexpressed folate [255]. In addition, in vivo and in vitro studies revealed the conjugation of CUR with LA on the surface of Au-Fe_3_O_4_ NPs and equipped with GSH ligands have potential for theranostics applications in TDD and as contrast media for MRI of brain carcinoma. Moreover, similar to previously mentioned studies, increasing the concentration of iron resulted in a decrease in signal intensity in MRI of astrocyte and U87MG cells [256]. In addition, Wang et al. [257] and Xie et al. [258] fabricated MNPs for theranostic utilization, including MRI, MHT and TDD [257,258]. Table 8 summarizes some studies for the application of SIONPs in drug delivery and imaging in single platform.

Furthermore, Gao et al. [259] reported temperature and redox-responsive poly (N-vinylcaprolactam) (PNVCL)/Fe_3_O_4_ NPs, transporting 5-FU for tumor targeting and MRI. The MNPs were fabricated via inverse mini-emulsion polymerization and disulfide-bond (S-S bond) containing a cross-linker. An improvement in drug release was observed due to the reduction factor as well as increases in the temperature above the LCST of PNVCL/Fe_3_O_4_ NPs [259]. The results are collected in Table 9.

**Figure 4:**
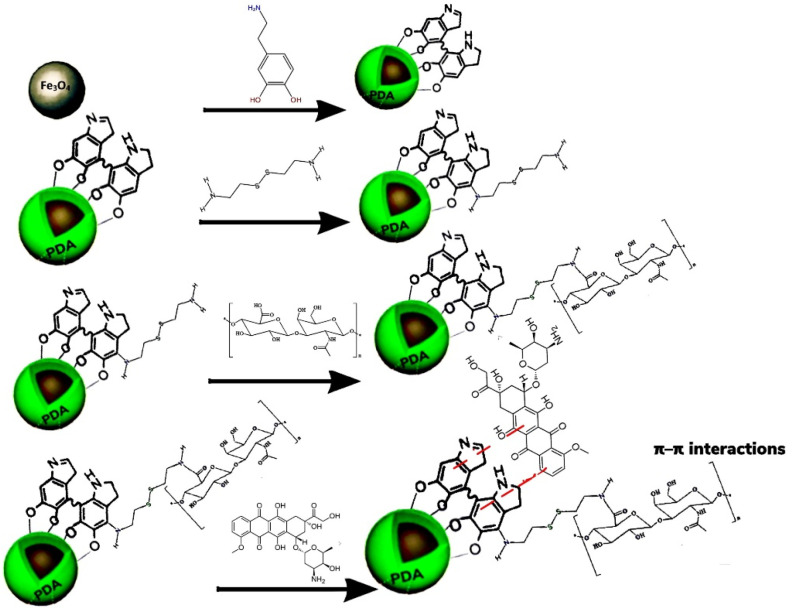
Schematic representation of a triple stimuli-responsive MNP drug carrier. Redrawn from [261].

In other research, NIR merged with pHR, smart meso-2,3-dimercaptosuccinic acid-coated, and DOX-loaded IONPs was prepared for breast carcinoma therapy. In vitro cargo release was higher due to the high solubility of the protonated DOX at a lower pH. Likewise, NIR light irradiation induced temperature rise, aiding drug release and causing death to cancer cells [260]. Also, a triple-stimuli-responsive drug carrier was developed by conjugation of HA onto the surface of IONPs-PDA through redox-sensitive S-S bond and attaching DOX via π–π interactions (Figure 4) [261].

In the presence of a GSH reducing agent, an NIR light, and a low pH, the therapeutic payload release was higher. The multi-modal-therapy displayed a positive response, the viability of HeLa cell was at 16.2%, while it was higher in single chemotherapy (55.3%)/PTT (52.1%). In vivo MRI results demonstrated an increased accumulation of nano-carriers in tumor tissue providing an enhanced contrast. The results are collected in Table 10 [261].

The dual and multi-responsive MNPs are composed of more than two types or complex targeting moieties in nano-platform, delivering the therapeutic cargo to the intended site. The targeting strategy offers versatile modes of response and smart control of DDS. Simultaneously, it can identify and react with more than one molecular participant of the pathological site, decrease off-target payload discharge, and improve therapeutic efficiency [207,262,263,264]. Nevertheless, the system confronts an enigma because of the steric deterrent causing an insufficient/plethora level of ligand density which debilitate targeting [265].

## 5. Magnetic Nanoparticle Targeting Methods

The key purpose in the diagnosis/therapeutics of carcinoma is the design of DDS with potency to target the lethal malignancy cells while leaving healthy cells/tissue intact. This might be attainable by the efficient delivery of MNPs loaded with anti-neoplastic agents into TME [213]. The successful targeting of nano-formulas depends on their ability to cross through a number of biological and physiological impediments such as unspecific interactions and early elimination from the bloodstream. The MNPs carcinoma-cell-targeting includes passive targeting via carcinoma vasculature and active targeting via ligand-receptor binding.

Passive targeting implies the assembly of MNPs in the malignant cells/tissue via EPR effect which was discovered in 1980s by Maeda and his colleagues [266]. The performance of the EPR effect is particularly defined with cancer biology, e.g., hypoxia/inflammation, which causes angiogenesis and lymphangiogenesis. Due to the rapid growth of tumors, they generate highly permeable, leaky, and defective veins which are ideal to facilitate the transit of macromolecules greater than 40 kDa and accumulation of NPs in TME. The therapeutic cargo is required to stay in the blood circulation for ≥6 h to demonstrate an effective EPR effect [267,268,269]. The poor drainage of lymphatic fluid and the irregular and leaky lymphatic vessels of the lymphatic system [270] can assist the retention of NPs, resulting in the passive targeting of the therapeutic cargo [271]. For example, Guo et al. [253] designed spherical MNPs with diameters of 160–220 nm, indicating a good candidate for passive targeting via EPR effect [253]. In passively targeted nano-formulations, the heterogeneity of solid malignancies and the lack of ability to manage the uptake of nano-carriers can minimize the therapeutic efficiency and cause multiple-drug resistance [206,272]. Another limitation is the short BCT that can reduce therapeutic efficacy [273]. Even polymerizing MNPs has not yet been able to completely resolve the issues, so further optimization and careful assessments are required.

According to recent research, prospective nano-pharmaceutics should mainly concentrate on developing nano-carriers based on active targeting which have demonstrated improved/enhanced efficacy and capable of overcoming the challenges of passive targeting in carcinoma treatment [274,275]. For example, Ghorbani et al. [276] engineered an anti-neoplastic agent by conjugating MTX on MNPs, employing both passive and active targeting mechanism. Although the results indicated MTX penetrated the cells via passive targeting strategies, due to the target-site identification by ligand (MTX) and interaction with overexpressed receptors, the cellular uptake of MTX increased. Hence, folate receptor positive malignancies were actively targeted and a high degree of MCF-7 cells were eradicated compared to MDA-MB-231 cells (Table 11) [276]. Similarly, Avedian et al. [277] designed smart MPSNPs for the active and passive targeted delivery of Erlotinib (ETB) to Human cervical carcinoma cells (HeLa). PEI coating regulated pHR CDR in various pH and the targeting agent FA, facilitated targeted cargo delivery [277].

Active targeting can unload remarkable quantities of MNPs to TME unlike free or passively targeted anti-neoplastic agents. It will enhance the specificity and affinities of the MNPs towards malignancy cells. Since MNPs are functionalized with ligands which bind to overexpressed receptors on carcinoma cells [278]. The phenomenon was initiated in 1980 by Lee and his colleagues who grafted antibodies on liposomes surfaces and conjugated with ligands [279]. The performance of targeted anti-neoplastic agent significantly relies on several factors such as (i) the nano-carrier, (e.g., size, shape, charge, stability, degradability, etc.), (ii) the ligand (availability, characteristics, density, bindings, etc.), pharmaceutical agent (type, release, efficacy, etc.), and (iii) other factors (cancer heterogeneity, type, stage, overexpressed receptors, size/density) [280].

**Figure 5:**
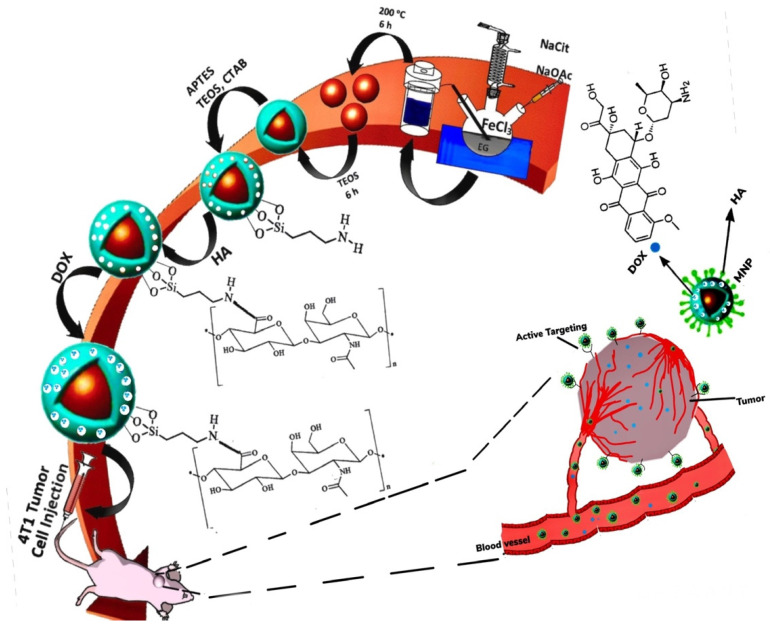
Schematic representation of an in vivo experimental procedure and the active targeting of DOX, and the cellular uptake by 4T1 cells following the administration. Redrawn from [282].

MNPs can be modified by targeting/homing agents that can actively react with overexpressed receptors on malignant cells/small molecules, e.g., carbohydrates and FA or macromolecules, i.e., antibodies, peptides, proteins, and aptamers (Apt) [281]. For instance, the conjugation of HA onto IONPs enabled selective binding to CD44 which is overexpressed on the surface of 4T1 breast carcinoma cell lines, permitting an efficient treatment. The distribution of DOX was improved at pH = 5.5 due to the protonation of DOX in the areas of higher acidity. The nano-carriers exhibited higher toxicity activity against 4T1 cells compared to GES-1 gastric mucosa cells [282]. Figure 5 illustrates the experimental procedure and active targeting of the DOX, and the cellular uptake by 4T1 cells, following the administration.

Table 11 shows the most recent examples of actively targeted smart magnetic nano-pharmaceutics delivery for cancer treatment. For instance, FA can stimulate cellular internalization of MNPs in active manner [283]. In vitro studies indicated FA promoted the targeted and controlled release of DOX in a dual release mechanism (pH and redox) [284] and a redox-responsive mechanism, respectively [283]. FA is cheap and widely available, has low immunogenicity and toxicity, also it is simple to alter for application as single/dual targeted systems in neoplasm treatment [274,281]. Nevertheless, challenges continue to persist in clinical trials for FA-targeted NPs against human malignancy cells [285].

Anti mucin (MUC) aptamer (MUC1 Apt) is also an alternative targeting agent/tumor marker and is overexpressed in the majority of adenocarcinomas on the whole cell surface and sheds in the blood system. MUC1 Apt is a highly glycosylated transmembrane glycoprotein. MUC1 Apt was used for the pHR release and active targeting of DOX in vitro. The nano-medicine demonstrated capability as a potential multi-modal agent for the simultaneous detection and treatment of MUC1 overexpressing carcinoma cells in clinical application [286,287]. Another example is PhBA, which allows selective and reversible combination with polyhydroxylated compounds which contain vicinal diol or meta-diol structure and can form covalent complexes. This feature of PhBA was employed in an in vitro study to actively target DOX release via pHR release [288]. Another targeting agent called Lactoferrin (LF), an Fe-carrier glycoprotein, was utilized to bind to overexpressed receptors on C6 glioma cells and endothelial cells for active targeting [289].

Some of the targeting agents serve dual purposes. For example, a number of in vitro studies applied MTX which is an antimetabolite agent, capable of acting as targeting and chemo-therapeutic agents. Since the structure of MTX is analogous to FA, it can target the folate-receptor-positive tumors and interrupt the metabolism pathway [209,276,290,291]. The outcomes confirmed that Fe MTX NPs are efficient anticancer delivery systems and most likely play a part in future in vivo applications. Pemetrexed (PMX), similar to MTX, is a folate analog and its application was evaluated for active targeting in vitro and in vivo by Ak et al. [292]. Moreover, the fusion of the targeting ligand to smarten IONPs could lead to a reduction in non-specificity and enhance the uptake of nano-formula, resulting in increased anticancer effects and minimized toxicity to healthy cells as compared to passively targeted alternatives. Thus, identifying the specific receptors that are abundantly overexpressed on malignancy cells and ligands which bind strongly to these receptors are vital aspects of constructing smart IONPs. Although, many pre-clinical studies have been conducted on actively targeted stimuli-responsive MNPs, no nano-formula has yet been approved by the FDA. This is due to the presence of different barriers that limit the cells penetration of nano-formula. Therefore, further research is needed for the successful production of cancer targeted IONPs in clinical applications. Furthermore, the construction of nano-formulas with efficiency in TDD and monitoring/imaging has a great degree of importance for both diagnosis/therapeutic intentions. Such smart targeted nano-formulas will have the preference to monitor TME and release the therapeutic cargo intracellularly to selectively eradicate the malignancy cells [248,250].

## 6. Interaction of MNPs with Biological System

IONPs are ideal candidates for use in clinical utilization because the force of magnetism has low physical interactions with the body [294,295]. Since these NPs are foreign entities, the administration could cause some biological responses [296]. Intravenous injection is the most common route of entry in which the body’s immune system as the first defense mechanism responds promptly and attempts to clear the particles from the blood stream [28,295].

The main part of the immune system is RES which includes monocytes circulating in the blood and specialized tissue-resident macrophages such as liver kupffer cells, bone osteoclasts, lung alveolar macrophages, and brain microglia. This system protects the body from pathogens or foreign particles such as IONPs via phagocytosis [28] and accumulating NPs in the liver and spleen [297]. Activation of immune cells and release of cytokines in the blood stream and tissues may cause systemic or local inflammation which is classified as a side effect [298]. Following the activation of immune cells such as macrophage, IONP can produce ROS and oxidative stress [299].

Moreover, the complement system is another part of the immune system that consists of a group of ~30 proteins soluble in plasma that can attach to IONPs and influence their efficacy. It triggers a series of inflammatory processes which cause anaphylatoxin production and finally cardiac and respiratory complications [300].

Different studies have reported that a variety of factors such as size, charge, surface, and polymer conformation as well as molecular structure of MNPs can influence the protein adsorption. For instance, positively charged polystyrene NPs enhanced complement anaphylatoxin levels and negatively charged citrated IONPs attached to more serum proteins and activated the complement proteins extensively [299]. When NPs enter the blood stream, macromolecules, especially proteins, may bind to their surface and produce “protein corona (PC)” [298].The PC affects the cell recognition of NPs that is called “cell vision”.

Cell responses to the NPs depend on the first contact between NP and the cell surface which differs between the protein coated NP and the intact one [295]. The bio-identity of NPs is influenced by three factors: physiochemical characteristics (shape, size, polydispersity, polymer conformation, molecular structure and etc.), biological elements (source of protein, human/animal), and experimental conditions (e.g., temperature, ionic strength) [301]. IONP tend to have a potency to attach to different plasma proteins such as immunoglobulins [295], blood coagulation, angiogenesis, complement system, and other regulatory proteins involved in protein processing, lipid metabolism, and cytoskeleton organization. For example, it has been demonstrated that IONPs attached to coagulation factor VII and fibrinogen led to the activation of the kallikrein system and induced thrombosis [300].

Moreover, there is another macrophage population called marginal zones in the spleen which are involved in blood clearance of pathogens or foreign agents such as IONPs by phagocytosis. IONP can aggregate in the liver/spleen and cause inflammation via necrosis, ROS production, secretion of pro-inflammatory cytokines [299], and lysosomal or mitochondrial damage [300]. ROS production enhancement is an initiating step which triggers an innate immune response by inflammasome activation [299]. Recently this feature of IONPs has attracted enormous interest in cancer vaccine immunotherapy as an activator of the immune system by targeting the tumor site [302].

The lymphatic system is composed of lymph nodes linked to each other by lymphatic vessels. When IONPs enter a tissue, they may move to lymph vessels as well as regional lymph nodes, where they encounter sinusoidal macrophages. Therefore, most of intravenously injected IONPs could be not only trapped by liver and spleen prior to reach any other organs but also lymph nodes, except intramuscular or subcutaneous injections in which regional lymph nodes may be the first clearance sites [28].

The renal system is another elimination pathway via the non-phagocytizing route that can clear carbohydrates, proteins, ions, and possibly NPs. Generally, the observation indicates that NPs with small sizes can be swiftly removed through the renal system [303]. However, there are no data that have reported the presence of non-degraded IONPs in urine [28].

The blood–brain barrier (BBB) is also an issue for the utilization of NPs in brain carcinomas, since only 2% of agents can cross that barrier. Size and charge of NPs should be optimized to be able to pass through BBB [28]. Additionally, the assembly of iron in the brain is linked with neurodegenerative illnesses, e.g., multiple sclerosis and Alzheimer’s [304] due to its capability to stimulate the generation of oxidative stress and ROS [299,305]. Furthermore, the correlation of magnetite NPs with microvascular endothelial cells of BBB led to detrimental implications [306].

Intrapulmonary delivery of IONPs was practiced for imaging and treatment of lung carcinoma. During the procedure IONPs enter the alveolar which has macrophages that phagocytize IONPs [28]. The interaction of NPs with pulmonary surfactant proteins raised phagocytosis [307]. Ruge et al. [308] reported that MNPs highly attached to surfactant protein A with high interaction with alveolar macrophages and maximized phagocytosis [308]. Ultimately, phagocytosis of NPs by alveolar macrophages largely relays on the generation of protein corona around the NP which can determine bio-distribution and immunological fate of the NP [299]. Moreover, the intratracheal dispensing of IONPs enormously rose the quantities of neutrophils and inflammatory cells in bronchoalveolar lavage fluid [299].

Oral administration of the IONPs is another approach mainly used for MRI of gastrointestinal (GI) tract. The gastric acids and enzymes which can degrade the IONPs rapidly are the major biological barriers for their GI delivery [28]. IONPs after successive entry into TME amalgamate with tumor-associated macrophages (TAMs), (constituents of immune system). TAMs take part in malignancy formation by inhibiting the immune system, enhancing malignancy cell growth, survival, and migration. Extracellular signals may cause phenotypic modifications in macrophages, recognized as polarization [309], dividing macrophages into two subtypes: type 1 macrophages (M1) and type 2 macrophages (M2). M1 macrophages orchestrate pro-inflammatory retaliation, identifying carcinoma cells and instigating immune feedbacks, whereas, M2 macrophages establish anti-inflammatory results, inducing growth and multiplication of neoplasm. They are the dominant population within TME and targeting these cells has displayed highly improved outcomes. [310,311].

Phagocytosis of NPs can influence TAM polarization because these particles are recognized as foreign bodies. IONPs demonstrated a potent effect on TAM polarization due to iron transporter-related protein expression [311,312,313]. Kodali et al. [314] reported 1029 changes in gene expression of lung macrophages using IONPs while silica NPs with only 67 gene. It also reduced IL-10 secretion and maximized TNF-α secretion in macrophages more than silica NPs treatments. This phenomenon is caused by the metallic core of IONPs rather than the surface coating [314].

In clinical studies, results have indicated that Fe agglomeration in tumor tissues can induce M1 polarization. A study by Reichel et al. [296] on non-small cell lung carcinoma patients revealed Fe cumulated in cells due to hemolysis caused positive correlation to CD68 expression on TAMs and had negative effect on malignancy size. The TAMs displayed increase in inducible nitric oxide synthase (iNOS) and CD86 expression, decrease in CD206 expression as well as rise in level of IL-6 secretion and also reduction in IL-10 secretion, all of these are the features of M1 macrophages. It seems that IONP accumulation near tumors caused a reduction in tumor size. This hypothesis was tested in mouse models and all the results mentioned above were reported [296].

Taken together, all those biological interactions suggest that developing experimental investigations are necessary to study systemic and local effects of the NPs on biomolecules, immune cells, and other biological components of the body.

## 7. Magnetic Nanoparticles in Clinical Applications

A substantial number of SMNPs have been developed over the last decades [243,255,277,287,315]. However, no IONPs, passively or actively targeted malignancy cells, have yet been clinically approved for therapeutic agent delivery in treatment of carcinoma [67]. In fact, the vast number of MNPs have been approved for use in the clinic as diagnostic and imaging agents such as SIONP ferumoxytol which is in phase IV clinical trials as an MRI contrast media for the detection of lymph node metastasis. The list of MNPs under clinical trial/withdrawn from the market for cancer theranostic are collected in Table 12. Although the US FDA approved IONPs as contrast agents in MRI, most have been discontinued. This is because radiologists are not fully experienced to interpret the T2 contrast signals provided by MNPs. The only FDA-approved IONP that has not been discontinued and is the most clinically investigated, as an MRI contrast media and applicable for treatment of iron deficiency in adults with chronic kidney disease in June 2009 is ferumoxytol with tradename Feraheme^®^ in the US and Rienso^®^ in Europe [316].

Clinical applications of MNPs for cancer HT have been limited by the need for the precise placement of a large AMF within the human body. Generally, FDA-approved formulations have been evaluated and optimized over the years. Simple formulation of these NPs is the most critical requirement for utilization in clinic. However, most smart IONPs have complex structures and formulations which is a major drawback for industrial production. The notable failure of using IONPs in clinical applications is the existence of various challenges that have led to insufficient efficacy and reduced interest for medical and commercial use [273]. In the following, we will briefly discuss some of the notable challenges facing for the translation of SMNPs from production on the bench toward application in patient treatment.

### 7.1. Clinical Challenges

The clinically approved nano-formulations for carcinoma treatment include Doxil (PEGylated liposomal encapsulating Dox, 1995), DaunoXome (liposome-encapsulated daunorubicin, 1996), DepoCyt (liposomal Cytarabine), Myocet (non-pegylated liposomal doxorubicin citrate, 2000), Abraxane (albumin-bound paclitaxel, 2013) and Genexol-PM (paclitaxel-loaded polymeric micelles, 2013) [321]. The aforementioned formulations improve efficacies and reduce adverse effects as compared to free drugs. To the best of our knowledge, no stimuli-responsive MNPs have been approved for carcinoma therapy. Some major obstacles for clinical translation of SMNPs for cancer therapy are mentioned here.

As previously stated, the injected smart nano-carriers face a series of complex biological constraints from the site of injection until they reach the final target destination. These include rapid elimination, escaping from endosomal and lysosomal compartments, cellular internalization, and drug efflux pumps which hinder the assembly of nano-formulas at target site and diminish their therapeutic effects [322,323]. Although on one hand the modification of NPs might be a good solution, on the other hand adding extra synthesizing steps can create further complexities and rise the production cost. It should be noted that a positive cost–benefit balance is necessary for sustainability of the launched products in the market [324,325]. IONPs used in preclinical research are almost prepared in small scales and their large-scale synthesis may not generate same quality particles. Furthermore, the complex structural design of SMNPs make the scale up for industrial production difficult. It is essential to develop low-energy-input methods for industrial-scale production of SMNPs with simple and high reproducible formulations.

Another major challenge preventing the translation of IONPs into clinic is their safety for humans. Although IONPs were approved and practiced in clinics, e.g., as iron replacements and contrast media [295,326], various studies found that parameters such as composition, size, surface, properties, dose, and route of administration can influence their safety. Several groups reported that most IONPs were not discharged from body, accumulated in vital organs, e.g., spleen and liver and led to toxicity [324]. Furthermore, the excessive release of free Fe from Fe_3_O_4_ NPs can facilitate the generation of ROS in cells and thus induce oxidative stress and disrupt liver mitochondrial function [295,324,327]. Moreover, MNPs with different coatings are toxic to brain cells and may cause neurodegenerative diseases such as Alzheimer’s and Parkinson’s disease [328]. Therefore, further studies on the long-term toxicity of SMNPs in the human body need to be conducted prior to their full clinical utilization. Another problem when using SMNPs as DDS is that deep organs within the body cannot be easily targeted by external magnets due to the absence of an effective MF gradient. To overcome this problem, the preparation of MNPs with high magnetic moments or the use of superconducting magnets such as SmBaCuO with the ability to produce strong magnetic gradients is necessary [328]. The dissimilarities between animal and human models create another pitfall in utilizing SMNPs for carcinoma treatment. Since an animal model, e.g., a mouse, cannot fully reflect the pathological conditions that exist in humans, and the nature of malignancy differs from person to person. Moreover, the lack of specific regulatory guidelines is final challenge toward commercialization of smart nano-carriers [329,330].

Last but not least, industrial scale-up, validation, reproducibility, and controllability of physicochemical properties of SMNPs remain a huge barricade hindering their clinical translation and public availability.

### 7.2. Future Perspectives

It is clear that the SMNPs have demonstrated potentials in both diagnosis and treatment of neoplasm. Although extensive research accompanied by development of advanced characterization techniques and instruments has been carried out on SMNPs during the past decades, there are still major problems related to the preparation of safe and effective smart IONPs for applications in the pharmaceutical market. Thus, the technology behind SMNPs needs further studies in order to achieve the substantial milestone in personalizing nano-medicines.

The greatest dilemma is testing on animal models which remains the fundamental key in examining the hypothesis and analyzing the safety profile of SMNPs. Hence, the way forward is to engineer research approaches and modalities which are physiologically suitable to mimic the complicated human physiology and avoid the requirement for animals, as well as overcoming the issue of disparity between human and animal species.

IONPs demonstrated a reliable outcome in nano-platforms. Study of safety profile of approved IONPs such as ferumoxytol showed they hold substantial potential for future clinical use. The incorporation of diagnosis tools, e.g., MRI, and treatment moieties, e.g., chemo-agents, into a solo platform presents a holistic opportunity for an efficient management of malignancies. Therefore, in near future it can be expected that with development of material, pharmaceutical and biomedical sciences and the combination of nano-engineering and smart chemistry, researchers will be able to design suitable stimuli-responsive SMNPs with abilities to perform as single/multi modal theranostics tool in a single platform.

## 8. Conclusions

In the present review, an attempt was made to provide a comprehensive review of SMNPs usages for malignancy detection and effective treatment, including TDD, HT, and MRI agents. The aim was also to lessen the complications of systemic chemo-agents and controlling the therapeutic efficacy of agents in tumorous tissue.

Although SMNPs are showing to be efficient in the diagnosis and treatment of carcinoma in laboratories, there are still many challenges ahead for these NPs in relation to the translation from bench to bed. Importantly, the clinical success of the SMNPs depends upon their ability to bypass chemical and biological barriers including toxicity, biodistribution, pharmacokinetics, pharmacodynamics, as well as their industrial scale-up and reproducibility for a reliable large scale production. Hence, in the future, more extensive studies are required to address the aforementioned challenges for the development of effective and practical SMNPs in cancer theranostics.

## Figures and Tables

**Table 1:** Common stimuli-responsive functional groups.

pH		Redox	Temperature	Light
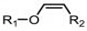 Vinyl ester	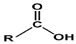 Carboxylic acid	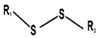 Disulphide	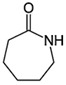 Caprolactam	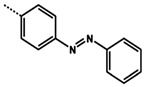 Azobenzene
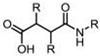 Amide	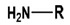 Primary amine	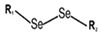 Diselenide	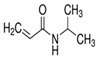 N-isopropyl acrylamide	
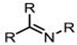 Imine	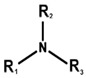 Tertiary amine			
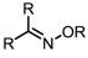 Oxime	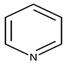 Pyridine			
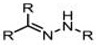 Hydrazone	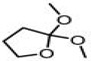 Orthoester			
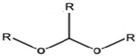 Acetal				

**Table 2:** Characteristics of nano-carriers based on Fe_3_O_4_/PIMF.

Fe_3_O_4_/PIMF		Fe_3_O_4_/PIMF-CUR			
Size (nm) (FE-SEM)	Saturation Magnetization (emu/g)	Cell Viability (%)		IC50 (mg/mL)	
		MCF-7 Cell Line	HeLa Cell Line	MCF-7 Cell Line	HeLa Cell Line
20–30	45	44	32	0.23	0.15

PIMF: Polyethylenimine-graft-poly (maleic anhydride-alt-1-octadecene)-folic acid; CUR: Curcumin.

**Table 3:** The important characteristics of Fe_3_O_4_/MPSNPs in DOX delivery system and MRI.

Fe_3_O_4_/MPSNPs			Peptide-Fe_3_O_4_/MPSNPs/DOX		
Size (nm)		Transverse Relaxivity (r_2_) (mM^−1^ s^−1^)	Loading Efficiency of Drug (%)	Drug Release (%)	
TEM	DLS			Without MMP-2 Enzyme	With MMP-2 Enzyme
114	600	135.6	12.2	15.7	70

MPSNPs: Mesoporous silica nanoparticles, Dynamic light scattering (DLS), Transmission electron microscopy (TEM), matric metalloproteinase-2-(MMP-2).

**Table 4:** The morphology, magnetic, loading, and release properties and IC50 values of Fe_3_O_4_/CM-CS and Fe_3_O_4_/β-CD.

Samples	Size (nm)		Ms (emu/g)	EEPG (%)	PG Release over 1 h (%)		IC50 (μg/mL)	
	TEM	DLS			No-Enzyme	With-Enzyme	MCF-7/GFP Cell Line	HepG2 Cell Line
Fe_3_O_4_/CMCS	9.8	38.1	65.01	91.78	6.95	44.61	0.8544	1.05
Fe_3_O_4_/β-CD	14.2	121.1	37.48	80.93	3.59	58.24	2.61	1.79

Carboxymethyl chitosan (CMCS), β-cyclodextrin (β-CD), Saturation magnetization (Ms), Encapsulation efficiency of prodigiosin (EEPG), The half-maximal inhibitory concentration (IC50).

**Table 5:** The important characteristics of M MPSNPs and M MPS/MBBs for DNA delivery.

Samples	Size (nm) DLS	Zeta Potential (mV)	Gene Transfection Rate (%)
M MPSNPs	82	−17.65	7
M MPS/MBBs	1120	31.47	14.87

**Table 6:** The pre-clinical studies of Thermo-sensitive MNPs in MHT therapy for theranostic purposes.

Formula	Shell	Application	Status	Procedure	Results	Ref
Zn_0.1_Fe_0.9_Fe_2_O_4_-Ge-Ag	Ge/Ag	-Contrast agent in MM-USI -Heat mediator in MHT	Pre-clinical MHT in phantom	-Synthesizing Zn-doped MNPs by co-precipitation (Zn_0.1_Fe_0.9_Fe_2_O_4_) -Manufacturing Ge/Ag phantom -Coating MNPs by Ge/Ag -Tissue mimicking (elasticity and acoustic) generating phantom -Applying PCC to produce powerful MF	-Cheap, non-invasive, and no ionizing effect -MM-USI provided real-time mapping of MNPs distribution, -MM-USI demonstrated successful 2D Temp monitoring prior and during MHT (for any depth within US limitation)	[233]
Fe_3_O_4_	No surfactant/capping materials	-Heat mediator in MHT -Sonosensitizer in US	In vitro In vivo	-Preparing Fe_3_O_4_ MNPs by co-precipitation -Dispersing into saline to produce MNFs	-The MNFs and US-TS produced a major rise in the cytotoxicity response of EACCs in rats -Ineffective for deep-seated tumors	[234]
ZnMn-IONCs–PEG-PCL-SiNc	PEG-PCL	-heat mediator in MHT by AMF	In vitro In vivo	-Fabricating Zn-Mn-substituted IONPS via thermal decomposition. -Synthesizing IONCs by adding Zn-Mn-dopped IONPs and PEG-PCL in THF (Zn-MN-IONCs-PEG-PCL). -Loading SiNc onto IONCs	-Doping of Zn-Mn/Fe increased adsorption rate of IONPs and improved heat efficiency. -Encapsulation by PEG-PCL caused the MNPs to cluster inside the shell and have a higher heat-efficiency. -Over 90% apoptosis of DU145 cells by MHT. -The shrinkage and inhibition of prostate tumor following four cycles of MH treatment. -No toxicity to healthy cells. -No weight loss in DU145-bearing mice.	[236]

Gelatin (Ge), Agar (Ag), Magnetomotive-ultrasound imaging (MM-USI), ultrasound (US), Ultrasound thermometry (UST), temperature (Temp), magnetic hyperthermia (MHT), Pancake coil (PCC), Ehrlich ascites carcinoma cells (EACCs), Magnetic nanofluids (MNFs), Ultrasound thermometry strain imaging (US-TS), Methoxy poly(ethylene glycol)-b-poly(ε-caprolactone) (PEG-PCL), Tetrahydrofuran (THF), Silicon naphthalocyanine (SiNc).

**Table 7:** The studies of application of MNPs in MHT therapy and CDR for theranostic purposes.

Formula	Targeting Agent	Drug	Shell	Target	Release Mechanism	Application	Status	Procedure	Result	Ref
NG-MNS-DOX	DOX	DOX	NIPAAm	MDA-MB-231 cells	TR	Chemotherapy and MHT by RF field	In vitro	-Preparing NG (carboxylate functionalization) by dissolving NIPAAm with ACA + MBAA (cross linker) + APS(catalyst) + SBS -Coating Fe_3_O_4_ by OlA and OlAm (MNS) -Covalently decorating NGs to MNS -Loading DOX on NG-MNS	-High stability (zerta potential = −28 mV) -High thermal stability -Passive targeting -Homogeneous heating and CDR due to the mono-dispersity in size/shape and uniform coating -Lower IC50 and enhanced therapeutic efficacy compared to free DOX	[137]
FMT-ML-DOX	DOX	DOX	lipid	4T1 breast cancer	TR	chemotherapy and MHT	In vitro	-Fabricating ML to enclose FMT via lipid hydration, using DPPC + Chol+ PEG-2000-DSPE in chloroform and methanol -Preparing FMT in (NH_4_)_2_SO_4_, via hydrating lipid film then collected by PESM -Encapsulating DOX into ML-FMT via ASGP, then dialysis in PBS	-TR CDR profile compared to single utilization of MHT/chemotherapy. -Higher payload (DOX) release -FMT-ML-DOX uniformly distributed	[238]
DOX- MTX-MNPs-DPPC, Chol, SA, DSPE-MPEG2000, DSPE-PEG2000-	MTX	DOX	DSPE-PEG2000-NH_2_	HeLa cells	TR	Dual imaging Dual targeting-light/MHT CDR via AMF + NIR-laser.	In vivo and In vitro	-Activating carboxylate group of MTX by NHS + DCC + DMSO -Followed by introducing DSPE-PEG2000-NH_2_ to it (DSPE-PEG2000-NH_2_-MTX) -Preparing OlA-coated-MNPs -Synthesizing MTX-MTRLs by adding DPPC, Chol, SA, DSPE-MPEG2000, DSPE-PEG2000-MTX and MNPs -Encapsulating DOX onto MTX-MTRLs	-Combination of AMF and NIR laser highly improved the DOX uptake, CDR and TDD into HeLa cells -MTX increased cytotoxicity to malignancy cells while the side effect to normal cells decreased	[239]
Fe_3_O_4_-Ge-HA-EDA-GO-DOX/PTX	HA	DOX/PTX	Ge	MDA-MB-231 and BT-474	TR	-TDD and MHT	In vitro	-Preparing GO via the Hummer reaction and sonication -Aminating the HA by EDA followed by addition of NHS to activate the COOH group (HA-EDA) -Functionalizing GO by HA-EDA -Fabricating Fe_3_O_4_ MNPs via co-precipitation -Coating Fe_3_O_4_ by Ge -Adding Fe_3_O_4_—Ge to GO-HA-EDA to generate MN-composite -Loading of DOX/PTX onto Fe_3_O_4_—Ge—GO-HA-EDA	-DOX loaded MN-had higher efficacy compared to PTX. -Higher performance of HA functionalized MN-composites in destroying MDA-MB-231 with overexpressed CD44, not BT-474 cells since they lack CD44 -Incorporation of DOX and GO-HA MN-composite with MHT exhibited high killing efficacy	[240]
NIPAAm-PCL-NIPAA-MNP-OlA -PTX	PTX	PTX	NIPAM-PCL-NIPAM	MCF-7	TR	-TDD, HT and chemotherapy	In vitro	-Fabricating of MNPs via co-precipitation -Adding OlA to MNPs (MNP@OlA) -Preparing PCL-diol polymer (using ε-CL + Sn(Oct)_2_ -Synthesizing triblock polymer by adding PCL-diol to THF + ALBN + NIPAM (PNIPAAm-PCL-PNIPAAm) (FRP) -Forming MNP micelles via solvent evaporation adding, PNIPAAm-PCL-PNIPAAm + THF + MNP@OlA -Loading PTX onto MNP-micelles	-Successful formation of PCL-diol and triblock PNIPAAm-PCL-PNIPAM -Biocompatible MNPs -PTX loaded MNPs were toxic to MCF-7 cell line. -Higher PTX release 89.3 ± 2.7% at Temp (42 °C) -Increasing Temp, decreased hydrodynamic diameter (size = 30–40 nm was thermal dependent)	[242]
SIONPPs-PEG-PBA-PEG-FA-TMZ	FA	TMZ	PEG-PBA-PEG	C6 GM cells	TR	-As heat mediator of AMF	In vivo and in vitro	-Fabricating of SIONPs via co-precipitation -Preparing PEG-PBA-PEG via poly-condensation and coupled with FA. -Loading TMZ onto SIONPs-PEG-PBA-PEG-FA via DESE	-Slight reduction in Ms value, due to FA and TMZ -Superparamagnetism with no magnetic hysteresis -Homo-compatible -High therapeutic efficiency, eradicating deeply placed tumors, e.g., brain GM-SIONPs-PEG-PBA-PEG-FA-TMZ remained inactive at 37°, activated when temp raised to 43° by AMF, resulting in an increase in the rate of drug release -Removal of AMF resulted in reduction in drug concentration, confirming the TR MNP facilitated the CDR -Improved thermal stability for hydrophilic phase	[243]

Ammonium persulfate (APS), Oleic acid (OlA), Poly N-isopropylacrylamide (PNIPAAm), N-isopropylacrylamide (NIPAM), Nanogel (NG), Acrylic acid (ACA), N,N′-methylenebisacrylamide (MBAA), Oleylamine (OlAm), ammonium sulfate (NH_4_)_2_SO_4_, Polyethersulfone membrane (PESM), Magnetic nano-system (MNS), Radio frequency (RF) field, Magnetic liposeme (ML), Ferumoxytol (FMT), Dipalmitoylphosphatidylcholine (DPPC), Poly-ethylene-glycol-2000-distearoyl-phosphatidyl-ethanolamine (PEG-2000-DSPE), Temozolomide (TMZ), Folic acid (FA), Glioblastoma (GM), Alternative magnetic field (AMF), Saturation magnetization (Ms), Poly (ethylene glycol)−poly (butylene adipate)-poly (ethylene glycol) (PEG-PBA-PEG), Double emulsion solvent evaporation (DESE), Iron oxide nanoparticles (IONPs), Drug Delivery (DD), Hyaluronic Acid (HA), Ethylene diamine (EDA), Polycaprolactone diol (PCL-diol), Cholesterol (Chol), 1,2-distearoyl-sn-glycero-3-phosphoethanolamine-N-[amino(polyethylene-glycol)-2000] (DSPE-PEG 2000), Dynamic light scattering (DLS), Polydispersity index (PDI), Magnetic Thermo-responsive liposome (MTRL), Stannous-2-ethylhexanoate (Sn(Oct)_2_), Azobisisobutyronitrile (AIBN), Muramyl dipeptide (MDP), Ammonium sulfate gradient protocol (ASGP), Dimethyl sulfoxide (DMSO).

**Table 8:** Examples of application of SIONPs in Drug Delivery and imaging in single platform.

Formula	Targeting Agent	Drug	Shell	Target	Release Mechanism	Status	Procedure	Result	Ref
Dex-SIONPs-Manevist-fluorescein-1,2-DPPC, DSPC, DSPE-PEG-2000	Fluorescein	Fluorescein and	1,2-DPPC, DSPC, DSPE-PEG-2000	Non-specific	TR	In vivo In vitro	-Producing M-HS via mixing commercially purchased Dex-IONPs with DTPA and fluorescein in PBS -Fabricating LMNPs using 1,2-DPPC, DSPC, DSPE-PEG-2000 -Hydrating LMNPs by M-HS -Exposing the LMNPs to AMF to release the drug model.	-High stability and dispersity, the PDI of LMNP before AMF = 0.134 and d = 231 nm, after AMF PDI = 0.1 and d = 223 nm at physiological pH = 7.4, confirming LMNPs stayed within liposome during and after the procedure. -Targeting malignancy via EPR -PEG enabled LMNPs to have prolong BCT -Applying AMF to LMNPs, fluorescein remained intact. even at 80° C -No drug release without AMF -A 100% drug release at 37 °C—37 °C by AMF -R1 showed CDR, concurrent release of DTPA and Fe -High-resolution/contrast and imaging quality. -Potential in MRI cancer theranostics	[164]
SA-MPDA-SPIO-DOX-Fe^3+^	SA	DOX	SA-PEG-NH_2_	HepG2 and Bel-7402 cells	pH and TR	In vivo In vitro	-Generating OlA-stabilized SIONPPs via thermal-decomposition. -Co-assembling MPDA and SIONPs by soft-templating (MPDA-SIONPs) -Synthesizing (SA-PEG-NH_2_) -Altering surface of MPDA-SIONPPs by SA-PEG-NH_2_ (MPDA-SIONPPs-SAPEG) -Chelating Fe^3+^ by MPDA-SIONPPs and MPDA-SIONPPs-SAPEG using FeCl_3_ -Encapsulating DOX onto MPDA-SIONPPs-SAPEG-FE^3+^	-Biocompatible and dispersible in water -Successful modification of MNPs with SA in SA-PEG-MPDA-SPIO-Fe^3+^ -Highly precise T1/T2 MRI effect -An increased therapeutic efficacy due to active interaction of SA and E-selectin in vitro -Exceptional PTC ability and photostability.	[180]
Fe_3_O_4_-PDMAEMA/ PNIPAAm/MTX	MTX	MTX	MS PNIPAAm-PDMAEMA	A549 lung cancer cell	pHR and TR	In vitro	-Generating multi-modal MNPs via co-precipitation -Modifying MNPs by TMSMA to supply vinyl-link on the MNPs surface (MNPs + acetic acid + ethanol + TMSMA) -Fabricating MPSNPs via Stober method ((NH_4_OH + anhydrous ethanol + TEOS), precipitating with n-hexane -Producing CIL using DMAEMA and CPTMS -Preparing CIL-MPSNPs using (CIL monomer + DMSO + MPSNPs) -Conjugating MNPs-TMSMA, CIL-MPSNPs and NIPAAm to produce dual sensitive MNSs -Loading MTX (prepared in PBS) onto the MNSs (MNS-MTX)	-Increased antitumor activity of MNPs (MNS-MTX) and CDR, due to entering the cell via R-ME -Applicable as T2 MRI contrast agent in vitro -Polymerized MPNCs sustained their magnetic characteristics -MNS-MTX demonstrated potential in vivo application because of passive targeting (EPR effect) and TDD via MF -The MNPs aggregated in malignant tissue -Dose-dependent anti-neoplasm efficacy in A548 cells -Reduced adverse effects. -Biocompatible and only minor cytotoxicity due to high dose.	[246]
Fe_3_O_4_/MOF/CD/DOX/AS1411 Apt	AS1411 Apt	DOX	UiO-66-NH_2_	MDA-MB-231 HBC cells	pHR	In vitro	-Ultra sonicating Fe_3_O_4_ in DMF and mixing with UiO-66-NH_2_ in NH_2_-BDC to generate Fe_3_O_4_@MOF core-shell MNPs -Loading DOX (DOX prepared in PBS, pH 8, 24 h) onto Fe_3_O_4_@MOF (Fe_3_O_4_@MOF-DOX) -Activating CDs by EDC and NHS (preparation in dark to activate the acid groups) for covalent conjugation with Fe_3_O_4_@MOF-DOX (Fe_3_O_4_@MOF-DOX-CDs) -Dissolving AS1411 Apt in DIW and US to conjugate with Fe_3_O_4_@MOF-DOX-CDs (Fe_3_O_4_-MOF@DOX-CDs-AS1411 Apt)	-High stability (up to 6 days in vitro) -Safe for HUVEC -Improved drug loading efficiency, tumor uptake and pHR drug release -TDD to nucleus of the triple-negative MDA-MB-231 HBC via overexpressed receptors, nucleolin -Effective anti-proliferation and promoting apoptosis in MDA-MB-231 HBC cells (77% cell apoptosis after 24 h) -Enhanced cancer cell targeting and binding affinity -Ability as FL bio-imaging	[247]
Fe_3_O_4_/CDNSs/FA/CUR	FA	CUR	β-CD	M109 cells	pHR	In vitro	-Polymerizing βCD and EPI as cross-linking agent to produce CDNS using anhydrous (βCD + DMSO + Et_3_N. EPI) -Synthesizing Fe_3_O_4_ NPs@CDNS via dispersing Fe_3_O_4_ NPs in (DMSO + βCD + Et_3_N + EPI) -Adding carboxyl group onto Fe_3_O_4_ NPs/CDNS using back titration method, dispersing Fe_3_O_4_ NPs-CDNS in (NaOH + HCl) -Fabricating FA-hydrazide (FA-NH-NH_2_) by dissolving FA in (DMSO + NHS + EDC) then hydrazine hydrate and converting into hydrochloride salt (using HCl) and precipitating by diethyl ether/acetonitrile and finally washing with ethanol -Conjugating FA-hydrazide with Fe_3_O_4_ NPs/CDNS, stirring Fe_3_O_4_/CDNS + DMSO + EDC and adding FA-hydrazide (Fe_3_O_4_/CDNS-FA NP) -Loading CUR into Fe_3_O_4_/CDNS-FA NP using (PBS + CUR in acetone)	-Fe_3_O_4_/CDNSs-FA-CUR was highly toxic to FRP M109 cells compared to FRN MCF 10A cells -Enhanced MRI (T2 negative signal) -Satisfying drug loading capacity -Acceptable agent release profile and TDD -Successful performance in theranostic platform -More in vivo studies on the potency of the nano-sponge to shrink the tumor will be required.	[248]
Fe_3_O_4_/Dex/ETB	ETB	ETB	Dex	CL1-5-F4 cancer cells	pHR	In vivo In vitro	-Preparing ultra-small Fe_3_O_4_ MNPs via co-precipitation -Coating Fe_3_O_4_ with Dex (Fe-D MNPs) -Crosslinking Dex coating with Epichlorohydrin/NaOH -Adding primary amino groups on the surface of IONPs (FeDN MNPs) -Treating FeDN MNPs with DMSO in buffer (pH:8.5, 0.1 M NaHCO_3,_ dialysis with DIW for 3 days), adding MPTSA and NaCl generating FeDC MNPs -Conjugating FeDNC MNPs with ETB (FeDNC-E MNPs)	-Higher cellular uptake of ETB and intracellular drug delivery. -High re-ignition ability to kill cells with overexpressed EGFR receptors and leaving EGFR-negative cells intact -FeDNC-E MNPs suppressed EGFR–ERK–NF-κB signaling pathways -FeDNC-E MNPs inhibited, migration and metastasize of extremely intrusive CL1-5 F4 lung cancer cells in vivo xenograft. -Non-invasive real-time tracking of tumor by MRI -Decreased MRI T2 values in tumor cells compared to non-targeted cells -Promising for clinical application in targeted treatment and MRI	[249]
MMPSNP-Imi/Cis-Pt	Cis-Pt	Cis-Pt	TIP	Ovary cells	pHR	In vitro	-Synthesizing Fe_3_O_4_ MNPs via co-precipitation -Stabilizing MNPs by capping with CA (Fe_3_O_4_-CA) -Preparing MMPSNPs via sol-gel method, adding Fe_3_O_4_-CA+CTAB (pore constructing agent)+TEOS -Functionalizing MMPSNPs with Imi-groups using anhydrous toluene and TIP (MMPSNPs-Imi) -Replacing chloro-ligand with aqua-ligand via suspending Cis-Pt in DI water and AgNO_3_ to precipitate in the dark, centrifuged to remove AgCl and diluted with DIW to obtain 0.1 mg/mL -Adsorbing Cis-Pt on MMPSNPs-Imi via direct incubation (mixing at 37 °C in the dark for 24 h).	-Functionalizing MMPSNPs with Imi-groups weakened Cis-Pt interactions and resulting CDR -Replacing chloro-ligand with aqua-ligand enhanced the reactivity of Cis-Pt in aqua-solution. -Sustained and prolonged Cis-Pt release, due to the mesoporous features (e.g., pore size/opening, ratio of NPs to pore size, attraction of NPs to pore walls) prevents Cis-Pt diffusion into the pores instead adsorbed on the surface -High Cis-Pt loading capacity -With no-burst, pHR, and constant Cis-Pt release in acidic conditions resulting in growth inhibition of HEOC cancer cells -Successful apoptosis and necrosis -Enhanced T2-weighted MRI (high transverse relaxivity) -Potential in vivo application as chemo and contrast agents TDR	[250]
Fe_3_O_4_-FTY720-PFP-RGD	FTY720	FTY720	RGD	HepG2 and Huh7 cells	US-responsive	In vitro	-Synthesizing SIONPPs via organic phase decomposition -Loading drug onto SIONPs via thin film hydration pathway, dissolving 1,2-DPPC+DSPE-PEG-MAL+FTY720 in THF, adding SIONPs. Mixing with buffered saline and sonicated. Adding PFP then ultrasonicated, emulsified and dialyzed for 3 day, store at −4 °C to produce FTY720@SIONP/PFP/NBs -Mixing FTY720@SIONP/PFP/NBs + EDC + NHS then adding RGD-peptide. -Dialyzing with DIW for 3 day and keep at −4 °C to develop FTY720@SIONP/PFP/RGD-NBs	-Enhanced FTY720 release and active targeting due to LIFUS -High stability, good encapsulation, and agent loading efficiency -Low toxicity to normal fibroblast 3T3 cells -High inhibition of HepG2 and Huh7 cells -High relaxation value and T2-weighted MRI -MNBs resulted in an increase in EPR results -Induced HepG2 apoptosis via activating Caspase3, Caspase9, and p53	[253]
Fe_3_O_4_-INU-LA-PEG-FA-DOX	FA	DOX	INU-LA-PEG	Colon cancer	RR	In vivo in vitro	-Synthesizing INU-LA-PEG-FA via microwave radiation, by dispersing (INU + BNPC + (±)-α-lipoic acid (LA) in DMF), adding PEG-FA -Coating SIONPs with INU-LA-PEG-FA -Loading DOX-HCl onto SIONP@INU-LA-PEG-FA	-Improved cancer inhibition due to the presence of FA -Reduced tumor volume -Enhanced MRI in vivo, promising in locoregional chemo-treatment	[254]
Fe_3_O_4_-PDMAEMA/ PNIPAAm/MTX	MTX	MTX	MS PNIPAAm-PDMAEMA	A549 lung cancer cell	pHR andTR	In vitro	-Generating multi-modal MNPs via co-precipitation -Modifying MNPs by TMSMA to supply vinyl-link on the MNPs surface via ultrasonicating (MNPs + acetic acid + ethanol + TMSMA) -Fabricating MPSNPs via Stober method ((NH_4_OH + anhydrous ethanol + TEOS), precipitating with n-hexane -Producing CIL using DMAEMA and CPTMS -Preparing CIL-MPSNPs using (CIL monomer + DMSO + MPSNPs) -Conjugating MNPs-TMSMA, CIL-MPSNPs and NIPAAm to produce dual sensitive MNSs -Loading MTX (prepared in PBS) onto the MNSs (MNS-MTX)	-Increased antitumor activity of MNPs (MNS-MTX) and CDR, due to entering the cell via R-ME -High relaxivities in vitro, indicating the ability of MNPs to sustain their magnetic characteristics. -MNS-MTX demonstrated potential in vivo application because of passive targeting (EPR effect) and TDD via MF -The MNPs aggregated in malignant tissue -Dose-dependent anti-neoplasm efficacy in A548 cells -Reduced adverse effects. -Biocompatible and non-cytotoxic effect on A549 cell line in vitro, -Minor cytotoxicity due to high dose.	[246]
Fe_3_O_4_-GO-DOX	FA	DOX	GO	MGC-803 cells	pHR	In vitro In vivo	-Fabricating NGO by cutting GO sheet and adding AgNO_3_ (redox reaction) -Preparing Fe_3_O_4_@NGO NPs via one-pot hydrothermal pathway (FeCl_3_·6H_2_O + ethylene glycol + NaOH + NGO) -Conjugating FA with Fe_3_O_4_@NGO MNPs via coupling reaction (EDC and NHS), followed by adding Fe_3_O_4_@NGO Loading DOX onto FA-Fe_3_O_4_@nGO via precipitating with a permanent magnet	-Good targeting capability for solid tumors in vivo -Biocompatible, safe and no cytotoxicity to normal cell -Higher uptake by tumor cells and lower uptake by residual organs, e.g., liver, kidneys -Increased cancer cell targeting -Due to the nano-size and conjugation with FA, the sharpness of GO NPs reduced (cell membrane damage decreased) and had lower oxidative stress in vitro and in vivo -MRI performed after 8–24 h of administration of NPs to tumor bearing mice; -showed decrease in MRI signal in FA-Fe_3_O_4_@nGO-DOX -and decrease in MRI signal in Fe_3_O_4_@nGO-DOX, due to the EPR effect.	[255]
Fe_3_O_4_-Au-LA-CUR	GSH	LA-CUR	Au LA-CUR-GSH	U87MG cells	pHR	In vivo in vitro	-Synthesizing Fe_3_O_4_ via co-precipitation -Functionalizing of Fe_3_O_4_ by APTES (Fe_3_O_4_-NH_2_) -Fabricating Fe_3_O_4—_Au NCs via wet-chemical, using (HAuCl_4_.2H_2_O and tri-sodium citrate+NaBH_4_) -Conjugating LA onto CUR (LA-CUR), using CUR + LA + DMAP in DCM, adding EDC -Bonding GSH with Fe_3_O_4_—Au NCs via swapping its thiol group with citrate group of AU-NPs -Attaching LA-CUR onto the Fe_3_O_4_-Au NCs via ligand swap of S-S bond in LA with citrate of Au NPs (Fe_3_O_4_-Au-LiA-CUR) -Adding LA-CUR and GSH simultaneously to Fe_3_O_4_-Au NCs forming Fe_3_O_4_-Au-LA-CUR-GSH	-Enhanced cytotoxicity -Great LA-CUR loading efficiency -Efficient and CDR in vitro -Highly biocompatible due to minimum protein adsorption and good hemocompatibility -Capable of crossing BBB -Successful TDD due to GSH targeting agent -High cellular uptake by GSH receptor positive astrocyte cell -Highly promising for MRI negative contrast media for brain tumor imaging/therapy	[256]
AuNCs−Fe_3_O_4_/MDP-FITC/PFP	DCs	MDP	PEG2000-NH_2_	Y79 cells	TR	In vivo in vitro	-Activating carboxyl group of citrated Au-NCs using EDC and NHS. -Conjugating PEG2000@Fe_3_O_4_ (−NH_2_) to Au-NCs by amide bond. -Adding MDP and FITC in PBS to Au-NCs–Fe_3_O_4_ at room temp and absence of light. -Loading PFP into Au-NCs/Fe_3_O_4_/MDP-FITC	-MNPs exhibited homogeneous size, good dispersity and superparamagnetism -Potential for multi-modal application, T2-MRI, MHT, TDD and CDR. -Facile transportation of MNPs in blood stream and assembly in RB tumor via EPR effect. -Biocompatible and non-toxic to healthy cells at high Temp (43.2 °C), leading to apoptosis/necrosis of Y79 cells. -Irradiation by LIFU resulted liquid PFP changes to gas that improve LIFU efficacy and US imaging and release MDPs which bound to NOD2 receptor resulting activation of DCs to identify and eliminate RB tumor cells.	[257]

Fluorescence imaging (FL), Dimethyl formamide (DMF), pH-responsive (pHR), 1,2-dipalmitoyl-sn-glycero-3-phosphocholine (1,2-DPPC), 1,2-distearoyl-sn-glycero-3-phosphocholine (DSPC), Liposome magnetic nanoparticle (LMNPs), Sialic acid (SA), Diethylenetriaminepentaacetic acid gadolinium(III) dihydrogen salt hydrate Gd(III)-DTPA (Magnevist), Zirconium 2-amino-1,4-benzenedicarboxylate (known as UiO-66-NH_2_), Metal-organic-frameworks (MOF), Carbon Dots (CD), Magnetic-Hydrating Solution (M-HS), 1-Ethyl-3-(3-dimethylaminopropyl)carbodiimide (EDC), 2-aminoterephthalic acid (NH_2_-BDC), Phosphate buffer solution (PBS), N-hydroxysuccinimide (NHS),Redox responsive (RR), Thermo-responsive (TR), nucleolin-binding aptamer (AS1411 Apt), Human Breast Carcinoma (HBC), Human Umbilical Vein Endothelial Cells (HUVEC), Cyclodextrin nanosponges (CDNSs), Folate receptor-positive (FRP), Madison lung carcer cell line (M109), Deionized water (DIW), Mesoporous polydopamine (MPDA), Photothermal Conversion (PTC), Folate receptor-negative (FRN), Normal human mammary epithelial cell line (MCF 10A), Dextran (Dex), Erlotinib (ETB), 4-Morpholineethanesulfonic acid (MPTSA), Bis(4-nitrophenyl)carbonate (BNPC), Doxorubicin hydrochloride (DOX-HCl), 3-(trimethoxysilyl)propyl methacrylate (TMSMA), Cationic ionic liquid (CIL), (N,N-dimethylaminoethyl methacrylate) (DMAEMA), Targeted drug release (TDR), (3-chloropropyl)trimethoxysilane (CPTMS), N-isopropylacrylamide (NIPAAm), Magnetic nano-system (MNS), Receptor-mediated endocytosis (R-ME), Mesoporous silica (MS), Tetrahydrofuran (THF), Nano-Graphene Oxide (NGO), Human gastric cancer cells (MGC-803), Tetraethyl orthosilicate (TEOS), Citric acid (CA), Triethoxy-3-(2-imidazoline-1-yl)propylsilane (TIP), Hexadecyltrimethylammonium bromide (CTAB), magnetic nanoparticles (M-MPS-NPS), Human epithelial ovarian carcinoma (HEOC), Aminopropyltriethoxy silane (APTES), poly(2-ethyl-2-oxazoline) (PEtOx), Magnetic Nanobables (MNBs), Fingolimod (2-amino-2[2-(4-octylphenyl)ethyl]-1,3-propanediol, 1,2-distearoyl-sn-glycero-3-phosphoethanolamine-N-[maleimide(polyethylene glycol)] (DSPE-PEG2000-MAL), Perflenapent (PFP), low-intensity focused ultrasound (LIFUS), Uppsala 87 Malignant Glioma (U87 MG), Nanocomposite (NCs), Lipoic acid (LiA), Receptor-Positive (R-P), Fluorescein isothiocyanate (FITC), Photoacoustic (PA), Dendritic cell (DC).

**Table 9:** The morphology, magnetic, loading and cytotoxicity properties of Fe_3_O_4_/PNVCL MNPs.

Size (nm) DLS	Saturation Magnetization (emu/g)	5-FU Loading Capacity (mg/g)	The Viability of SW620 Cells (%)
423.5	7.7	400	90

**Table 10:** The main characteristics of an Fe_3_O_4_/polydopamine/hyaluronic acid nanocomposite.

Size (nm) DLS	Saturation Magnetization (emu/g)	DOX Loading Capacity (%)	DOX Release within 24 h (%)			Transverse Relaxation Rate (mM^−1^s^−1^)
			In the Absence of Any Stimulus	In the Presence of pH = 5.5, GSH	In the Presence of pH = 5.5, GSH and Laser	
120	28.5	7.13	3.6	9.96	32.7	171.76

**Table 11:** Examples of Active Targeted Drug Delivery of SIONPs for cancer treatment.

Formula	Targeting Agent	Drug	Shell	Target	Release Mechanism	Procedure	Result	Ref
Fe_3_O_4_/Au/OPSS-PEG-SVA-/PDMAEMA/MTX	MTX	MTX	OPSS-PEG-SVA/PDMAEMA	MCF7 and MDA-MB-231 BC cells	pHR	-Preparing polymer solution via NH_2_-terminating DMAEMA ((+)ly charged, pHR polymer) (NH_2_-PDMAEMA) -Fabricating Fe_3_O_4_ via co-precipitation -Developing Au-Fe_3_O_4_ MNPs via standard citrate reduction -PEGylating MNPs using OPSS-PEG-SVA (MNPs@OPSS-PEG-SVA) -Adding PDMAEMA onto MNPs@OPSS-PEG-SVA under Ar gas (MNPs@polymer) -Loading MTX onto MNPs@polymer	-Improved EPR effect -Prolonged blood circulation -Safe and CDR at physiological pH -Improved cytotoxic activity MCF7 and MDAMB231 cell lines -Potential candidate for passive and active TDD	[276]
Fe_3_O_4_/MSN/PEI/ETB	FA	ETB	MS and PEI	HeLa cells	pHR	-Synthesizing of Fe_3_O4 vi_a_ co-precipitation -Coating Fe_3_O_4_ NPs with silica to produce (Fe_3_O_4_@MSN) using TEOS and CTAB -Conjugating FA with PEI to produce PEI-FA using FA+DCC+NHS+PEI in coupling reaction -Preparing Fe_3_O_4_@ MSN/PEI-FA, using Fe_3_O_4_@MSN+modified PEI-FA+acetic acid -Loading ETB onto Fe_3_O_4_@ MSN/PEI-FA via dissolving ETB in DMSO	-Fe_3_O_4_@MSN/PEI-FA-ETB demonstrated higher cytotoxicity effect on HeLa cells compared to Fe_3_O_4_@MSN-ETB/PEI (due to lacking TA) -Non-toxic effect -TDD and CDR with minimum side effects -Promising TDD tool for in vivo applications	[277]
Fe_3_O_4_/CS/SH/FA/Co 6	FA	Co 6	CS	HeLa cells	RR	-Thiolating Fe_3_O_4_ and CS separately. (Fe_3_O_4_-SH) and (CS-SH) -Coupling CS-SH with (1) FA (FA-CS-SH) (2) RFP (RFP-CS-SH) -Followed by mixing (1) and (2) in HSO, dispersing Co 6 and ultrasonicating to generate Fe_3_O_4_/CS/SH/FA/Co 6	-Higher internalization into HeLa cells compared to the non-targeted MNPs	[283]
Fe_3_O_4_/PLH−PEG −LiA/FA-PEG/DOX	FA	DOX	Si	MCF-7, MDA-MB-231, MCF-10A cells	pHR and RR	-Preparing MNPs via hydrothermal reaction -Reacting MNPs with TEOS to produce Fe_3_O_4_@SiO_2_ -Preparing MPS coating (using C18TMS), to produce Fe_3_O_4_@SiO_2_@MSiO_2_ -Loading Dox onto Fe_3_O_4_@SiO_2_@MSiO_2_ -Preparing polymer (PLH−PEG− NH_2_) using coupling reaction (EDC and NHS reagents) -Connecting LiA to PLH−PEG−NH_2_ using DCC coupling reagent to produce PLH−PEG−LiA polymer -Synthesizing FA−PEG−NH_2_ via coupling reaction	-Elipsoidal-shaped MNPsenhanced uptake and cell morbidity compared to the non-targeted NPs against BC cell -Substantial increase in DOX release in the presence of 10 mM GSH at pH 5.5 (97.1%) as compared to pH 7.4 (31.7%) within 24 h	[284]
Fe_3_O_4_/SiO_2_/MUC1 Apt/DOX	MUC1 Apt	DOX	SiO_2_	MCF-7 and MDA-MB-231 BC cells	pHR	-Preparing SIONP using thermal decomposition -Developing SIONPs@SiO_2_-NH_2_ using TEOS and APTEs in the presence of CTAB -Fabricating SIONPs@SiO_2_-COOH via reacting SIONPs@SiO_2_-NH_2_ with succinic anhydrate in DMF -Anchoring DOX onto SIONP-SiO_2_-COOH -Decorating SIONP@SiO_2_-COOH/DOX by MUC1 Apt -Labelling SIONP@SiO_2_/DOX/MUC1 Apt with fluorophore using PBS and FITC	-Remarkably high drug release in acidic TME -Potential multi-modal candidate used for diagnosis and treatment of MUC1 overexpressed malignant cells. -Higher toxicity and internalization by MUC1 expressing MCF-7 cells	[287]
Fe_3_O_4_/PEI/HPhBA/DOX	PhBA	DOX	PEI	U-87 MG	pHR	-Functionalizing Fe_3_O_4_ by NH_2_ groups (Fe_3_O_4_-NH_2_) -Preparing NH_2_ groups of Fe_3_O_4_-NH_2_ by PEI -Reacting with PhBA to fabricate HPhBA-MNPs	-Increased therapeutic effect on U-87 MG malignant glioma cells. -Improved cellular uptake and CDR	[288]
Fe_3_O_4_/GO multi/Lf DOX	LF	DOX	-GO	C6 glioma cells	pHR	-Encapsulating Fe_3_O_4_ by GO (GO/Fe_3_O_4_) -Functionalizing by LF via click chemistry for TDD -Loading DOX onto MNPs	-Intracellular delivery efficiency -Increased cytotoxicity against C6 glioma cells	[289]
Fe_3_O_4_/TMSMA/QDMAMEA/Aly-Imz/CD/MTX	MTX	MTX	β-CD	Saos-2 bone cancer cells	pHR	-Grafting β-CD onto Aly-Imz (via reflux-coprecipitation) -Preparing CMNPs via FRPR (using β-CD/Aly-Imz/QDMAME/TMSMA in DMSO under N_2_), followed by co-precipitation -Loading with MTX for TDD to Saos-2 cells	-Successful pH-responsive characteristics -Increased cytotoxicity, -Enhanced cellular uptake in Saos-2 cells -No major cytotoxicity effects on HRBCs.	[290]
Fe_3_O_4_/CS/PNIPAAm-Co-IA/MTX/ETB	MTX	ETB	CS-NIPAAm-IA	OVCAR-3 cells	pHR and TR	-Modifying CS with SDS and MaA, (generating polymerizable organo-soluble precursor) -Grafting NIPAAm and IA (TR and pHR monomers) onto CS via FRPR (co-polymerization) -synthesizing MNPs via co-precipitation -Developing MNPs@CSC -Activating MTX via coupling reaction (using EDC and NHS reagents) -Conjugating MTX with MNPs@CSC (producing MNPs@CSC-MTX) -Loading ETB onto MNPs@CSC-MTX (producing MNPs@CSC-MTX-ETB)	-spherical-shaped MNPs -High loading efficiency for ETB. -Increased cytotoxicity and -Higher cellular uptake of MTX, by FR-positive cells -TDD and improved drug release -Potential theranostic nano-system for the targeted imaging/treatment of solid tumors, e.g., ovarian	[291]
O-CMCS-Fe_3_O_4_-PEM	PEM	PEM	O-CMCS	A549-luc-C8 and CRL5807 cell	pHR	-Improving CS solubility by O-CM (O-CMCS) -Encapsulating O-CMCS by Fe_3_O_4_ to produce MNPs (O-CMCS MNPs) -Loading PEM onto O-CMCS MNPs	-Highly promising therapy for NSCL carcinoma	[292]
Fe_3_O_4_/MUC-1/PEG//DOX	MUC-1 Apt	DOX	PEG	MDA-MB-231 and MCF-7 cells	pHR	-PEGlayting SIONPs -Decorating by MUC1-Apt	-Higher uptake as compared to non-specific targeted NPs, -Increased death rate in MCF-7 cells	[293]

Folic Acid (FA), Targeted drug delivery (TDD), Hyperthermia (HT), Polyethylene glycol (PEG), Cationic magnetic nanoparticles (CMNP), Allyl imidazole (Aly-Imz), Free radical polymerization reaction (FRPR), Dimethyl sulfoxide (DMSO), Human red blood cells (HRBC), Orthopyridyl disulfide-poly(ethylene glycol)-succinimidyl valerate (OPSS-PEG-SVA), poly (butylene adipate) PBA, Dimethyl formamide (DMF), Temozolomide (TMZ), 3-(Trimethoxysilyl) propyl methacrylate (TMSMA), Mesoporous nano-cage (MPNCs), Quaternized ammonium alkyl halide N,N-dimethylaminoethyl methacrylate (QDMAMEA), Phenylboronic acid (PhBA), hyperbranched phenylboronic acid (HPhBA), Targeting Agent (TA), β-Cyclodextrin (β-CD), Positively ((+)ly), Methotrexate (MTX), Oleic acid (OlA), 1,2-Distearoyl-sn-glycero-3-phosphoethanolamine-N-[maleimide(polyethylene glycol)] (DSPE-PEG-MAL), Lactoferrin (LF), 1,2-dipalmitoyl-sn-glycero-3-phosphocholine (1,2-DPPC), Perflenapent (PFP), Fingolimod (FTY720), Poly(N-isopropylacrylamide) (PNIPAAm), Itaconic acid (IA), Ovarian cancer cells (OCC), Sodium dodecyl sulfate (SDS), Maleic acid anhydride (MaA), Chitosan co-polymer (CSC), MUC-1 aptamer (MUC-1 Apt), Tetra-ethyl ortho-silicate (TEOS), Phosphate-buffered saline (PBS), Fluorescein isothiocyanate (FITC) octadecyltrimethoxysilane (C18TMS), Poly-L-histidine (PLH), Lipoic acid (LiA), 3-aminopropyltriethoxysilane (APTES), Cetyltrimethylammonium bromide (CTAB), Red fluorescent probes (RFP), Hydroxy silicon oil (HSO), Coumarin 6 (Co 6), Redox Responsive (RR), Pemetrexed (PMX), O-Carboxymethyl chitosan (O-CMCS), Non-small-cell-lung (NSCL).

**Table 12:** Magnetic iron oxide nanoparticles approved or in clinical trials.

Name	Coating	Application	Status	Ref
Ferumoxytol (Feraheme^®^)	Carboxymethyl dextran	Head and Neck imaging	Clinical trial (Early Phase I)	[317]
Magnablate I	Not available	MHT on prostate cancer	Clinical trial (Early Phase I)	
Ferumoxytol/Gadobutrol	carbohydrate	Urinary bladder imaging	Clinical trial (Early Phase I)	
Ferumoxytol (Feraheme^TM^)	Carboxymethyl dextran	Imaging for lymph node staging in esophageal cancer	Clinical trial (Phase I)/discontinued	
Ferumoxytol (Feraheme^®^)	Carboxymethyl dextran	Imaging of lymph node involvement in prostate cancer	Clinical trial (Phase I)	
Ferumoxytol (Feraheme^®^)	Carboxymethyl dextran	Imaging of lymph nodes in patients with primary prostate or breast cancer	Clinical trials (observational study)	
Ferumoxytol (Feraheme^®^)	Carboxymethyl dextran	Imaging of lymph node metastases in prostate, bladder, and kidney cancers	Clinical trial (phase II)	
Ferumoxytol (Feraheme^®^)	Carboxymethyl dextran	Brain tumor imaging	Clinical trial (phase II)	
Ferumoxytol/MM-398	Carboxymethyl dextran	Imaging of solid tumors	Clinical trial (phase I)	
Ferumoxytol (Feraheme^®^, Ferumoxytol non-stoichiometric magnetite)/Gadolinium	Carboxymethyl dextran	Imaging of primary or metastatic brain tumors	Clinical trial (phase II)	
Ferumoxytol (Feraheme^®^, Ferumoxytol non-stoichiometric magnetite)	Carboxymethyl dextran	Imaging of lymph nodes in patients with advanced rectal cancer	Clinical trial (Early phase I)	
Ferumoxytol (Feraheme^®^, Ferumoxytol non-stoichiometric magnetite)	Carboxymethyl dextran	Imaging of inflammatory (macrophage) responses in patients with malignant brain tumors	Clinical trial (Early phase I)	
Ferumoxytol (Feraheme™, Ferumoxytol non-stoichiometric magnetite)	Carboxymethyl dextran	Imaging of lymph nodes in patients with stage IIB-IIIC esophageal cancer	Clinical trial (Early phase I)	[317]
Ferumoxytol(Feraheme™)	Carboxymethyl dextran	Radiotherapy with SIONP on MR-Linac for hepatic cancers	Clinical trial (Observational)	
Ferumoxytol (Feraheme™, Ferumoxytol non-stoichiometric magnetite)	Carboxymethyl dextran	Imaging of lung carcinoma metastatic in the brain	Clinical trial (Phase II)	
Ferumoxytol (Feraheme^®^)	Carboxymethyl dextran	Imaging of prostate tumor	Clinical trial (Early Phase I)	
Ferumoxytol (AMAG Pharmaceuticals, Inc. (Waltham, MA, USA), Code 7228)	Carboxymethyl dextran	Imaging of brain tumors in Patients Receiving Chemotherapy	Clinical trial (Phase I)	
Ferumoxytol (Feraheme^®^)	Carboxymethyl dextran	Bone imaging	Clinical trial (Phase II)	
Ferumoxytol (Feraheme™, Ferumoxytol non-stoichiometric magnetite)	Carboxymethyl dextran	Imaging of glioblastoma tumors after treatment with pembrolizumab	Clinical trial (Phase II)	
Magnetic nanoparticles	anti-EpCAM or anti-CD52 antibodies	Removing of blood tumor cells in patients suffering from prostate, colon, lung, or pancreatic cancer	Clinical trial (Observational)	
Ferumoxytol (Feraheme^®^)	Carboxymethyl dextran	Imaging of abnormal lymph nodes in patients with thyroid cancer	Clinical trial (unknown status)	
Ferumoxytol (Feraheme^®^)	Carboxymethyl dextran	Imaging of lymph node metastases in pancreatic cancer	Clinical trial(phase IV)	
Ferumoxsil (Lumirem^®^, GastroMARK^®^, AMI-121)	Poly [N-(2-aminoethyl)-3- aminopropyl] siloxane	GI tract, abdominal tissue and bowel imaging	Used and discontinued	[273,318]
Ferumoxide (Feridex^®^, EndoremTM, AMI-25)	Dextran	Liver imaging	Used and discontinued	[67,318]
Ferucarbotran (Resovist^®^, CliavistTM, SHU 555A)	Carboxydextran	Liver/spleen imaging	Approved	[67,273]
Ferristene (Abdoscan^®^, OMP)	Polystyrene (sulfonated styrene-divinylbenzene copolymer)	Gastrointestinal, abdominal imaging	Approved and discontinued	[319]
Ferumoxtran-10 (Ferrotran^®^)	Dextran	Prostate imaging	Clinical trial (Phase III)	[317]
Ferumoxtran (Combidex^®^, Sinerem^®^)	Dextran	Lymph node imaging	Clinical trial	[67]
SIONP-epirubicin	Anhydroglucose	Magnetic targeted delivery of 4-epidoxorubicin into solid tumors	Clinical trials (terminated)	[320]
Nanotherm^®^ (MagForce)	Aminosilane	Magnetic hyperthermia on brain tumors	Approved/in use in Europe	[318,319]
MTC-Dox	Metallic iron and activated carbon	Magnetic drug targeting	Clinical trials (terminated)	[273,320]

OMP: Oral magnetic particles; SIONP: Superparamagnetic iron oxide nanoparticles; MTC: Magnetic targeted carrier.

## Data Availability

Not applicable.

## Abbreviations

((+)ly)	Positively
(NH_4_)_2_SO_4_	Ammonium sulfate
5-FU	5-Fluorouracil
ACA	Acrylic acid
ACV	Aciloclovir
Ag	Agar
AIBN	Azobisisobutyronitrile
Aly-Imz	Allyl imidazole
AMF	Alternating Magnetic Field
APS	Ammonium persulfate
Apt	Aptamer
APTES	3-aminopropyltriethoxysilane
ASGP	Ammonium sulfate gradient protocol
B-CD	B-cyclodextrin
BCT	Blood circulation time
BNPC	Bis(4-nitrophenyl)carbonate
C18TMS	Octadecyltrimethoxysilane
CDNSs	Cyclodextrin nanosponges
CDR	Controlled drug release
Cho	Cholesterol
CMCS	Carboxymethyl Chitosan
CMNP	Cationic magnetic nanoparticles
Co 6	Coumarin 6
CST	Critical Solution Temperature
CSC	Chitosan co-polymer
CTAB	Cetyltrimethylammonium bromide
Cu	Copper
CUR	Curcumin
DC	Dendritic cell
DD	Drug Delivery
DDS	Drug Delivery System
DESE	Double emulsion solvent evaporation
Dex	Dextran
DMSO	Dimethyl sulfoxide
DOX	Doxorubicin
DPPC	Dipalmitoylphosphatidylcholine
1,2-DPPC	1,2-dipalmitoyl-sn-glycero-3-phosphocholine
DSPC	1,2-distearoyl-sn-glycero-3-phosphocholine
DSPE-PEG2000	1,2-distearoyl-sn-glycero-3-phosphoethanolamine-N-[ami no(polyethylene-glycol)-2000]
EACCs	Ehrlich ascites carcinoma cells
EDA	ethylene diamine
EFR	Electric field-responsive
ENO1	Enolase 1
EPI	Epirubici
EPR	Enhance permeability retention affect
ER	Enzyme Responsive
ETB	Erlotinib
FA	Folic Acid
FDA	Food and drug administration
Fe	Iron
FE-SEM	Field emission scanning electron microscope
FITC	Fluorescein isothiocyanate
FMT	Ferumoxytol
FMT	Ferumoxytol
FRPR	Free radical polymerization reaction
FTIR	Fourier transform infrared
FTY720	Fingolimod
GaCCM	Gadolinium Consisting contrast medium
Ge	Gelatin
GM	Glioblastoma
GMC	Glioblastoma cancer
GSH	Glutathione
HA	Hyaluronic Acid
HAS	Human Serum albumin
HCC	Hepatocellular Carcinoma
HeLa	Human Cervical Carcinoma Cells
HEOC	Human epithelial ovarian carcinoma
HIFU	High Intensity focused Ultrasound
HPhBA	Hyperbranched phenylboronic acid
HRBC	Human red blood cells
HSO	Hydroxy silicon oil
HUVEC	Human Umbilical Vein Endothelial Cells
IONP	Iron oxide nanoparticle
IA	Itaconic acid
LCST	Lower Critical Solution Temperature
LF	Lactoferrin
LIFUS	low-intensity focused ultrasound
LiA	Lipoic acid
LMNPs	liposome magnetic nanoparticle
LR	Light Responsive
MaA	Maleic acid anhydride
Magnevist	Diethylenetriaminepentaacetic acid gadolinium(III) dihydrogen salt hydrate Gd(III)-DTPA
MB	Methylene Blue
MBAA	N,N-methylenebisacrylamide
MBBs	Microbubble
MDP	Muramyl dipeptide
MF	Magnetic field
MF-SPIO-NB	Multi-functional superparamagnetic iron oxide nano bubbles
M-HS	Magnetic-Hydrating Solution
MHT	Magnetic hypothermia
ML	Magnetic liposeme
MMP-2	Matrix metalloproteinase-2
MM-USI	Magnetomotive-ultrasound imaging
MNFs	magnetic nanofluids
MNP	Magnetic nano particle
MNS	Magnetic nano-system
MPNCs	Mesoporous nano-cage
MPDA	Mesoporous polydopamine
MPSNP	Mesoporous silica nanoparticles
M MPSNP	Magnetic Mesoporous silica nanoparticles
M MPS/MBBs	Magnetic Mesoporous silica microbubbles
MPTSA	4-Morpholineethanesulfonic acid
MRI	Magnetic resonance imaging
Ms	Saturation Magnetization
MSSM	Magnetic star structured micellar
MTRL	Magnetic Thermo-responsive liposome
MTX	Methotrexate
MUC1 Apt	Anti mucin aptamer
NADPH	Nicotinamide adenine dinucleotide phosphate
NB	Nanobubbles
nCP:Fe-CA	Fe-doped nano-calcium phosphate
NG	Nanogel
NH_2_-BDC	2-aminoterephthalic acid
NHS	N-hydroxy succinimide
NIPAM	N-isopropylacrylamide
NIR	Near infrared light
NP	Nano particle
NSCL	Non-small-cell-lung
O-CMCS	O-Carboxymethyl chitosan
OlA	Oleic Acid
OlAm	Oleylamine
OPSS-PEG-SVA	Orthopyridyl disulfide-poly(ethylene glycol)-succinimidyl valerate
PA	Photoacoustic
PhBA	Phenylboronic Acid
PBS	Phosphate-buffered saline
PCC	Pancake coil
PCL	Poly(ε-caprolactone)
PCL-diol	Polycaprolactone diol
PCL-g-Dex	poly(ε-caprolactone)-grafted dextran
PDA	Polydopamine
PDEAAm	Poly(N,N-diethylacrylamide)
PDI	Polydispersity index
PEG	Polyethylene Glycol
PEG-2000-DSPE	Poly-ethylene-glycol-2000-distearoyl-phosphatidyl-ethanolamine
PEG-PBA-PEG	poly (ethylene glycol)−Poly (butylene adipate)−poly (ethylene glycol)
PEG-PCL	Methoxy poly(ethylene glycol)-b-poly(ε-caprolactone)
PEI	Polyethyleneimine
PESM	Polyethersulfone membrane
PFP	Perfluoropentane
PG	Prodigiosin
pHR	pH responsive
PIMF	Polyethylenimine-rafted-poly (Malevich anhydride-alt-1-ocatadecene)-folic acid
PLH	Poly-L-histidine
PMX	Pemetrexed
PNIPAAm	Poly(N-isopropylacrylamide)
PNVCL	Poly (N-vinylcaprolactom)
PTC	Photothermal Conversion
PTT	Photothermal therapy
QDMAMEA	Quaternized ammonium alkyl halide N,N-dimethylaminoethyl methacrylate
R1/r2	Relaxivity
RES	Reticuloendothelial System
RF	Radio frequency
RFP	Red fluorescent probes
RGD	Arginylglycylaspartic
ROS	Reactive oxygen species
R-P	Receptor-Positive
RR	Redox Responsive
SA	Salic acid
SDS	Sodium dodecyl sulfate
SEM	Scanning electron microscopy
SH	Thiol
SiNc	Silicon naphthalocyanine
SIONP	Superparamagnetic iron oxide nano particle
SMNP	Smart magnetic nanoparticle
Sn(Oct)_2_	Stannous-2-ethylhexanoate Sn(Oct)2
SPECT	Single photo emission computed tomography
T1/T2	Transverse/Longitudinal Time
TDD	Targeted drug delivery
TDR	Targeted drug release
TEM	Transmission electron microscopy
Temp	Temperature
TEOS	Tetra-ethyl ortho-silicate
THF	Tetrahydrofuran
TME	Tumor micro environment
TMSMA	3-(Trimethoxysilyl) propyl methacrylate
TMZ	Temozolomide
TR	Thermo-responsive
UCST	Upper Critical Solution Temperature
U87 MG	Uppsala 87 Malignant Glioma
US	Ultrasound
UST	Ultrasound thermometry
US-TS	Ultrasound thermometry strain imaging
UV	Ultraviolet
Vs	Visible
VSM	Vibrating sample magnetometry
XRD	X-ray diffractometry
ZW	Zwitteronic 99mTc

## References

[1] Fitzmaurice C., Allen C., Barber R.M., Barregard L., Bhutta Z.A., Brenner H., Dicker D.J., Chimed-Orchir O., Dandona R., Dandona L. (2017). Global, Regional, and National Cancer Incidence, Mortality, Years of Life Lost, Years Lived with Disability, and Disability-Adjusted Life-years for 32 Cancer Groups, 1990 to 2015: A Systematic Analysis for the Global Burden of Disease Study. JAMA Oncol..

[2] Moein S., Adibi R., da Silva Meirelles L., Nardi N.B., Gheisari Y. (2020). Cancer regeneration: Polyploid cells are the key drivers of tumor progression. Biochim. Biophys Acta Rev. Cancer.

[3] Thitichai N., Thanapongpibul C., Theerasilp M., Sungkarat W., Nasongkla N. (2019). Study of biodistribution and systemic toxicity of glucose functionalized SPIO/DOX micelles. Pharm. Dev. Technol..

[4] Azamjah N., Soltan-Zadeh Y., Zayeri F. (2019). Global Trend of Breast Cancer Mortality Rate: A 25-Year Study. Asian Pac. J. Cancer Prev..

[5] Mattiuzzi C., Lippi G. (2019). Current Cancer Epidemiology. J. Epidemiol. Glob. Health.

[6] Sung H., Ferlay J., Siegel R.L., Laversanne M., Soerjomataram I., Jemal A., Bray F. (2021). Global Cancer Statistics 2020: GLOBOCAN Estimates of Incidence and Mortality Worldwide for 36 Cancers in 185 Countries. CA Cancer J. Clin..

[7] Salehiniya H., Ghobadi Dashdebi S., Rafiemanesh H., Mohammadian-Hafshejani A., Enayatrad M. (2016). Time Trend Analysis of Cancer Incidence in Caspian Sea, 2004–2009: A Population-based Cancer Registries Study (northern Iran). Casp. J. Intern. Med..

[8] Takeshima H., Ushijima T. (2019). Accumulation of genetic and epigenetic alterations in normal cells and cancer risk. NPJ Precis. Oncol..

[9] Morand G.B., da Silva S.D., Hier M.P., Alaoui-Jamali M.A. (2014). Insights into Genetic and Epigenetic Determinants with Impact on Vitamin D Signaling and Cancer Association Studies: The Case of Thyroid Cancer. Front. Oncol..

[10] Lewandowska A.M., Rudzki M., Rudzki S., Lewandowski T., Laskowska B. (2019). Environmental risk factors for cancer—Review paper. Ann. Agric. Environ. Med..

[11] Joung M.J., Han M.A., Park J., Ryu S.-Y. (2016). Association between Family and Friend Smoking Status and Adolescent Smoking Behavior and E-Cigarette Use in Korea. Int. J. Environ. Res. Public Health.

[12] Erenpreisa J., Salmina K., Anatskaya O., Cragg M.S. (2020). Paradoxes of cancer: Survival at the brink. Semin. Cancer Biol..

[13] Pienta K.J., Hammarlund E.U., Axelrod R., Amend S.R., Brown J.S. (2020). Convergent Evolution, Evolving Evolvability, and the Origins of Lethal Cancer. Mol. Cancer Res..

[14] Das M., Solanki A., Joshi A., Devkar R., Seshadri S., Thakore S. (2019). β-cyclodextrin based dual-responsive multifunctional nanotheranostics for cancer cell targeting and dual drug delivery. Carbohydr. Polym..

[15] Mancarella S., Greco V., Baldassarre F., Vergara D., Maffia M., Leporatti S. (2015). Polymer-Coated Magnetic Nanoparticles for Curcumin Delivery to Cancer Cells. Macromol. Biosci..

[16] Manatunga D.C., de Silva R.M., de Silva K.M.N., Malavige G.N., Wijeratne D.T., Williams G.R., Jayasinghe C.D., Udagama P.V. (2018). Effective delivery of hydrophobic drugs to breast and liver cancer cells using a hybrid inorganic nanocarrier: A detailed investigation using cytotoxicity assays, fluorescence imaging and flow cytometry. Eur. J. Pharm. Biopharm..

[17] Rahimi M., Safa K.D., Salehi R. (2017). Co-delivery of doxorubicin and methotrexate by dendritic chitosan-g-mPEG as a magnetic nanocarrier for multi-drug delivery in combination chemotherapy. Polym. Chem..

[18] Yetisgin A.A., Cetinel S., Zuvin M., Kosar A., Kutlu O. (2020). Therapeutic Nanoparticles and Their Targeted Delivery Applications. Molecules.

[19] Chamundeeswari M., Jeslin J., Verma M.L. (2018). Nanocarriers for drug delivery applications. Environ. Chem. Lett..

[20] Bai X.R., Wang L.H., Ren J.Q., Bai X.W., Zeng L.W., Shen A.G., Hu J.M. (2019). Accurate Clinical Diagnosis of Liver Cancer Based on Simultaneous Detection of Ternary Specific Antigens by Magnetic Induced Mixing Surface-Enhanced Raman Scattering Emissions. Anal. Chem..

[21] Satpathy M., Wang L., Zielinski R.J., Qian W., Wang Y.A., Mohs A.M., Kairdolf B.A., Ji X., Capala J., Lipowska M. (2019). Targeted Drug Delivery and Image-Guided Therapy of Heterogeneous Ovarian Cancer Using HER2-Targeted Theranostic Nanoparticles. Theranostics.

[22] Wei J., Shuai X., Wang R., He X.-l., Li Y., Ding M., Li J., Tan H., Fu Q. (2017). Clickable and imageable multiblock polymer micelles with magnetically guided and PEG-switched targeting and release property for precise tumor theranosis. Biomaterials.

[23] Ciofani G. (2018). Smart Nanoparticles for Biomedicine.

[24] Guo T., Lin M., Huang J., Zhou C., Tian W., Yu H., Jiang X., Ye J., Shi Y., Xiao Y. (2018). The Recent Advances of Magnetic Nanoparticles in Medicine. J. Nanomater..

[25] Soetaert F., Korangath P., Serantes D., Fiering S.N., Ivkov R. (2020). Cancer therapy with iron oxide nanoparticles: Agents of thermal and immune therapies. Adv. Drug Deliv. Rev..

[26] Assa F., Jafarizadeh-Malmiri H., Ajamein H., Anarjan N., Vaghari H., Sayyar Z., Berenjian A. (2016). A biotechnological perspective on the application of iron oxide nanoparticles. Nano Res..

[27] Sasikala A.R., GhavamiNejad A., Unnithan A.R., Thomas R.G., Moon M., Jeong Y.Y., Park C.H., Kim C.S. (2015). A smart magnetic nanoplatform for synergistic anticancer therapy: Manoeuvring mussel-inspired functional magnetic nanoparticles for pH responsive anticancer drug delivery and hyperthermia. Nanoscale.

[28] Arami H., Khandhar A., Liggitt D., Krishnan K.M. (2015). In vivo delivery, pharmacokinetics, biodistribution and toxicity of iron oxide nanoparticles. Chem. Soc. Rev..

[29] Barick K.C., Ekta E., Gawali S.L., Sarkar A., Kunwar A., Priyadarsini K.I., Hassan P.A. (2016). Pluronic stabilized Fe_3_O_4_ magnetic nanoparticles for intracellular delivery of curcumin. RSC Adv..

[30] Pourjavadi A., Dastanpour L., Tehrani Z.M. (2018). Magnetic micellar nanocarrier based on pH-sensitive PEG-PCL-PEG triblock copolymer: A potential carrier for hydrophobic anticancer drugs. J. Nanopart. Res..

[31] Sadr S.H., Davaran S., Alizadeh E., Salehi R., Ramazani A. (2018). PLA-based magnetic nanoparticles armed with thermo/pH responsive polymers for combination cancer chemotherapy. J. Drug Deliv. Sci. Technol..

[32] Pourjavadi A., Amin S.S., Hosseini S.H. (2018). Delivery of Hydrophobic Anticancer Drugs by Hydrophobically Modified Alginate Based Magnetic Nanocarrier. Ind. Eng. Chem. Res..

[33] Kang T., Li F., Baik S., Shao W., Ling D., Hyeon T. (2017). Surface design of magnetic nanoparticles for stimuli-responsive cancer imaging and therapy. Biomaterials.

[34] Pourjavadi A., Kohestanian M., Streb C. (2020). pH and thermal dual-responsive poly(NIPAM-co-GMA)-coated magnetic nanoparticles via surface-initiated RAFT polymerization for controlled drug delivery. Mater. Sci. Eng. C Mater. Biol. Appl..

[35] Garcia-Pinel B., Ortega-Rodriguez A., Porras-Alcala C., Cabeza L., Contreras-Caceres R., Ortiz R., Diaz A., Moscoso A., Sarabia F., Prados J. (2020). Magnetically active pNIPAM nanosystems as temperature-sensitive biocompatible structures for controlled drug delivery. Artif. Cells Nanomed. Biotechnol..

[36] Agrahari V., Agrahari V. (2018). Facilitating the translation of nanomedicines to a clinical product: Challenges and opportunities. Drug Discov. Today.

[37] Khandel P., Shahi S.K. (2018). Mycogenic nanoparticles and their bio-prospective applications: Current status and future challenges. J. Nanostruct. Chem..

[38] Rahmani R., Gharanfoli M., Gholamin M., Darroudi M., Chamani J., Sadri K., Hashemzadeh A. (2020). Plant-mediated synthesis of superparamagnetic iron oxide nanoparticles (SPIONs) using aloe vera and flaxseed extracts and evaluation of their cellular toxicities. Ceram. Int..

[39] Ullah A., Lim S.I. (2022). Plant extract-based synthesis of metallic nanomaterials, their applications, and safety concerns. Biotechnol. Bioeng..

[40] Majeed S., Danish M., Mohamad Ibrahim M.N., Sekeri S.H., Ansari M.T., Nanda A., Ahmad G. (2020). Bacteria Mediated Synthesis of Iron Oxide Nanoparticles and Their Antibacterial, Antioxidant, Cytocompatibility Properties. J. Clust. Sci..

[41] Vainshtein M., Belova N.S., Kulakovskaya T., Suzina N.E., Sorokin V.V. (2014). Synthesis of magneto-sensitive iron-containing nanoparticles by yeasts. J. Ind. Microbiol. Biotechnol..

[42] Satheeshkumar M.K., Kumar E.R., Indhumathi P., Srinivas C., Deepty M., Sathiyaraj S., Suriyanarayanan N., Sastry D.L. (2020). Structural, morphological and magnetic properties of algae/CoFe2O4 and algae/Ag-Fe-O nanocomposites and their biomedical applications. Inorg. Chem. Commun..

[43] Salem D.M.S.A., Ismail M.M., Aly-Eldeen M.A.-E. (2019). Biogenic synthesis and antimicrobial potency of iron oxide (Fe_3_O_4_) nanoparticles using algae harvested from the Mediterranean Sea, Egypt. Egypt. J. Aquat. Res..

[44] Mathur P., Saini S., Paul E., Sharma C., Mehtani P. (2021). Endophytic fungi mediated synthesis of iron nanoparticles: Characterization and application in methylene blue decolorization. Curr. Res. Green Sustain. Chem..

[45] Mahanty S., Bakshi M., Ghosh S., Chatterjee S., Bhattacharyya S., Das P., Das S., Chaudhuri P. (2019). Green Synthesis of Iron Oxide Nanoparticles Mediated by Filamentous Fungi Isolated from Sundarban Mangrove Ecosystem, India. BioNanoScience.

[46] Nie L., Cai C., Sun M., Zhang F., Zheng L., Peng Q., Shavandi A., Yang S. (2021). Iron Oxide Nanoparticles Synthesized Via Green Tea Extract for Doxorubicin Delivery. Curr. Nanosci..

[47] Bao Y., He J., Song K., Guo J., Zhou X., Liu S. (2021). Plant-Extract-Mediated Synthesis of Metal Nanoparticles. J. Chem..

[48] Hamdy N.M., Boseila A.A., Ramadan A.E., Basalious E.B. (2022). Iron Oxide Nanoparticles-Plant Insignia Synthesis with Favorable Biomedical Activities and Less Toxicity, in the “Era of the-Green”: A Systematic Review. Pharmaceutics.

[49] Salem S.S., Fouda A. (2020). Green Synthesis of Metallic Nanoparticles and Their Prospective Biotechnological Applications: An Overview. Biol. Trace Elem. Res..

[50] Nadeem M., Khan R., Shah N.R., Bangash I.R., Abbasi B.H., Hano C., Liu C., Ullah S., Hashmi S.S., Nadhman A. (2021). A Review of Microbial Mediated Iron Nanoparticles (IONPs) and Its Biomedical Applications. Nanomaterials.

[51] Mathur P., Jha S., Ramteke S., Jain N.K. (2018). Pharmaceutical aspects of silver nanoparticles. Artif. Cells Nanomed. Biotechnol..

[52] Singh P., Kim Y.-J., Zhang D., Yang D.-C. (2016). Biological Synthesis of Nanoparticles from Plants and Microorganisms. Trends Biotechnol..

[53] Klis F., Boorsma A., de Groot P.W.J. (2006). Cell wall construction in Saccharomyces cerevisiae. Yeast.

[54] Ma G., Zhao Z.-C., Liu H. (2016). Yeast Cells Encapsulating Polymer Nanoparticles as Trojan Particles via in Situ Polymerization inside Cells. Macromolecules.

[55] Fawcett D., Verduin J.J., Shah M., Sharma S., Poinern G.E.J. (2017). A Review of Current Research into the Biogenic Synthesis of Metal and Metal Oxide Nanoparticles via Marine Algae and Seagrasses. J. Nanosci..

[56] Chandran P.R., Naseer M.M., Udupa N., Sandhyarani N. (2012). Size controlled synthesis of biocompatible gold nanoparticles and their activity in the oxidation of NADH. Nanotechnology.

[57] Dahoumane S.A., Wujcik E.K., Jeffryes C.S. (2016). Noble metal, oxide and chalcogenide-based nanomaterials from scalable phototrophic culture systems. Enzym. Microb. Technol..

[58] Parial D., Pal R. (2014). Biosynthesis of monodisperse gold nanoparticles by green alga Rhizoclonium and associated biochemical changes. J. Appl. Phycol..

[59] Sodipo B.K., Aziz A.A. (2016). Recent advances in synthesis and surface modification of superparamagnetic iron oxide nanoparticles with silica. J. Magn. Magn. Mater..

[60] Arbain R., Othman M., Palaniandy S. (2011). Preparation of iron oxide nanoparticles by mechanical milling. Miner. Eng..

[61] Kurapov Y.A., Vazhnichaya E.M., Litvin S.E., Romanenko S.M., Didikin G.G., Devyatkina T.A., Mokliak Y.V., Oranskaya E.I. (2018). Physical synthesis of iron oxide nanoparticles and their biological activity in vivo. SN Appl. Sci..

[62] Scharf F., Mikhnevich E.A., Safronov A.P. (2017). Interaction of iron oxide nanoparticles synthesized by laser target evaporation with polyacrylamide in composites and ferrogels. Chim. Techno Acta.

[63] Sequeira C.A.C. (2018). Electrochemical Synthesis of Iron Oxide Nanoparticles for Biomedical Application. Org. Med. Chem. Int. J..

[64] Sorvali M., Nikka M., Juuti P., Honkanen M., Salminen T., Hyvärinen L., Mäkelä J.M. (2019). Controlling the phase of iron oxide nanoparticles fabricated from iron(III) nitrate by liquid flame spray. Int. J. Ceram. Eng. Sci..

[65] Sathya K., Saravanathamizhan R., Baskar G. (2017). Ultrasound assisted phytosynthesis of iron oxide nanoparticle. Ultrason. Sonochem..

[66] Rivera-Chaverra M.J., Restrepo-Parra E., Acosta-Medina C.D., Mello A., Ospina R. (2020). Synthesis of Oxide Iron Nanoparticles Using Laser Ablation for Possible Hyperthermia Applications. Nanomaterials.

[67] Dadfar S.M., Roemhild K., Drude N.I., von Stillfried S., Knüchel R., Kiessling F., Lammers T. (2019). Iron oxide nanoparticles: Diagnostic, therapeutic and theranostic applications. Adv. Drug. Deliv. Rev..

[68] Ali A., Shah T., Ullah R., Zhou P., Guo M., Ovais M., Tan Z., Rui Y. (2021). Review on Recent Progress in Magnetic Nanoparticles: Synthesis, Characterization, and Diverse Applications. Front. Chem..

[69] Teja A.S., Koh P.-Y. (2009). Synthesis, properties, and applications of magnetic iron oxide nanoparticles. Prog. Cryst. Growth Charact. Mater..

[70] Osial M., Rybicka P., kaBa M.P., Cichowicz G., CyrbDski M.K., KrysiDski P. (2018). Easy Synthesis and Characterization of Holmium-Doped SPIONs. Nanomaterials.

[71] Aende A., Gardy J., Aslam Z., Rogers M., Edokali M., Cespedes O., Harbottle D., Hassanpour A. (2022). A novel highly osmotic K/Fe_3_O_4_/CNF magnetic draw solution for salty water desalination. Desalination.

[72] Anastasiou A., Strafford S., Thomson C., Gardy J., Edwards T., Malinowski M., Hussain S., Metzger N., Hassanpour A., Brown C.J.A.B. (2018). Exogenous mineralization of hard tissues using photo-absorptive minerals and femto-second lasers; the case of dental enamel. Acta Biomater..

[73] Gardy J., Osatiashtiani A., Céspedes O., Hassanpour A., Lai X., Lee A.F., Wilson K., Rehan M. (2018). A magnetically separable SO_4_/Fe-Al-TiO_2_ solid acid catalyst for biodiesel production from waste cooking oil. J. Appl. Catal. B Environ..

[74] Gardy J., Nourafkan E., Osatiashtiani A., Lee A.F., Wilson K., Hassanpour A., Lai X. (2019). A core-shell SO_4_/Mg-Al-Fe_3_O_4_ catalyst for biodiesel production. J. Appl. Catal. B Environ..

[75] Hesas R.H., Baei M.S., Rostami H., Gardy J., Hassanpour A. (2019). An investigation on the capability of magnetically separable Fe_3_O_4_/mordenite zeolite for refinery oily wastewater purification. J. Environ. Manag..

[76] Besenhard M.O., LaGrow A.P., Hodzic A., Kriechbaum M., Panariello L., Bais G., Loizou K., Damilos S., Margarida Cruz M., Thanh N.T.K. (2020). Co-precipitation synthesis of stable iron oxide nanoparticles with NaOH: New insights and continuous production via flow chemistry. Chem. Eng. J..

[77] Besenhard M.O., Panariello L., Kiefer C., LaGrow A.P., Storozhuk L., Perton F., Bégin S., Mertz D., Thanh N.Å.T.K., Gavriilidis A. (2021). Small iron oxide nanoparticles as MRI T1 contrast agent: Scalable inexpensive water-based synthesis using a flow reactor. Nanoscale.

[78] Bhandari R., Gupta P., Dziubla T., Hilt J.Z. (2016). Single step synthesis, characterization and applications of curcumin functionalized iron oxide magnetic nanoparticles. Mater. Sci. Eng. C Mater. Biol. Appl..

[79] Fatima H., Kim K.-S. (2018). Iron-based magnetic nanoparticles for magnetic resonance imaging. Adv. Powder Technol..

[80] Majidi S., Sehrig F.Z., Farkhani S.M., Goloujeh M.S., Akbarzadeh A. (2016). Current methods for synthesis of magnetic nanoparticles. Artif. Cells Nanomed. Biotechnol..

[81] Cotin G., Kiefer C., Perton F., Ihiawakrim D., Blanco-Andujar C., Moldovan S., Lefevre C., Ersen O., Pichon B., Mertz D. (2018). Unravelling the Thermal Decomposition Parameters for The Synthesis of Anisotropic Iron Oxide Nanoparticles. Nanomaterials.

[82] Xie W., Guo Z., Gao F., Gao Q., Wang D., Liaw B.-S., Cai Q., Sun X., Wang X., Zhao L. (2018). Shape-, size- and structure-controlled synthesis and biocompatibility of iron oxide nanoparticles for magnetic theranostics. Theranostics.

[83] Liang Y.J., Zhang Y., Guo Z., Xie J., Bai T., Zou J., Gu N. (2016). Ultrafast Preparation of Monodisperse Fe_3_O_4_ Nanoparticles by Microwave-Assisted Thermal Decomposition. Chemistry.

[84] Gyergyek S., Makovec D., Jagodj M., Drofenik M., Schenk K., Jordan O., Kovb J., Drb~i G., Hofmann H. (2017). Hydrothermal growth of iron oxide NPs with a uniform size distribution for magnetically induced hyperthermia: Structural, colloidal and magnetic properties. J. Alloys Compd..

[85] Salvador M., Gutiérrez G., Noriega S., Moyano A., Blanco-López M.d.C., Matos M. (2021). Microemulsion Synthesis of Superparamagnetic Nanoparticles for Bioapplications. Int. J. Mol. Sci..

[86] Najafi A., Nematipour K. (2017). Synthesis and Magnetic Properties Evaluation of Monosized FeCo Alloy Nanoparticles Through Microemulsion Method. J. Supercond. Nov. Magn..

[87] Horikoshi S., Serpone N. (2013). Microwaves in Nanoparticle Synthesis: Fundamentals and Applications.

[88] Antone A.J., Sun Z., Bao Y. (2019). Preparation and Application of Iron Oxide Nanoclusters. Magnetochemistry.

[89] Wang P., Sun W., Guo J., Zhang K., Liu Y., Jiang Q., Su D.-K., Sun X. (2020). One pot synthesis of zwitteronic 99mTc doped ultrasmall iron oxide nanoparticles for SPECT/T1-weighted MR dual-modality tumor imaging. Colloids Surf. B Biointerfaces.

[90] Yoo D., Lee C., Seo B., Piao Y. (2017). One pot synthesis of amine-functionalized and angular-shaped superparamagnetic iron oxide nanoparticles for MR/fluorescence bimodal imaging application. RSC Adv..

[91] Pakapongpan S., Poo-arporn Y., Tuantranont A., Poo-arporn R.P. (2022). A facile one-pot synthesis of magnetic iron oxide nanoparticles embed N-doped graphene modified magnetic screen printed electrode for electrochemical sensing of chloramphenicol and diethylstilbestrol. Talanta.

[92] Al-Rawi N.N., Anwer B.A., Al-Rawi N.H., Uthman A.T., Ahmed I.S. (2020). Magnetism in drug delivery: The marvels of iron oxides and substituted ferrites nanoparticles. Saudi Pharm. J..

[93] Mohammed L., Gomaa H.G., Ragab D., Zhu J. (2017). Magnetic nanoparticles for environmental and biomedical applications: A review. Particuology.

[94] Veiseh O., Gunn J.W., Zhang M. (2010). Design and fabrication of magnetic nanoparticles for targeted drug delivery and imaging. Adv. Drug. Deliv. Rev..

[95] Zhou H., Yang H., Wang G., Gao A., Yuan Z. (2019). Recent Advances of Plasmonic Gold Nanoparticles in Optical Sensing and Therapy. Curr. Pharm. Des..

[96] Gotman I., Psakhie S.G., Lozhkomoev A.S., Gutmanas E.Y. (2016). Iron oxide and gold nanoparticles in cancer therapy. AIP Conf. Proc..

[97] Giménez C., de la Torre C., Gorbe M., Aznar E., Sancenón F., Murguía J.R., Martínez Máñez R., Marcos M.D., Amorós P. (2015). Gated mesoporous silica nanoparticles for the controlled delivery of drugs in cancer cells. Langmuir ACS J. Surf. Colloids.

[98] Liberman A., Mendez N., Trogler W.C., Kummel A.C. (2014). Synthesis and surface functionalization of silica nanoparticles for nanomedicine. Surf. Sci. Rep..

[99] Murugadoss S., Lison D., Godderis L., van den Brûle S., Mast J., Brassinne F., Sebaihi N., Hoet P.H.M. (2017). Toxicology of silica nanoparticles: An update. Arch. Toxicol..

[100] Zhong Q., Cao M., Hu H., Yang D., Chen M., Li P., Wu L., Zhang Q. (2018). One-Pot Synthesis of Highly Stable CsPbBr_3_@SiO_2_ Core-Shell Nanoparticles. ACS Nano.

[101] Hu G., Yang L., Li Y.n., Wang L. (2018). Continuous and scalable fabrication of stable and biocompatible MOF@SiO2 nanoparticles for drug loading. J. Mater. Chem. B.

[102] Foglia S., Ledda M., Fioretti D., Iucci G., Papi M., Capellini G., Lolli M.G., Grimaldi S., Rinaldi M., Lisi A. (2017). In vitro biocompatibility study of sub-U nm silica-coated magnetic iron oxide fluorescent nanoparticles for potential biomedical application. Sci. Rep..

[103] Jia L., Kitamoto Y. (2015). Influence of silica coating process on fine structure and magnetic properties of iron oxide nanoparticles. Electrochim. Acta.

[104] Saikia J., Yazdimamaghani M., Hadipour Moghaddam S.P., Ghandehari H. (2016). Differential Protein Adsorption and Cellular Uptake of Silica Nanoparticles Based on Size and Porosity. ACS Appl. Mater. Interfaces.

[105] Xue Y., Wu J., Sun J. (2012). Four types of inorganic nanoparticles stimulate the inflammatory reaction in brain microglia and damage neurons in vitro. Toxicol. Lett..

[106] Chen Q., Xue Y., Sun J. (2013). Kupffer cell-mediated hepatic injury induced by silica nanoparticles in vitro and in vivo. Int. J. Nanomed..

[107] Yu Y., Li Y., Wang W., Jin M., Du Z., Li Y., Duan J., Yu Y., Sun Z. (2013). Acute Toxicity of Amorphous Silica Nanoparticles in Intravenously Exposed ICR Mice. PLoS ONE.

[108] Zhuravskii S.G., Yukina G.Y., Kulikova O.O., Panevin A.S., Tomson V.V., Korolev D.V., Galagudza M.M. (2016). Mast cell accumulation precedes tissue fibrosis induced by intravenously administered amorphous silica nanoparticles. Toxicol. Mech. Methods.

[109] Limbach L.K., Wick P., Manser P., Grass R.N., Bruinink A., Stark W.J. (2007). Exposure of engineered nanoparticles to human lung epithelial cells: Influence of chemical composition and catalytic activity on oxidative stress. Environ. Sci. Technol..

[110] Boraschi D., Fadeel B., Duschl A. (2010). Nanoparticles and the immune system. Endocrinology.

[111] Jessop F., Hamilton R.F., Rhoderick J.F., Shaw P.K., Holian A. (2016). Autophagy deficiency in macrophages enhances NLRP3 inflammasome activity and chronic lung disease following silica exposure. Toxicol. Appl. Pharmacol..

[112] Park J.H., Jackman J.A., Ferhan A.R., Belling J.N., Mokrzecka N., Weiss P.S., Cho N.J. (2020). Cloaking Silica Nanoparticles with Functional Protein Coatings for Reduced Complement Activation and Cellular Uptake. ACS Nano.

[113] Shaterabadi Z., Nabiyouni G., Soleymani M. (2017). High impact of in situ dextran coating on biocompatibility, stability and magnetic properties of iron oxide nanoparticles. Mater. Sci. Eng. C Mater. Biol. Appl..

[114] Hein S., Wang K., Stevens W.F., Kjems J. (2008). Chitosan composites for biomedical applications: Status, challenges and perspectives. Mater. Sci. Technol..

[115] Hammad M., Hardt S., Mues B., Salamon S., Landers J., Slabu I., Wende H., Schulz C., Wiggers H. (2020). Gas-phase synthesis of iron oxide nanoparticles for improved magnetic hyperthermia performance. J. Alloys Compd..

[116] Tadros T.F. (2014). An Introduction to Surfactants.

[117] Poller J.M., Zaloga J., Schreiber E., Unterweger H., Janko C., Radon P., Eberbeck D., Trahms L., Alexiou C., Friedrich R.P. (2017). Selection of potential iron oxide nanoparticles for breast cancer treatment based on in vitro cytotoxicity and cellular uptake. Int. J. Nanomed..

[118] Gupta R., Pancholi K., De Sa R., Murray D., Huo D., Droubi G., White M.L., Njuguna J. (2019). Effect of Oleic Acid Coating of Iron Oxide Nanoparticles on Properties of Magnetic Polyamide-6 Nanocomposite. JOM.

[119] De Oliveira L.R., Rodrigues T.A., Costa H.L., da Silva Jr W.M. (2022). Scuffing resistance of polyalphaolefin (PAO)-based nanolubricants with oleic acid (OA) and iron oxide nanoparticles. Mater. Today Commun..

[120] Köçkar H., Karaagac O., Özel F. (2019). Effects of biocompatible surfactants on structural and corresponding magnetic properties of iron oxide nanoparticles coated by hydrothermal process. J. Magn. Magn. Mater..

[121] Lammers T., Subr V., Ulbrich K., Peschke P., Huber P.E., Hennink W.E., Storm G. (2009). Simultaneous delivery of doxorubicin and gemcitabine to tumors in vivo using prototypic polymeric drug carriers. Biomaterials.

[122] González E., Frey M.W. (2017). Synthesis, characterization and electrospinning of poly(vinyl caprolactam-co-hydroxymethyl acrylamide) to create stimuli-responsive nanofibers. Polymer.

[123] Rasool A., Ata S., Islam A. (2019). Stimuli responsive biopolymer (chitosan) based blend hydrogels for wound healing application. Carbohydr. Polym..

[124] Pormohammad A., Monych N.K., Ghosh S., Turner D.L., Turner R.J. (2021). Nanomaterials in Wound Healing and Infection Control. Antibiotics.

[125] Municoy S., Álvarez Echazú M.I., Antezana P.E., Galdopórpora J.M., Olivetti C.E., Mebert A.M., Foglia M.L., Tuttolomondo M.V., Álvarez G.S., Hardy J.G. (2020). Stimuli-Responsive Materials for Tissue Engineering and Drug Delivery. Int. J. Mol. Sci..

[126] Chatterjee S., Chi-Leung Hui P. (2019). Review of Stimuli-Responsive Polymers in Drug Delivery and Textile Application. Molecules.

[127] Wei M., Gao Y., Li X., Serpe M.J. (2017). Stimuli-responsive polymers and their applications. Polym. Chem..

[128] Contreras-Cáceres R., Cabeza L., Perazzoli G., Díaz A., López-Romero J.M., Melguizo C., Prados J. (2019). Electrospun Nanofibers: Recent Applications in Drug Delivery and Cancer Therapy. Nanomaterials.

[129] Das S.S., Bharadwaj P., Bilal M., Barani M., Rahdar A., Taboada P., Bungau S., Kyzas G.Z. (2020). Stimuli-Responsive Polymeric Nanocarriers for Drug Delivery, Imaging, and Theragnosis. Polymers.

[130] Hajebi S., Rabiee N., Bagherzadeh M., Ahmadi S., Rabiee M., Roghani-Mamaqani H., Tahriri M., Tayebi L., Hamblin M.R. (2019). Stimulus-responsive polymeric nanogels as smart drug delivery systems. Acta Biomater..

[131] Liu D., Yang F., Xiong F., Gu N. (2016). The Smart Drug Delivery System and Its Clinical Potential. Theranostics.

[132] Singh J., Kaur H. (2006). Stimuli-Responsive Materials: Thermo- and pH-Responsive Polymers for Drug Delivery. Adv. Drug Deliv. Rev..

[133] You Y., Kalebaila K.K., Brock S.L., Oupický D. (2008). Temperature-controlled uptake and release in PNIPAM-modified porous silica nanoparticles. Chem. Mater..

[134] Lim E.-K., Kim T., Paik S., Haam S., Huh Y.-M., Lee K. (2015). Nanomaterials for theranostics: Recent advances and future challenges. Chem. Rev..

[135] Turan S.K., Y1ld1zhan H., Barkan N.Q.n., Demiralp F.D.Ö., Uslu B., Ozkan S.A. (2018). Novel diagnostic techniques: Genomic, proteomic and systems biology approaches. Design of Nanostructures for Theranostics Applications.

[136] Hu W., Bai X., Wang Y., Lei Z., Luo H., Tong Z. (2019). Upper critical solution temperature polymer-grafted hollow mesoporous silica nanoparticles for near-infrared-irradiated drug release. J. Mater. Chem. B.

[137] Nandwana V., Ryoo S.R., Zheng T., You M.M., Dravid V.P. (2019). Magnetic Nanostructure-Coated Thermoresponsive Hydrogel Nanoconstruct As a Smart Multimodal Theranostic Platform. ACS Biomater. Sci. Eng..

[138] Raza A., Rasheed T., Nabeel F., Hayat U., Bilal M., Iqbal H.M.N. (2019). Endogenous and Exogenous Stimuli-Responsive Drug Delivery Systems for Programmed Site-Specific Release. Molecules.

[139] Rao N.V., Ko H., Lee J., Park J.H. (2018). Recent Progress and Advances in Stimuli-Responsive Polymers for Cancer Therapy. Front. Bioeng. Biotechnol..

[140] Ferjaoui Z., Jamal Al Dine E., Kulmukhamedova A., Bezdetnaya L., Soon Chang C., Schneider R., Mutelet F., Mertz D., Bégin-Colin S., Quilès F. (2019). Doxorubicin Loaded Thermo-responsive Superparamagnetic Nanocarriers for Controlled Drug Delivery and Magnetic Hyperthermia Applications. ACS Appl. Mater. Interfaces.

[141] Zhang Z., Wang Y., Rizk M.M.I., Liang R., Wells C.J.R., Gurnani P., Zhou F., Davies G.-L., Williams G.R. (2022). Thermo-responsive nano-in-micro particles for MRI-guided chemotherapy. Mater. Sci. Eng. C.

[142] Low L.E., Lim H.-P., Ong Y.S., Siva S.P., Sia C.S., Goh B.H., Chan E.S., Tey B.T. (2022). Stimuli-controllable iron oxide nanoparticle assemblies: Design, manipulation and bio-applications. J. Control. Release: Off. J. Control. Release Soc..

[143] Lakshmanan S., Gupta G.K., Avci P., Chandran R., Sadasivam M., Jorge A.E.S., Hamblin M.R. (2014). Physical energy for drug delivery; poration, concentration and activation. Adv. Drug Deliv. Rev..

[144] Maniotis N., Makridis A., Myrovali E., Theopoulos A., Samaras T., Angelakeris M. (2019). Magneto-mechanical action of multimodal field configurations on magnetic nanoparticle environments. J. Magn. Magn. Mater..

[145] Vangijzegem T., Stanicki D., Laurent S. (2019). Magnetic iron oxide nanoparticles for drug delivery: Applications and characteristics. Expert. Opin. Drug. Deliv..

[146] Lee K., David A.E., Zhang J., Shin M.C., Yang V.C. (2017). Enhanced accumulation of theranostic nanoparticles in brain tumor by external magnetic field mediated in situ clustering of magnetic nanoparticles. J. Ind. Eng. Chem..

[147] Wang J., Zhu X., Li C., Cai L., Pei W., Ni M., He J., Jiang H., Chen J. (2022). Efficient exosome extraction through the conjugation of superparamagnetic iron oxide nanoparticles for the targeted delivery in rat brain. Mater. Today Chem..

[148] Vilas-Boas V., Carvalho F., Espina B. (2020). Magnetic Hyperthermia for Cancer Treatment: Main Parameters Affecting the Outcome of In Vitro and In Vivo Studies. Molecules.

[149] Kobayashi T. (2011). Cancer hyperthermia using magnetic nanoparticles. Biotechnol. J..

[150] Del Sol-Fernández S., Portilla-Tundidor Y., Gutiérrez L., Odio O.F., Reguera E., Barber D.F., Morales M.P. (2019). Flower-like Mn-doped magnetic nanoparticles functionalized with α_v_β_3_-integrin-ligand to efficiently induce intracellular heat after AMF-exposition triggering glioma cell death. ACS Appl. Mater. Interfaces.

[151] Palzer J., Eckstein L., Slabu I., Reisen O., Neumann U.P., Roeth A.A. (2021). Iron Oxide Nanoparticle-Based Hyperthermia as a Treatment Option in Various Gastrointestinal Malignancies. Nanomaterials.

[152] Yang H., Jiang F., Zhang L., Wang L., Luo Y., Li N., Guo Y., Wang Q., Zou J. (2021). Multifunctional L-arginine-based magnetic nanoparticles for multiple-synergistic tumor therapy. Biomater. Sci..

[153] Tagami T., Foltz W., Ernsting M.J.E., Lee C.M., Tannock I.F., May J.P., Li S.-D. (2011). MRI monitoring of intratumoral drug delivery and prediction of the therapeutic effect with a multifunctional thermosensitive liposome. Biomaterials.

[154] Dan M., Bae Y., Pittman T.A., Yokel R.A. (2014). Alternating Magnetic Field-Induced Hyperthermia Increases Iron Oxide Nanoparticle Cell Association/Uptake and Flux in Bloo Brain Barrier Models. Pharm. Res..

[155] Carter T.J., Agliardi G., Lin F.-Y., Ellis M.J., Jones C., Robson M.P., Richard-Londt A., Southern P., Lythgoe M.F., Zaw Thin M. (2021). Potential of Magnetic Hyperthermia to Stimulate Localized Immune Activation. Small.

[156] Espinosa A., Di Corato R., Kolosnjaj-Tabi J., Flaud P., Pellegrino T., Wilhelm C. (2016). Duality of Iron Oxide Nanoparticles in Cancer Therapy: Amplification of Heating Efficiency by Magnetic Hyperthermia and Photothermal Bimodal Treatment. ACS Nano.

[157] Thorat N.D., Lemine O.M., Bohara R.A., Omri K., El Mir L., Tofail S.A.M. (2016). Superparamagnetic iron oxide nanocargoes for combined cancer thermotherapy and MRI applications. Phys. Chem. Chem. Phys. PCCP.

[158] Amin M., Huang W.-L., Seynhaeve A.L.B., ten Hagen T.L.M. (2020). Hyperthermia and Temperature-Sensitive Nanomaterials for Spatiotemporal Drug Delivery to Solid Tumors. Pharmaceutics.

[159] Shi D., Mi G., Shen Y., Webster T.J. (2019). Glioma-targeted dual functionalized thermosensitive Ferri-liposomes for drug delivery through an in vitro blood-brain barrier. Nanoscale.

[160] Habash R.W.Y., Bansal R., Krewski D., Alhafid H.T. (2006). Thermal therapy, part 2: Hyperthermia techniques. Crit. Rev. Biomed. Eng..

[161] Kumar C.S., Mohammad F. (2011). Magnetic nanomaterials for hyperthermia-based therapy and controlled drug delivery. Adv. Drug Deliv. Rev..

[162] Li J., Hu Y., Yang J., Wei P., Sun W., Shen M., Zhang G., Shi X. (2015). Hyaluronic acid-modified Fe_3_O_4_@Au core/shell nanostars for multimodal imaging and photothermal therapy of tumors. Biomaterials.

[163] Revia R.A., Zhang M. (2016). Magnetite nanoparticles for cancer diagnosis, treatment, and treatment monitoring: Recent advances. Mater. Today.

[164] Ray S., Cheng C.A., Chen W., Li Z., Zink J.I., Lin Y.Y. (2019). Magnetic Heating Stimulated Cargo Release with Dose Control using Multifunctional MR and Thermosensitive Liposome. Nanotheranostics.

[165] Asl H.M. (2017). Applications of Nanoparticles in Magnetic Resonance Imaging: A Comprehensive Review. Asian J. Pharm..

[166] Vuong Q.L., Gillis P., Roch A., Gossuin Y. (2017). Magnetic resonance relaxation induced by superparamagnetic particles used as contrast agents in magnetic resonance imaging: A theoretical review. Wiley Interdiscip. Rev. Nanomed. Nanobiotechnol..

[167] Buxton R.B. (2009). Introduction to Functional Magnetic Resonance Imaging: Principles and Techniques.

[168] Hu F., Zhao Y.S. (2012). Inorganic nanoparticle-based T1 and T1/T2 magnetic resonance contrast probes. Nanoscale.

[169] Caravan P., Ellison J.J., McMurry T.J., Lauffer R.B. (1999). Gadolinium(III) Chelates as MRI Contrast Agents: Structure, Dynamics, and Applications. Chem. Rev..

[170] Kanda T., Ishii K., Kawaguchi H., Kitajima K., Takenaka D. (2014). High signal intensity in the dentate nucleus and globus pallidus on unenhanced T1-weighted MR images: Relationship with increasing cumulative dose of a gadolinium-based contrast material. Radiology.

[171] Stojanov D., Aracki-Trenkj A., Benedeto-Stojanov D. (2016). Gadolinium deposition within the dentate nucleus and globus pallidus after repeated administrations of gadolinium-based contrast agent“ current status. Neuroradiology.

[172] Olchowy C., Cebulski K., Aasecki M., Chaber R., Olchowy A., KbBwak K., Zaleska-Dorobisz U. (2017). The presence of the gadolinium-based contrast agent depositions in the brain and symptoms of gadolinium neurotoxicity—A systematic review. PLoS ONE.

[173] Peng Y.K., Tsang S.C.E., Chou P.T. (2016). Chemical design of nanoprobes for T1-weighted magnetic resonance imaging. Mater. Today.

[174] Heine M., Bartelt A., Bruns O.T., Bargheer D., Giemsa A., Freund B., Scheja L., Waurisch C., Eychmüller A., Reimer R. (2014). The cell-type specific uptake of polymer-coated or micelle-embedded QDs and SPIOs does not provoke an acute pro-inflammatory response in the liver. Beilstein J. Nanotechnol..

[175] Sheel R., Kumari P., Panda P.K., Jawed Ansari M.D., Patel P., Singh S., Kumari B., Sarkar B., Mallick M.A., Verma S.K. (2020). Molecular intrinsic proximal interaction infer oxidative stress and apoptosis modulated in vivo biocompatibility of P.niruri contrived antibacterial iron oxide nanoparticles with zebrafish. Environ. Pollut..

[176] Kwon H.J., Shin K., Soh M., Chang H., Kim J., Lee J., Ko G., Kim B.H., Kim D., Hyeon T. (2018). Large-Scale Synthesis and Medical Applications of Uniform-Sized Metal Oxide Nanoparticles. Adv. Mater..

[177] Bao Y., Sherwood J.A., Sun Z. (2018). Magnetic iron oxide nanoparticles as T1 contrast agents for magnetic resonance imaging. J. Mater. Chem. C.

[178] Thanh N.T.K. (2018). Clinical Applications of Magnetic Nanoparticles: From Fabrication to Clinical Applications.

[179] Corot C., Robert P., Idee J.M., Port M. (2006). Recent advances in iron oxide nanocrystal technology for medical imaging. Adv. Drug. Deliv. Rev..

[180] Shu G., Chen M., Song J., Xu X., Lu C., Du Y., Xu M., Zhao Z., Zhu M., Fan K. (2021). Sialic acid-engineered mesoporous polydopamine nanoparticles loaded with SPIO and Fe. Bioact. Mater..

[181] Fernández-Barahona I., Muñoz-Hernando M., Ruiz-Cabello J., Herranz F., Pellico J. (2020). Iron Oxide Nanoparticles: An Alternative for Positive Contrast in Magnetic Resonance Imaging. Inorganics.

[182] Lu J., Sun J., Li F., Wang J., Liu J., Kim D., Fan C., Hyeon T., Ling D. (2018). Highly Sensitive Diagnosis of Small Hepatocellular Carcinoma Using pH-Responsive Iron Oxide Nanocluster Assemblies. J. Am. Chem. Soc..

[183] Wang L., Yin H., Bi R., Gao G., Li K., Liu H.-L. (2020). ENOQ targeted superparamagnetic iron oxide nanoparticles for detecting pancreatic cancer by magnetic resonance imaging. J. Cell. Mol. Med..

[184] Sridharan B., Devarajan N., Jobanputra R.B., Gowd G.S., Anna I.M., Ashokan A., Nair S.V., Koyakutty M. (2021). nCP:Fe Nanocontrast Agent for Magnetic Resonance Imaging-Based Early Detection of Liver Cirrhosis and Hepatocellular Carcinoma. ACS Appl. Bio Mater..

[185] Anna I.M., Sathy B.N., Ashokan A., Gowd G.S., Ramachandran R.K., Kochugovindan Unni A.K., Manohar M., Chulliyath D., Nair S.V., Bhakoo K.K. (2019). nCP: A Biomineral Magnetic Nanocontrast Agent for Tracking Implanted Stem Cells in Brain Using MRI. ACS Appl. Bio Mater..

[186] Uppalapati D., Boyd B.J., Garg S., Travas-sejdic J., Svirskis D.M. (2016). Conducting polymers with defined micro- or nanostructures for drug delivery. Biomaterials.

[187] Casella A., Panitch A., Leach J.K. (2021). Endogenous Electric Signaling as a Blueprint for Conductive Materials in Tissue Engineering. Bioelectricity.

[188] Kotnik T., Rems L., Tarek M., Miklavčič D. (2019). Membrane Electroporation and Electropermeabilization: Mechanisms and Models. Annu. Rev. Biophys..

[189] Prakash P., Srimathveeravalli G. (2021). Principles and Technologies for Electromagnetic Energy Based Therapies.

[190] Viratchaiboott N., Sakunpongpitiporn P., Niamlang S., Sirivat A. (2022). Release of 5-FU Loaded Pectin/Fe_3_O_4_ from Porous PBSA Matrix under Magnetic and Electric Fields. J. Alloys Compd..

[191] Benson L. (2018). Tumor Treating Fields Technology: Alternating Electric Field Therapy for the Treatment of Solid Tumors. Semin. Oncol. Nurs..

[192] Raza A., Hayat U., Rasheed T., Bilal M., Iqbal H.M.N. (2018). Redox-responsive nano-carriers as tumor-targeted drug delivery systems. Eur. J. Med. Chem..

[193] Molaei H., Zaaeri F., Sharifi S., Ramazani A., Safaei S., Abdolmohammadi J., Khoobi M. (2020). Polyethylenimine-graft-poly (maleic anhydride-alt-1-octadecene) coated Fe_3_O_4_ magnetic nanoparticles: Promising targeted pH-sensitive system for curcumin delivery and MR imaging. Int. J. Polym. Mater. Polym. Biomater..

[194] Nalluri L.P., Popuri S.R., Lee C.-H., Terbish N. (2020). Synthesis of biopolymer coated functionalized superparamagnetic iron oxide nanoparticles for the pH-sensitive delivery of anti-cancer drugs epirubicin and temozolomide. Int. J. Polym. Mater. Polym. Biomater..

[195] Banstola A., Poudel K., Kim J.O., Jeong J.H., Yook S. (2021). Recent progress in stimuli-responsive nanosystems for inducing immunogenic cell death. J. Control. Release Off. J. Control. Release Soc..

[196] Saadat M., Mostafaei F., Mahdinloo S., Abdi M., Zahednezhad F., Zakeri-Milani P., Valizadeh H. (2021). Drug delivery of pH-Sensitive nanoparticles into the liver cancer cells. J. Drug Deliv. Sci. Technol..

[197] Rosenblum D., Joshi N., Tao W., Karp J.M., Peer D. (2018). Progress and challenges towards targeted delivery of cancer therapeutics. Nat. Commun..

[198] Guo X., Cheng Y., Zhao X., Luo Y., Chen J., Yuan W.-E. (2018). Advances in redox-responsive drug delivery systems of tumor microenvironment. J. Nanobiotechnol..

[199] Tang Z., Zhang L., Wang Y., Li D., Zhong Z., Zhou S. (2016). Redox-responsive star-shaped magnetic micelles with active-targeted and magnetic-guided functions for cancer therapy. Acta Biomater..

[200] Chen L., Xue Y., Xia X.-M., Song M., Huang J., Zhang H., Yu B., Long S., Liu Y., Liu L. (2015). A redox stimuli-responsive superparamagnetic nanogel with chemically anchored DOX for enhanced anticancer efficacy and low systemic adverse effects. J. Mater. Chem. B.

[201] Mousavi S.D., Maghsoodi F., Panahandeh F., Yazdian-Robati R., Reisi-Vanani A., Tafaghodi M. (2018). Doxorubicin delivery via magnetic nanomicelles comprising from reduction-responsive poly(ethylene glycol)bpoly(epsiloncaprolactone) (PEG-SS-PCL) and loaded with superparamagnetic iron oxide (SPIO) nanoparticles: Preparation, characterization and simulation. Mater. Sci. Eng. C Mater. Biol. Appl..

[202] Yang H.Y., Jang M.S., Li Y., Lee J.H., Lee D.S. (2017). Multifunctional and Redox-Responsive Self-Assembled Magnetic Nanovectors for Protein Delivery and Dual-Modal Imaging. ACS Appl. Mater. Interfaces.

[203] Shang L., Wang Q., Chen K., Qu J., Lin J., Luo J., Zhou Q. (2017). Preparation of polydopamine based redox-sensitive magnetic nanoparticles for doxorubicin delivery and MRI detection. J. Bioresour. Bioprod..

[204] Gisbert-Garzarán M., Valle Regí M. (2021). Redox-Responsive Mesoporous Silica Nanoparticles for Cancer Treatment: Recent Updates. Nanomaterials.

[205] Nguyen M.M., Carlini A.S., Chien M.P., Sonnenberg S., Luo C., Braden R.L., Osborn K.G., Li Y., Gianneschi N.C., Christman K.L. (2015). Enzyme-Responsive Nanoparticles for Targeted Accumulation and Prolonged Retention in Heart Tissue after Myocardial Infarction. Adv. Mater..

[206] Ramasamy T., Ruttala H.B., Gupta B., Poudel B.K., Choi H.G., Yong C.S., Kim J.O. (2017). Smart chemistry-based nanosized drug delivery systems for systemic applications: A comprehensive review. J. Control. Release.

[207] Qiao Y., Wan J., Zhou L., Ma W., Yang Y., Luo W., Yu Z., Wang H. (2019). Stimuli-responsive nanotherapeutics for precision drug delivery and cancer therapy. Wiley Interdiscip. Rev. Nanomed. Nanobiotechnol..

[208] Li E., Yang Y., Hao G., Yi X., Zhang S., Pan Y., Xing B., Gao M. (2018). Multifunctional Magnetic Mesoporous Silica Nanoagents for in vivo Enzyme-Responsive Drug Delivery and MR Imaging. Nanotheranostics.

[209] Nosrati H., Mojtahedi A., Danafar H., Kheiri Manjili H. (2018). Enzymatic stimuli-responsive methotrexate-conjugated magnetic nanoparticles for target delivery to breast cancer cells and release study in lysosomal condition. J. Biomed. Mater. Research. Part A.

[210] Rastegari B., Karbalaei-Heidari H.R., Zeinali S., Sheardown H. (2017). The enzyme-sensitive release of prodigiosin grafted β-cyclodextrin and chitosan magnetic nanoparticles as an anticancer drug delivery system: Synthesis, characterization and cytotoxicity studies. Colloids Surf. B Biointerfaces.

[211] Hu Q., Katti P.S., Gu Z. (2014). Enzyme-responsive nanomaterials for controlled drug delivery. Nanoscale.

[212] Khan M.F., Kundu D., Gogoi M., Shrestha A.K., Karanth N.G., Patra S. (2020). Enzyme-Responsive and Enzyme Immobilized Nanoplatforms for Therapeutic Delivery: An Overview of Research Innovations and Biomedical Applications. Princ. Appl..

[213] Navya P.N., Kaphle A., Srinivas S.P., Bhargava S.K., Rotello V.M., Daima H.K. (2019). Current trends and challenges in cancer management and therapy using designer nanomaterials. Nano Converg..

[214] Li M., Zhao G., Su W., Shuai Q. (2020). Enzyme-Responsive Nanoparticles for Anti-tumor Drug Delivery. Front. Chem..

[215] Meinhardt M., Krebs R., Anders A., Heinrich U., Tronnier H.P.D. (2008). Wavelength-dependent penetration depths of ultraviolet radiation in human skin. J. Biomed. Opt..

[216] Zhou J., Gai L., Zhou Z., Yang W., Mack J., Xu K., Zhao J., Zhao Y., Qiu H., Chan K.S. (2016). Rational Design of Emissive NIR-Absorbing Chromophores: Rh(III) Porphyrin-Aza-BODIPY Conjugates with Orthogonal Metal-Carbon Bonds. Chemistry.

[217] Zhang L., Guan X., Xiao X., Chai Y., Chen Z., Zhou G., Fan Y. (2021). Near-infrared triggered injectable ferrimagnetic chitosan thermosensitive hydrogel for photo hyperthermia and precisely controlled drug release in tumor ablation. Eur. Polym. J..

[218] Feng Q., Zhang Y., Zhang W., Hao Y., Wang Y., Zhang H., Hou L., Zhang Z. (2017). Programmed near-infrared light-responsive drug delivery system for combined magnetic tumor-targeting magnetic resonance imaging and chemo-phototherapy. Acta Biomater..

[219] Eyvazzadeh N., Shakeri-Zadeh A., Fekrazad R., Amini E., Ghaznavi H., Kamran Kamrava S. (2017). Gold-coated magnetic nanoparticle as a nanotheranostic agent for magnetic resonance imaging and photothermal therapy of cancer. Lasers Med. Sci..

[220] Kuo S.H., Wu P.T., Huang J.Y., Chiu C.P., Yu J., Liao M.Y. (2020). Fabrication of Anisotropic Cu Ferrite-Polymer Core-Shell Nanoparticles for Photodynamic Ablation of Cervical Cancer Cells. Nanomaterials.

[221] Delalande A., Bastié C., Pigeon L., Manta S., Lebertre M., Mignet N., Midoux P., Pichon C. (2017). Cationic gas-filled microbubbles for ultrasound-based nucleic acids delivery. Biosci. Rep..

[222] Kooiman K., Roovers S., Langeveld S.A.G., Kleven R.T., Dewitte H., Reilly M.A.O., Escoffre J.-M., Bouakaz A., Verweij M.D., Hynynen K. (2020). Ultrasound-Responsive Cavitation Nuclei for Therapy and Drug Delivery. Ultrasound Med. Biol..

[223] Inserra C., Regnault G., Cleve S., Mauger C., Blanc-Benon P. (2021). Induction of Microstreaming by Nonspherical Bubble Oscillations in an Acoustic Levitation System. J. Vis. Exp. JoVE.

[224] Du M., Chen Y., Tu J., Liufu C., Yu J., Yuan Z., Gong X., Chen Z. (2020). Ultrasound Responsive Magnetic Mesoporous Silica Nanoparticle-Loaded Microbubbles for Efficient Gene Delivery. ACS Biomater. Sci. Eng..

[225] Tang Y., Wang G. (2021). NIR light-responsive nanocarriers for controlled release. J. Photochem. Photobiol. C-Photochem. Rev..

[226] Nesbitt H., Sheng Y., Kamila S., Logan K.A., Thomas K.G., Callan B., Taylor M.A., Love M., O’Rourke D., Kelly P. (2018). Gemcitabine loaded microbubbles for targeted chemo sonodynamic therapy of pancreatic cancer. J. Control. Release.

[227] Snipstad S., Berg S., Mørch Ý., Bjørkøy A., Sulheim E., Hansen R., Grimstad I., van Wamel A., Maaland A.F., Torp S.H. (2017). Ultrasound Improves the Delivery and Therapeutic Effect of Nanoparticle-Stabilized Microbubbles in Breast Cancer Xenografts. Ultrasound Med. Biol..

[228] Bhattacharya D., Behera B., Sahu S.K., Ananthakrishnan R., Maiti T.K., Pramanik P. (2016). Design of dual stimuli responsive polymer modified magnetic nanoparticles for targeted anti-cancer drug delivery and enhanced MR imaging. New J. Chem..

[229] Kaczmarek K., Hornowski T., Kubovcíková M., Timko M., Koralewski M., Józefczak A. (2018). Heating Induced by Therapeutic Ultrasound in the Presence of Magnetic Nanoparticles. ACS Appl. Mater. Interfaces.

[230] Mahmoudi K., Bouras A., Bozec D., Ivkov R., Hadjipanayis C. (2018). Magnetic hyperthermia therapy for the treatment of glioblastoma: A review of the therapy’s history, efficacy and application in humans. Int. J. Hyperth..

[231] Zhu L., Altman M.B., Laszlo A., Straube W.L., Zoberi I., Hallahan D., Chen H. (2019). Ultrasound Hyperthermia Technology for Radiosensitization. Ultrasound Med. Biol..

[232] Sengupta S., Balla V.K. (2018). A review on the use of magnetic fields and ultrasound for non-invasive cancer treatment. J. Adv. Res..

[233] Hadadian Y., Uliana J.H., Carneiro A.A.O., Pavan T.Z. (2021). A Novel Theranostic Platform: Integration of Magnetomotive and Thermal Ultrasound Imaging With Magnetic Hyperthermia. IEEE Trans. Biomed. Eng..

[234] Shalaby T., Gawish A., Hamad H. (2021). A Promising Platform of Magnetic Nanofluid and Ultrasonic Treatment for Cancer Hyperthermia Therapy: In Vitro and in Vivo Study. Ultrasound. Med. Biol..

[235] Rühle B., Datz S., Argyo C., Bein T., Zink J.I. (2016). A molecular nanocap activated by superparamagnetic heating for externally stimulated cargo release. Chem. Commun..

[236] Albarqi H.A., Demessie A.A., Sabei F.Y., Moses A.S., Hansen M.N., Dhagat P., Taratula O.R., Taratula O. (2020). Systemically Delivered Magnetic Hyperthermia for Prostate Cancer Treatment. Pharmaceutics.

[237] Issels R.D., Lindner L.H., Verweij J., Wessalowski R., Reichardt P., Wust P., Ghadjar P., Hohenberger P., Angele M., Salat C. (2018). Effect of Neoadjuvant Chemotherapy Plus Regional Hyperthermia on Long-term Outcomes Among Patients With Localized High-Risk Soft Tissue Sarcoma: The EORTC 62961-ESHO 95 Randomized Clinical Trial. JAMA Oncol..

[238] Chen B., Zhang R., Wu H., Li M., Zhou G., Ji M. (2020). Thermoresponsive magnetoliposome encapsulating doxorubicin and high performance Ferumoxytol for effective tumor synergistic therapy in vitro. J. Drug Deliv. Sci. Technol..

[239] Guo Y., Zhang Y., Ma J., Li Q., Li Y., Zhou X., Zhao D., Song H., Chen Q., Zhu X. (2018). Light/magnetic hyperthermia triggered drug released from multi-functional thermo-sensitive magnetoliposomes for precise cancer synergetic theranostics. J. Control. Release.

[240] Pramanik N., Ranganathan S., Rao S., Suneet K., Jain S., Rangarajan A., Jhunjhunwala S. (2019). A Composite of Hyaluronic Acid-Modified Graphene Oxide and Iron Oxide Nanoparticles for Targeted Drug Delivery and Magnetothermal Therapy. ACS Omega.

[241] Aseyev V., Tenhu H., Winnik F.M. (2010). Non-Ionic Thermoresponsive Polymers in Water.

[242] Pourjavadi A., Mazaheri Tehrani Z., Dastanpour L. (2018). Smart magnetic self-assembled micelle: An effective nanocarrier for thermo-triggered paclitaxel delivery. Int. J. Polym. Mater. Polym. Biomater..

[243] Afzalipour R., Khoei S., Khoee S., Shirvalilou S., Raoufi N.J., Motevalian M., Karimi M.Y. (2021). Thermosensitive magnetic nanoparticles exposed to alternating magnetic field and heat-mediated chemotherapy for an effective dual therapy in rat glioma model. Nanomedicine.

[244] Dutta B., Shetake N.G., Barick B.K., Barick K.C., Pandey B.N., Priyadarsini K.I., Hassan P.A. (2018). pH sensitive surfactant-stabilized Fe. Colloids Surf. B Biointerfaces.

[245] Matos R.J.R., Chaparro C.I.P., Silva J.C., Valente M.A., Borges J.P., Soares P.I.P. (2018). Electrospun composite cellulose acetate/iron oxide nanoparticles non-woven membranes for magnetic hyperthermia applications. Carbohydr. Polym..

[246] Farshbaf M., Salehi R., Annabi N., Khalilov R., Akbarzadeh A., Davaran S. (2018). pH- and thermo-sensitive MTX-loaded magnetic nanocomposites: Synthesis, characterization, and in vitro studies on A549 lung cancer cell and MR imaging. Drug. Dev. Ind. Pharm..

[247] Alijani H., Noori A., Faridi N., Bathaie S.Z., Mousavi M.F. (2020). Aptamer-functionalized Fe_3_O_4_@MOF nanocarrier for targeted drug delivery and fluorescence imaging of the triple-negative MDA-MB-231 breast cancer cells. J. Solid State Chem..

[248] Gholibegloo E., Mortezazadeh T., Salehian F., Forootanfar H., Firoozpour L., Foroumadi A., Ramazani A., Khoobi M. (2019). Folic acid decorated magnetic nanosponge: An efficient nanosystem for targeted curcumin delivery and magnetic resonance imaging. J. Colloid. Interface Sci..

[249] Ali A.A., Hsu F.T., Hsieh C.L., Shiau C.Y., Chiang C.H., Wei Z.H., Chen C.Y., Huang H.S. (2016). Erlotinib-Conjugated Iron Oxide Nanoparticles as a Smart Cancer-Targeted Theranostic Probe for MRI. Sci. Rep..

[250] Abedi M., Abolmaali S.S., Abedanzadeh M., Farjadian F., Mohammadi Samani S., Tamaddon A.M. (2020). Core-Shell Imidazoline-Functionalized Mesoporous Silica Superparamagnetic Hybrid Nanoparticles as a Potential Theranostic Agent for Controlled Delivery of Platinum(II) Compound. Int. J. Nanomed..

[251] Li D., Zhang M., Xu F., Chen Y., Chen B.-f., Chang Y., Zhong H., Jin H., Huang Y. (2018). Biomimetic albumin-modified gold nanorods for photothermo-chemotherapy and macrophage polarization modulation. Acta Pharm. Sin. B.

[252] Deng X., Liang H., Yang W., Shao Z. (2020). Polarization and function of tumor-associated macrophages mediate graphene oxide-induced photothermal cancer therapy. J. Photochem. Photobiol. B Biol..

[253] Guo X.-M., Chen J.-L., Zeng B.-H., Lai J.-C., Lin C.-Y., Lai M.-Y. (2020). Ultrasound-mediated delivery of RGD-conjugated nanobubbles loaded with fingolimod and superparamagnetic iron oxide nanoparticles: Targeting hepatocellular carcinoma and enhancing magnetic resonance imaging. RSC Adv..

[254] Licciardi M., Scialabba C., Puleio R., Cassata G., Cicero L., Cavallaro G., Giammona G. (2019). Smart copolymer coated SPIONs for colon cancer chemotherapy. Int. J. Pharm..

[255] Li D., Deng M., Yu Z., Liu W., Zhou G., Li W., Wang X., Yang D.P., Zhang W. (2018). Biocompatible and Stable GO-Coated Fe_3_O_4_ Nanocomposite: A Robust Drug Delivery Carrier for Simultaneous Tumor MR Imaging and Targeted Therapy. ACS Biomater. Sci. Eng..

[256] Ghorbani M., Bigdeli B., Jalili-Baleh L., Baharifar H., Akrami M., Dehghani S., Goliaei B., Amani A., Lotfabadi A., Rashedi H. (2018). Curcumin-lipoic acid conjugate as a promising anticancer agent on the surface of goldiron oxide nanocomposites: A pH-sensitive targeted drug delivery system for brain cancer theranostics. Eur. J. Pharm. Sci..

[257] Wang M., Yang Q., Li M., Zou H., Wang Z., Ran H., Zheng Y., Jian J., Zhou Y., Luo Y. (2020). Multifunctional Nanoparticles for Multimodal Imaging-Guided Low-Intensity Focused Ultrasound/Immunosynergistic Retinoblastoma Therapy. ACS Appl. Mater. Interfaces.

[258] Wang X., Huang P., Liu H., Li C., Shen G., Cui D. (2013). Metal ion-directed solution-phase tailoring: From large-area graphene oxide into nanoscale pieces. Nanoscale Res. Lett..

[259] Gao F., Wu X., Wu D., Yu J., Yao J., Qi Q., Cao Z., Cui Q., Mi Y. (2020). Preparation of degradable magnetic temperature- and redox-responsive polymeric/Fe_3_O_4_ nanocomposite nanogels in inverse miniemulsions for loading and release of 5-fluorouracil. Colloids Surf. A Physicochem. Eng. Asp..

[260] Oh Y., Je J.Y., Moorthy M.S., Seo H., Cho W.H. (2017). pH and NIR-light-responsive magnetic iron oxide nanoparticles for mitochondria-mediated apoptotic cell death induced by chemo-photothermal therapy. Int. J. Pharm..

[261] Lin X., Song X., Zhang Y., Cao Y., Xue Y., Wu F., Yu F., Wu M., Zhu X. (2020). Multifunctional theranostic nanosystems enabling photothermal-chemo combination therapy of triple-stimuli-responsive drug release with magnetic resonance imaging. Biomater. Sci..

[262] Luo Y., Yang H., Zhou Y.-f., Hu B. (2019). Dual and multi-targeted nanoparticles for site-specific brain drug delivery. J. Control. Release Off. J. Control. Release Soc..

[263] Nag O.K., Delehanty J.B. (2019). Active Cellular and Subcellular Targeting of Nanoparticles for Drug Delivery. Pharmaceutics.

[264] Mair L.O., Adam G., Chowdhury S., Davis A., Arifin D.R., Vassoler F.M., Engelhard H.H., Li J., Tang X., Weinberg I.N. (2021). Soft Capsule Magnetic Millirobots for Region-Specific Drug Delivery in the Central Nervous System. Front. Robot. AI.

[265] Tietjen G.T., Bracaglia L.G., Saltzman W.M., Pober J.S. (2018). Focus on Fundamentals: Achieving Effective Nanoparticle Targeting. Trends Mol. Med..

[266] Lopalco A., Cutrignelli A., Denora N., Lopedota A., Franco M., Laquintana V. (2018). Transferrin Functionalized Liposomes Loading Dopamine HCl: Development and Permeability Studies across an In Vitro Model of Human Blood-Brain Barrier. Nanomaterials.

[267] Banerjee D., Harfouche R., Sengupta S. (2011). Nanotechnology-mediated targeting of tumor angiogenesis. Vasc. Cell.

[268] Attia M.F., Anton N., Wallyn J., Omran Z., Vandamme T. (2019). An overview of active and passive targeting strategies to improve the nanocarriers efficiency to tumour sites. J. Pharm. Pharmacol..

[269] Bazak R., Houri M.T., Achy S.E., Hussein W., Refaat T. (2014). Passive targeting of nanoparticles to cancer: A comprehensive review of the literature. Mol. Clin. Oncol..

[270] Cao Y. (2005). Emerging mechanisms of tumour lymphangiogenesis and lymphatic metastasis. Nat. Rev. Cancer.

[271] Narum S.M., Le T., Le D.-P., Lee J.C., Donahue N.D., Yang W., Wilhelm S. (2020). Passive Targeting in Nanomedicine: Fundamental Concepts, Body Interactions, and Clinical Potential.

[272] Albinali K.E., Zagho M.M., Deng Y., Elzatahry A.A. (2019). A perspective on magnetic core-shell carriers for responsive and targeted drug delivery systems. Int. J. Nanomed..

[273] El-Boubbou K. (2018). Magnetic iron oxide nanoparticles as drug carriers: Preparation, conjugation and delivery. Nanomedicine.

[274] Jurczyk M., Jelonek K., MusibB-Kulik M., Beberok A., Wrześniok D., Kasperczyk J. (2021). Single- versus Dual-Targeted Nanoparticles with Folic Acid and Biotin for Anticancer Drug Delivery. Pharmaceutics.

[275] Raj S., Khurana S., Choudhari R., Kesari K.K., Kamal M.A., Garg N., Ruokolainen J., Das B.C., Kumar D. (2021). Specific targeting cancer cells with nanoparticles and drug delivery in cancer therapy. Semin. Cancer Biol..

[276] Ghorbani M., Zarei M., Mahmoodzadeh F., Ghorbani M. (2020). Targeted delivery of methotrexate using a new PEGylated magnetic/gold nanoplatform covered with pH-responsive shell. Int. J. Polym. Mater. Polym. Biomater..

[277] Avedian N., Zaaeri F., Daryasari M.P., Akbari Javar H., Khoobi M. (2018). pH-sensitive biocompatible mesoporous magnetic nanoparticles labeled with folic acid as an efficient carrier for controlled anticancer drug delivery. J. Drug Deliv. Sci. Technol..

[278] Yoo J., Park C., Yi G., Lee D., Koo H. (2019). Active Targeting Strategies Using Biological Ligands for Nanoparticle Drug Delivery Systems. Cancers.

[279] Leserman L.D., Barbet J., Kourilsky F.M., Weinstein J.N. (1980). Targeting to cells of fluorescent liposomes covalently coupled with monoclonal antibody or protein A. Nature.

[280] Senapati S., Mahanta A.K., Kumar S., Maiti P. (2018). Controlled drug delivery vehicles for cancer treatment and their performance. Signal Transduct. Target. Ther..

[281] Argenziano M., Arpicco S., Brusa P., Cavalli R., Chirio D., Dosio F., Gallarate M., Peira E., Stella B., Ugazio E. (2021). Developing Actively Targeted Nanoparticles to Fight Cancer: Focus on Italian Research. Pharmaceutics.

[282] Fang Z., Li X., Xu Z., Du F., Wang W., Shi R., Gao D. (2019). Hyaluronic acid-modified mesoporous silica-coated superparamagnetic Fe_3_O_4_ nanoparticles for targeted drug delivery. Int. J. Nanomed..

[283] Zhong S., Zhang H., Liu Y., Wang G., Shi C., Li Z., Feng Y., Cui X. (2017). Folic acid functionalized reduction-responsive magnetic chitosan nanocapsules for targeted delivery and triggered release of drugs. Carbohydr. Polym..

[284] Birlik Demirel G., Aygul E., Dag A., Atasoy S., Cimen Z., Cetin B. (2020). Folic Acid-Conjugated pH and Redox-Sensitive Ellipsoidal Hybrid Magnetic Nanoparticles for Dual-Triggered Drug Release. ACS Appl. Biol. Mater..

[285] Narmani A., Rezvani M., Farhood B., Darkhor P., Mohammadnejad J., Amini B., Refahi S., Abdi Goushbolagh N. (2019). Folic acid functionalized nanoparticles as pharmaceutical carriers in drug delivery systems. Drug Dev. Res..

[286] Nabavinia M.S., Gholoobi A., Charbgoo F., Nabavinia M., Ramezani M., Abnous K. (2017). Ant‰ MUC1 aptamer: A potential opportunity for cancer treatment. Med. Res. Rev..

[287] Siminzar P., Omidi Y., Golchin A., Aghanejad A., Barar J. (2020). Targeted delivery of doxorubicin by magnetic mesoporous silica nanoparticles armed with mucin-1 aptamer. J. Drug Target.

[288] Song H., Wang C., Zhang H., Yao L., Zhang J., Gao R., Tang X., Chong T., Liu W., Tang Y. (2019). A high-loading drug delivery system based on magnetic nanomaterials modified by hyperbranched phenylboronic acid for tumor-targeting treatment with pH response. Colloids Surf. B Biointerfaces.

[289] Song M.M., Xu H.L., Liang J.X., Xiang H.H., Liu R., Shen Y.X. (2017). Lactoferrin modified graphene oxide iron oxide nanocomposite for glioma-targeted drug delivery. Mater. Sci. Eng. C Mater. Biol. Appl..

[290] Ahmadi D., Zarei M., Rahimi M., Khazaie M., Asemi Z., Mir S.M., Sadeghpour A., Karimian A., Alemi F., Rahmati-Yamchi M. (2020). Preparation and in-vitro evaluation of pH-responsive cationic cyclodextrin coated magnetic nanoparticles for delivery of methotrexate to the Saos-2 bone cancer cells. J. Drug Deliv. Sci. Technol..

[291] Fathi M., Barar J., Erfan-Niya H., Omidi Y. (2020). Methotrexate-conjugated chitosan-grafted pH- and thermo-responsive magnetic nanoparticles for targeted therapy of ovarian cancer. Int. J. Biol. Macromol..

[292] Ak G., Aksu D., Çapkın E., Sarı Ö., Kımız Geboloğlu I., Şanlıer Ş. (2020). Delivery of pemetrexed by magnetic nanoparticles: Design, characterization,. Prep. Biochem. Biotechnology.

[293] Aghanejad A., Babamiri H., Adibkia K., Barar J., Omidi Y. (2018). Mucin-1 aptamer-armed superparamagnetic iron oxide nanoparticles for targeted delivery of doxorubicin to breast cancer cells. Bioimpacts.

[294] Reichel D., Tripathi M., Perez J.M. (2019). Biological effects of nanoparticles on macrophage polarization in the tumor microenvironment. Nanotheranostics.

[295] Karimi M., Ghasemi A., Zangabad P.S., Rahighi R., Basri S.M.M., Mirshekari H., Amiri M., Pishabad Z.S., Aslani A., Bozorgomid M. (2016). Smart micro/nanoparticles in stimulus-responsive drug/gene delivery systems. Chem. Soc. Rev..

[296] Stater E.P., Sonay A.Y., Hart C., Grimm J. (2021). The ancillary effects of nanoparticles and their implications for nanomedicine. Nat. Nanotechnol..

[297] Hossen S., Hossain M.K., Basher M., Mia M., Rahman M., Uddin M.J. (2019). Smart nanocarrier-based drug delivery systems for cancer therapy and toxicity studies: A review. J. Adv. Res..

[298] Tran S., DeGiovanni P.-J., Piel B., Rai P. (2017). Cancer nanomedicine: A review of recent success in drug delivery. Clin. Transl. Med..

[299] Wang X., Reece S.P., Brown J.M. (2013). Immunotoxicological impact of engineered nanomaterial exposure: Mechanisms of immune cell modulation. Toxicol. Mech. Methods.

[300] Escamilla-Rivera V., Solorio-Rodríguez A., Uribe-Ramírez M., Lozano O., Lucas S., Chagolla-López A., Winkler R., De Vizcaya-Ruiz A. (2019). Plasma protein adsorption on Fe_3_O_4_-PEG nanoparticles activates the complement system and induces an inflammatory response. Int. J. Nanomed..

[301] Behzadi S., Serpooshan V., Tao W., Hamaly M.A., Alkawareek M.Y., Dreaden E.C., Brown D., Alkilany A.M., Farokhzad O.C., Mahmoudi M. (2017). Cellular uptake of nanoparticles: Journey inside the cell. Chem. Soc. Rev..

[302] Luo L., Iqbal M.Z., Liu C., Xing J., Akakuru O.U., Fang Q., Li Z., Dai Y., Li A., Guan Y. (2019). Engineered nano-immunopotentiators efficiently promote cancer immunotherapy for inhibiting and preventing lung metastasis of melanoma. Biomaterials.

[303] Ferretti A.M., Usseglio S., Mondini S., Drago C., La Mattina R., Chini B., Verderio C., Leonzino M., Cagnoli C., Joshi P. (2020). Towards bio-compatible magnetic nanoparticles: Immune-related effects, in-vitro internalization, and in-vivo bio-distribution of zwitterionic ferrite nanoparticles with unexpected renal clearance. J. Colloid Interface Sci..

[304] Kruer M.C., Boddaert N. (2012). Neurodegeneration with brain iron accumulation: A diagnostic algorithm. Semin. Pediatric Neurol..

[305] Hauser A.K., Mitov M.I., Daley E.F., Mcgarry R., Anderson K.W., Hilt J.Z. (2016). Targeted iron oxide nanoparticles for the enhancement of radiation therapy. Biomaterials.

[306] Everett J., Collingwood J.F., Tjendana-Tjhin V., Brooks J., Lermyte F., Plascencia-Villa G., Hands-Portman I., Dobson J., Perry G., Telling N.D. (2018). Nanoscale synchrotron X-ray speciation of iron and calcium compounds in amyloid plaque cores from Alzheimer’s disease subjects. Nanoscale.

[307] Liu Q., Guan J., Song R., Zhang X., Mao S. (2021). Physicochemical properties of nanoparticles affecting their fate and the physiological function of pulmonary surfactants. Acta Biomater..

[308] Ruge C.A., Kirch J., Cañadas O., Schneider M., Pérez-Gil J., Schaefer U.F., Casals C., Lehr C.M. (2011). Uptake of nanoparticles by alveolar macrophages is triggered by surfactant protein A. Nanomed. Nanotechnol. Biol. Med..

[309] Li X., Guo X., Ling J., Tang Z., Huang G., He L., Chen T. (2021). Nanomedicine-based cancer immunotherapies developed by reprogramming tumor-associated macrophages. Nanoscale.

[310] Kashfi K., Kannikal J., Nath N. (2021). Macrophage Reprogramming and Cancer Therapeutics: Role of iNOS-Derived NO. Cells.

[311] Zhao Y., Muhetaerjiang M., An H.-W., Fang X., Zhao Y., Wang H. (2020). Nanomedicine enables spatiotemporally regulating macrophage-based cancer immunotherapy. Biomaterials.

[312] Liu X., Xie X., Jiang J., Lin M., Zheng E., Qiu W., Yeung I., Zhu M., Li Q., Xia T. (2021). Use of Nanoformulation to Target Macrophages for Disease Treatment. Adv. Funct. Mater..

[313] Nascimento C.S., Alves É.A.R., de Melo C.P., Corrêa-Oliveira R., Calzavara-Silva C.E. (2021). Immunotherapy for cancer: Effects of iron oxide nanoparticles on polarization of tumor-associated macrophages. Nanomedicine.

[314] Kodali V.K., Littke M.H., Tilton S.C., Teeguarden J.G., Shi L., Frevert C.W., Wang W., Pounds J.G., Thrall B.D. (2013). Dysregulation of macrophage activation profiles by engineered nanoparticles. ACS Nano.

[315] Lu Q., Dai X., Zhang P., Tan X., Zhong Y., Yao C., Song M., Song G., Zhang Z., Peng G. (2018). Fe_3_O_4_@Au composite magnetic nanoparticles modified with cetuximab for targeted magneto-photothermal therapy of glioma cells. Int. J. Nanomed..

[316] Anselmo A.C., Mitragotri S. (2015). A Review of Clinical Translation of Inorganic Nanoparticles. AAPS J..

[317] (2022). Clinical Trial. https://www.clinicaltrials.gov/.

[318] Singh D., McMillan J.M., Kabanov A.V., Sokolsky-Papkov M., Gendelman H.E. (2014). Bench-to-bedside translation of magnetic nanoparticles. Nanomedicine.

[319] Maier-Hauff K., Ulrich F., Nestler D., Niehoff H., Wust P., Thiesen B., Orawa H., Budach V., Jordan A. (2010). Efficacy and safety of intratumoral thermotherapy using magnetic iron-oxide nanoparticles combined with external beam radiotherapy on patients with recurrent glioblastoma multiforme. J. Neuro-Oncol..

[320] Min Y., Caster J.M., Eblan M.J., Wang A.Z. (2015). Clinical Translation of Nanomedicine. Chem. Rev..

[321] Tekade R.K. (2018). Basic Fundamentals of Drug Delivery.

[322] Guo S., Huang L. (2011). Nanoparticles escaping RES and endosome: Challenges for siRNA delivery for cancer therapy. J. Nanomater..

[323] Zhang X., Chen X., Guo Y., Jia H.-R., Jiang Y.W., Wu F.G. (2020). Endosome/lysosome-detained supramolecular nanogels as an efflux retarder and autophagy inhibitor for repeated photodynamic therapy of multidrug-resistant cancer. Nanoscale Horiz..

[324] Schleich N., Danhier F., Préat V. (2015). Iron oxide-loaded nanotheranostics: Major obstacles to in vivo studies and clinical translation. J. Control. Release.

[325] Ross K.A., Brenza T.M., Binnebose A.M., Phanse Y., Kanthasamy A.G., Gendelman H.E., Salem A.K., Bartholomay L.C., Bellaire B.H., Narasimhan B. (2015). Nano-enabled delivery of diverse payloads across complex biological barriers. J. Control. Release Off. J. Control. Release Soc..

[326] Landry R.S., Jacobs P.M., Davis R., Shenouda M., Bolton W.K. (2005). Pharmacokinetic Study of Ferumoxytol: A New Iron Replacement Therapy in Normal Subjects and Hemodialysis Patients. Am. J. Nephrol..

[327] Feng Q., Liu Y., Huang J., Chen K., Huang J., Xiao K. (2018). Uptake, distribution, clearance, and toxicity of iron oxide nanoparticles with different sizes and coatings. Sci. Rep..

[328] Gao Y., Lim J., Teoh S.-H., Xu C. (2015). Emerging translational research on magnetic nanoparticles for regenerative medicine. Chem. Soc. Rev..

[329] Barré-Sinoussi F.o., Montagutelli X. (2015). Animal models are essential to biological research: Issues and perspectives. Future Sci. OA.

[330] Pound P., Ritskes-Hoitinga M. (2018). Is it possible to overcome issues of external validity in preclinical animal research? Why most animal models are bound to fail. J. Transl. Med..

